# The global, regional, and national burden of stomach cancer in 195 countries, 1990–2017: a systematic analysis for the Global Burden of Disease study 2017

**DOI:** 10.1016/S2468-1253(19)30328-0

**Published:** 2019-10-21

**Authors:** GBD 2017 Stomach Cancer Collaborators, Arash Etemadi, Saeid Safiri, Sadaf G Sepanlou, Kevin Ikuta, Catherine Bisignano, Ramin Shakeri, Mohammad Amani, Christina Fitzmaurice, Molly Nixon, Nooshin Abbasi, Hassan Abolhassani, Shailesh M Advani, Mohsen Afarideh, Tomi Akinyemiju, Tahiya Alam, Mahtab Alikhani, Vahid Alipour, Christine A Allen, Amir Almasi-Hashiani, Jalal Arabloo, Reza Assadi, Suleman Atique, Ashish Awasthi, Ahad Bakhtiari, Masoud Behzadifar, Kidanemaryam Berhe, Neeraj Bhala, Ali Bijani, Muhammad Shahdaat Bin Sayeed, Tone Bjørge, Antonio M Borzì, Dejana Braithwaite, Hermann Brenner, Giulia Carreras, Félix Carvalho, Carlos A Castañeda-Orjuela, Franz Castro, Dinh-Toi Chu, Vera M Costa, Ahmad Daryani, Dragos V Davitoiu, Gebre T Demoz, Asmamaw B Demis, Edgar Denova-Gutiérrez, Subhojit Dey, Mostafa Dianati Nasab, Shirin Djalalinia, Mohammad Hassan Emamian, Mohammad Farahmand, João C Fernandes, Florian Fischer, Masoud Foroutan, Mohamed M Gad, Silvano Gallus, Gebreamlak Gebremedhn Gebremeskel, Getnet A Gedefew, Fatemeh Ghaseni-Kebria, Giuseppe Gorini, Nima Hafezi-Nejad, Arvin Haj-Mirzaian, Josep M Haro, James D Harvey, Amir Hasanzadeh, Maryam Hashemian, Hamid Y Hassen, Simon I Hay, Hagos D Hidru, Mihaela Hostiuc, Mowafa Househ, Olayinka s Ilesanmi, Milena D. Ilic, Kaire Innos, Farhad Islami, Spencer L James, Ensiyeh Jenabi, Rohollah kalhor, Farin Kamangar, Amir Kasaeian, Andre P Kengne, Yousef S Khader, Rovshan Khalilov, Ejaz A Khan, Gulfaraz Khan, Maryam Khayamzadeh, Maryam Khazaee-Pool, Salman Khazaei, Abdullah T Khoja, Fatemah Khosravi Shadmani, Yun Jin Kim, Jonathan M Kocarnik, Hamidreza Komaki, Ai Koyanagi, Vivek Kumar, Carlo La Vecchia, Alan D Lopez, Raimundas Lunevicius, Navid Manafi, Ana-Laura Manda, Birhanu Geta, Hailemariam Meheretu, Getnet Mengistu, Bartosz Miazgowski, Seyed Mostafa Mir, Karzan A Mohammad, Naser Mohammad Gholi Mezerji, Mahdi Mohammadian, Abdollah Mohammadian-Hafshejani, Reza Mohammadpourhodki, Shafiu Mohammed, Farnam Mohebi, Ali H Mokdad, Lorenzo Monasta, Mahmood Moosazadeh, Maryam Moossavi, Ghobad Moradi, Farhad Moradpour, Rahmatollah Moradzadeh, Ilais Moreno Vel squez, Abbas Mosapour, Mehdi Naderi, Gurudatta Naik, Farid Najafi, Azin Nahvijou, Ionut Negoi, Rajan Nikbakhsh, Marzieh Nojomi, Andrew T Olagunju, Tinuke O Olagunju, Eyal Oren, Hadi Parsian, Cristiano Piccinelli, Akram Pourshams, Hossein Poustchi, Navid Rabiee, Amir Radfar, Alireza Rafiei, Mahdi Rahimi, Marveh Rahmati, Andre M N Renzaho, Nima Rezaei, Ana Isabel Ribeiro, Gholamreza Roshandel, Anas M Saad, Seyedmohammad Saadatagah, Hamideh Salimzadeh, Abdallah M Samy, Juan Sanabria, Milena M Santric Milicevic, Arash Sarveazad, Monika Sawhney, Faramarz Shaahmadi, Mario Sekerija, Masood A Shaikh, Amir Shamshirian, Sudeep K Siddappa Malleshappa, Jasvinder A Singh, Catalin-Gabriel Smarandache, Moslem Soofi, Takahiro Tabuchi, Degena B Tadesse, Leili Tapak, Berhe E Tesfay, Eugenio Traini, Bach Tran, Khanh B Tran, Marco Vacante, Amir Vahedian-Azimi, Yousef Veisani, Kia Vosoughi, Isidora S Vujcic, Ronny Westerman, Adam B Wondmieneh, Rixing Xu, Sanni Yaya, Vahid Yazdi-Feyzabadi, Zabihollah Yousefi, Bhaman Yousefi, Telma Zahirian Moghadam, Leila Zaki, Mohammad Zamani, Maryam Zamanian, Hamed Zandian, Afshin Zarghi, Zhi-Jiang Zhang, Mohsen Naghavi, Reza Malekzadeh

## Abstract

**Background:**

Stomach cancer is a major health problem in many countries. Understanding the current burden of stomach cancer and the differential trends across various locations is essential for formulating effective preventive strategies. We report on the incidence, mortality, and disability-adjusted life-years (DALYs) due to stomach cancer in 195 countries and territories from 21 regions between 1990 and 2017.

**Methods:**

Estimates from GBD 2017 were used to analyse the incidence, mortality, and DALYs due to stomach cancer at the global, regional, and national levels. The rates were standardised to the GBD world population and reported per 100 000 population as age-standardised incidence rates, age-standardised death rates, and age-standardised DALY rates. All estimates were generated with 95% uncertainty intervals (UIs).

**Findings:**

In 2017, more than 1·22 million (95% UI 1·19–1·25) incident cases of stomach cancer occurred worldwide, and nearly 865 000 people (848 000–885 000) died of stomach cancer, contributing to 19·1 million (18·7–19·6) DALYs. The highest age-standardised incidence rates in 2017 were seen in the high-income Asia Pacific (29·5, 28·2–31·0 per 100 000 population) and east Asia (28·6, 27·3–30·0 per 100 000 population) regions, with nearly half of the global incident cases occurring in China. Compared with 1990, in 2017 more than 356 000 more incident cases of stomach cancer were estimated, leading to nearly 96 000 more deaths. Despite the increase in absolute numbers, the worldwide age-standardised rates of stomach cancer (incidence, deaths, and DALYs) have declined since 1990. The drop in the disease burden was associated with improved Socio-demographic Index. Globally, 38·2% (21·1–57·8) of the age-standardised DALYs were attributable to high-sodium diet in both sexes combined, and 24·5% (20·0–28·9) of the age-standardised DALYs were attributable to smoking in males.

**Interpretation:**

Our findings provide insight into the changing burden of stomach cancer, which is useful in planning local strategies and monitoring their progress. To this end, specific local strategies should be tailored to each country's risk factor profile. Beyond the current decline in age-standardised incidence and death rates, a decrease in the absolute number of cases and deaths will be possible if the burden in east Asia, where currently almost half of the incident cases and deaths occur, is further reduced.

**Funding:**

Bill & Melinda Gates Foundation.

## Introduction

Stomach cancer is an important contributor to the global burden of cancer,^1^ and less than a century ago it was the most common cancer in the world.^2^ Since then, the incidence and mortality rates of stomach cancer have fallen.^3^ However, this trend has shown signs of change; for example, some researchers suggest that in the USA, the rates of stomach cancer might be increasing among younger age groups (ie, <50 years) and predict that this increase might reverse the overall decline in the incidence of stomach cancer.^4^ More than 90% of stomach cancers are adenocarcinomas, and, depending on whether the tumour is located near the gastro-oesophageal junction (cardia) or away from it, they are subdivided into cardia and non-cardia tumours, respectively.^1^ The decreasing trend of stomach cancer incidence and mortality in most populations is due to the falling rates of non-cardia stomach cancer and has been linked to a decline in *Helicobacter pylori* infection rates.^5^, ^6^ *H pylori* is a known carcinogen^7^ for non-cardia stomach cancer, which probably once infected most adults during their life course.^8^ Improved socio-economic status, hygienic practices, and widespread antibiotic use have led to a decrease in infection rates.^9^

The epidemiology of stomach cancer has substantial geographical heterogeneity, and its incidence can vary 5-fold to 10-fold between high-risk and low-risk countries.^10^ Part of this geographical variation correlates with *H pylori* infection rates across populations; however, a number of environmental factors also contribute to the risk of stomach cancer. Cigarette smoking has been shown to be a risk factor for both cardia and non-cardia stomach cancers.^11^ Evidence suggests that salt and salt-preserved foods might increase the risk of stomach cancer.^12^, ^13^ Both types of stomach cancer are more common among males, which might be due to the higher prevalence of risk factors, such as smoking, or hormonal factors contributing to this difference.^2^

**Research in context**

**Evidence before this study**

The age-standardised incidence and death rates of stomach cancer have declined in most parts of the world, but it remains a major health problem in many countries. Understanding the current burden of stomach cancer and trends across different locations is essential for formulating effective preventive strategies. The International Agency for Research on Cancer has regularly provided cancer estimates in the Global Cancer Incidence, Mortality and Prevalence (GLOBOCAN) project; however, GLOBOCAN does not provide estimates over time for all locations, correlations with risk factors, or estimates for disability-adjusted life-years (DALYs). We used estimates from the Global Burden of Diseases, Injuries, and Risk Factors Study (GBD) 2017 to examine trends of incidence, mortality, and burden of disease across 195 countries and territories in seven super-regions and 21 regions from 1990 to 2017.

**Added value of this study**

Using results from GBD 2017, we studied the global, regional, and national trends of stomach cancer using comprehensive data collected from sources around the world, and reported geographical variation and trends over time. Our study adds value to the available evidence because, to our knowledge, it provides the most comprehensive assessment of the burden of stomach cancer by age, sex, socio-demographic status, and location over time.

**Implications of all the available evidence**

Stomach cancer is a significant cause of morbidity and mortality in many parts or the world, and the total numbers of incident cases and deaths are increasing worldwide. East Asia, particularly China, contributes the largest number of incident cases, deaths, and DALYs of stomach cancer in the world. The age-standardised rates of incidence and death, however, have declined steadily, and are much lower than the corresponding rates in 1990. The falling rates globally, and within each region, are associated with rises in the Socio-demographic Index. Our findings provide insight into the changing burden of stomach cancer at the global, regional, and national levels, which can be used by policy makers to develop location-specific programmes aimed at further reducing the burden of stomach cancer.

Although survival rates have generally improved over the past several decades, prognosis remains poor.^14^ The 5-year survival rate is around 20%, with the notable exceptions of 65% in Japan^15^ and 71·5% in South Korea,^16^ where population screening has led to the effective diagnosis of tumours at early stages.^17^ Given this poor survival and the considerable burden associated with stomach cancer, we evaluated the burden of stomach cancer using the results of the Global Burden of Diseases, Injuries, and Risk Factors Study (GBD) 2017. We highlight global and regional trends that can help to inform global and local interventions to lower disease burden and, perhaps, curtail the increasing number of incident cases.

## Methods

### Overview

The methods used for GBD 2017 have been described previously and are briefly summarised here.^18^, ^19^, ^20^, ^21^ Cancers in GBD 2017 are classified into 29 groups according to the International Classification of Diseases 10th edition (ICD-10). Stomach cancer included all diagnoses coded C16·0–C16·9 (malignant neoplasm of stomach), Z12·0, and Z85·02–Z85·028, and did not include tumours of gastro-oesophageal junction.^22^ This study is compliant with the Guidelines for Accurate and Transparent Health Estimates Reporting.

### Data sources

For this study, we used GBD 2017 vital registration (19 618 site-years of data), verbal autopsy (374 site-years), and cancer registry (4474 site-years) data sources that provided a representative partial or complete sample of incidence or mortality. Information about the data sources used for each location in this study can be found on the GBD 2017 Data Input Sources Tool website.

### Mortality

We derived mortality estimates from the data source described above and, when necessary, registry incidence data were multiplied by the corresponding, independently modelled, mortality-to-incidence ratio to produce mortality estimates. Mortality-to-incidence ratios were modelled using locations where same-year cancer mortality and incidence data were available. The mortality-to-incidence ratio model started with a linear-step mixed-effects model with logit link functions, with the Healthcare Access and Quality (HAQ) Index,^23^ age, and sex as covariates. The estimates produced by this model were then smoothed over space and time and adjusted using spatiotemporal Gaussian process regression.^24^ All the estimates computed from the mortality-to-incidence ratios and incidence data were used as inputs for a Cause of Death Ensemble model.^25^ Potential covariates in these models were selected at three levels, pertaining to a possible predictive relationship between the covariate and stomach cancer mortality. Level 1 covariates (those with a proven strong relationship with stomach cancer; eg, biological or causative link) included smoking prevalence, mean cigarettes per capita, cumulative cigarettes (5, 10, 15, and 20 mean pack-years), diet high in sodium, log-transformed summary exposure value (SEV) scalar for stomach cancer, SEV of unsafe water, and SEV of unsafe sanitation. Level 2 covariates (strong relationship without a direct biological link) were mean body-mass index, indoor air pollution (all cooking fuels), outdoor air pollution (particulate matter concentration of 2·5 μm dimeter), HAQ Index, adjusted fruit and vegetable intake (grams), sanitation (proportion with access), and improved water source (proportion with access). Finally, Level 3 (more distal covariates mediated through Level 1 or 2 covariates) included education (years per capita), lag-distributed income (US$ per capita), and Socio-demographic Index (SDI).

### Non-fatal modelling

The final mortality estimates were divided by the estimated mortality-to-incidence ratios to compute stomach cancer incidence. Disability-adjusted life-years (DALYs) were calculated by summing years lived with disability (YLDs) and years of life lost (YLLs). The contributions of YLDs and YLLs to stomach cancer DALYs were 2% and 98%, respectively. YLDs were estimated by classifying 10-year cancer prevalence into four sequelae and multiplying these prevalences by corresponding disability weights: diagnosis and treatment, remission, disseminated and metastatic, and terminal phase. The durations of the four prevalence phases for stomach cancer were 5·2 months^26^ of diagnosis and treatment, 3·88 months^27^ of disseminated and metastatic disease, and 1 month of terminal phase. Remission durations were calculated on the basis of the remainder of time after attributing other sequelae. The YLLs were estimated by multiplying the estimated number of deaths by age with a standard life expectancy at that age. Details of estimation methods and data sources have been published before.^22^

Rates were standardised to the GBD world population and reported per 100 000 population as age-standardised incidence rates, age-standardised death rates, and age-standardised DALY rates. All estimates were generated with 95% uncertainty intervals (UIs), including all sources of uncertainty arising from measurement error, biases, and modelling. The 95% UIs were derived from the 2·5th and 97·5th percentiles of 1000 draws.

### SDI and risk factors

Risk factor quantification was based on the GBD 2017 comparative risk assessment described earlier.^18^ The SDI is a composite indicator of socio-development status that includes fertility, education, and income, and which has shown a strong association with health outcomes. SDI ranges from zero (worst) to one (best).^22^ We used linear correlation and fitted regression lines to determine the relationship between countries' development level (ie, SDI) and incidence, death, and DALY rates of stomach cancer. We reported the percentage of DALYs due to stomach cancer that were attributable to high-sodium diet and smoking by multiplying stomach cancer DALYs by the risk factor's population attributable fraction for a given age, sex, location, and year.^18^

### Role of the funding source

The funder of the study had no role in study design, data collection, data analysis, data interpretation, or the writing of the report. All authors had full access to the data in the study and had final responsibility for the decision to submit for publication.

## Results

**Figure 1:**
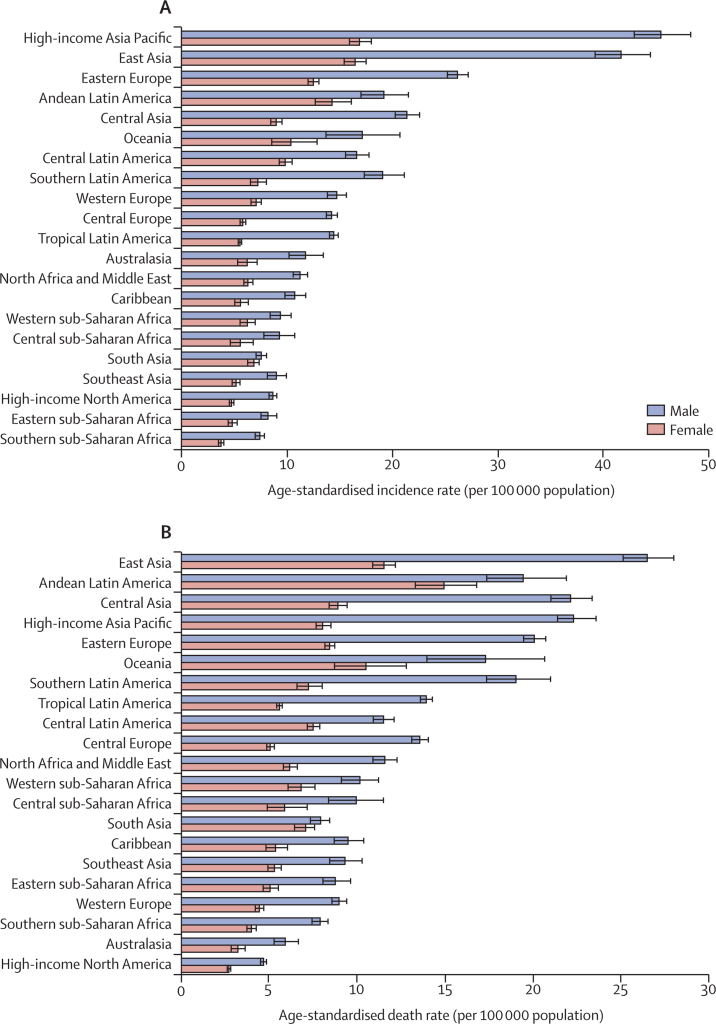
The age-standardised incidence (A) and death (B) rates of stomach cancer in 2017 for 21 GBD regions, by sex GBD=Global Burden of Diseases, Injuries, and Risk Factors Study.

In 2017, more than 1·22 million (95% UI 1·19–1·25) incident cases of stomach cancer occurred worldwide, and nearly 865 000 people (848 000–885 000) died of stomach cancer (table). The age-standardised incidence rate of stomach cancer was 15·4 per 100 000 population (15·0–15·8), with an age-standardised death rate of 11·0 per 100 000 population (10·8–11·2). Stomach cancer contributed to 19·1 million (18·7–19·6) DALYs worldwide in 2017. For males, both the age-standardised incidence and death rates of stomach cancer were more than twice the rates for females (21·7 [21·0–22·6] *vs* 9·9 [9·6–10·2], and 15·2 [14·8–15·7] *vs* 7·5 [7·3–7·7] per 100 000 population, respectively). The male–female incidence and death gap existed in all regions, but was narrower in Andean Latin America and south Asia (figure 1). Specific country and territory data for incidence, deaths, and DALYs can be found in the appendix (p 2–25).

**Table:** Incident cases of deaths and DALYs of stomach cancer in 2017, and percentage change of age-standardised rates by sex and GBD region

	**Incidence**			**Deaths**			**DALYs**		
	Number of incident cases	Age-standardised incidence rate (per 100 000 population)	Percentage change in rates, 1990–2017	Number of deaths	Age-standardised death rate (per 100 000 population)	Percentage change in rates, 1990–2017	Number of DALYs	Age-standardised DALYs rate (per 100 000 population)	Percentage change in rates, 1990–2017
Global	1 220 662 (1 189 032 to 1 254 563)	15·4 (15·0 to 15·8)	–28·0 (−30·5 to −25·4)	864 989 (848 254 to 884 655)	11·0 (10·8 to 11·2)	–43·2 (−45·1 to −41·4)	19 130 771 (18 738 585 to 19 569 409)	235·9 (231·1 to 241·3)	–47·1 (−49·0 to −45·3)
Males	799 309 (771 025 to 830 413)	21·7 (21·0 to 22·6)	–24·0 (−27·0 to −20·7)	546 441 (530 918 to 564 028)	15·2 (14·8 to 15·7)	–41·4 (−43·4 to −38·9)	12 248 716 (11 898 092 to 12 645 097)	317·8 (308·8 to 327·9)	–45·4 (−47·6 to −43·0)
Females	421 353 (408 084 to 434 424)	9·9 (9·6 to 10·2)	–35·6 (−39·3 to −32·3)	318 548 (309 796 to 327 854)	7·5 (7·3 to 7·7)	–46·9 (−50·0 to −44·4)	6 882 055 (6 683 617 to 7 083 864)	163·0 (158·3 to 167·8)	–50·0 (−53·0 to −47·5)
Andean Latin America	8925 (8194 to 9658)	16·6 (15·2 to 18·0)	–33·2 (−39·2 to −26·6)	9130 (8408 to 9901)	17·1 (15·7 to 18·5)	–35·9 (−41·4 to −29·7)	193 905 (176 702 to 210 939)	353·5 (322·4 to 384·4)	–38·3 (−44·2 to −31·9)
Australasia	4263 (3845 to 4692)	8·8 (7·9 to 9·7)	–24·9 (−32·4 to −17·1)	2233 (2052 to 2429)	4·4 (4·1 to 4·8)	–47·4 (−51·7 to −42·8)	39 703 (36 268 to 43 401)	88·4 (80·7 to 96·6)	–47·6 (−52·2 to −42·5)
Caribbean	4033 (3769 to 4342)	7·9 (7·4 to 8·5)	–25·2 (−30·4 to −19·6)	3684 (3438 to 3966)	7·3 (6·8 to 7·8)	–34·1 (−38·6 to −29·3)	83 446 (77 493 to 90 753)	164·4 (152·5 to 178·7)	–31·1 (−36·2 to −25·2)
Central Asia	10 513 (10 059 to 10 965)	14·1 (13·5 to 14·7)	–38·9 (−41·6 to −36·2)	10 331 (9891 to 10 769)	14·3 (13·8 to 14·9)	–39·0 (−41·7 to −36·4)	273 093 (260 966 to 285 850)	340·0 (325·0 to 354·6)	–42·7 (−45·3 to −40·1)
Central Europe	19 794 (19 194 to 20 462)	9·4 (9·1 to 9·7)	–43·3 (−45·4 to −41·2)	18 570 (18 014 to 19 135)	8·6 (8·4 to 8·9)	–48·7 (−50·5 to −46·8)	379 211 (367 809 to 391 374)	189·9 (184·0 to 196·0)	–50·0 (−51·8 to −48·0)
Central Latin America	29 601 (28 255 to 31 008)	12·9 (12·3 to 13·5)	–16·9 (−20·8 to −12·7)	21 226 (20 432 to 22 007)	9·3 (9·0 to 9·7)	–41·6 (−43·8 to −39·4)	487 298 (468 124 to 506 160)	203·8 (195·8 to 211·7)	–38·9 (−41·4 to −36·6)
Central sub-Saharan Africa	3555 (3106 to 4009)	7·1 (6·3 to 8·0)	–34·3 (−43·2 to −24·4)	3551 (3110 to 3990)	7·6 (6·7 to 8·5)	–33·9 (−42·7 to −24·2)	100 529 (87 599 to 113 903)	173·0 (151·3 to 195·0)	–34·9 (−44·5 to −24·0)
East Asia	583 758 (554 933 to 612 688)	28·6 (27·3 to 30·0)	–14·7 (−21·5 to −8·9)	371 288 (356 398 to 387 201)	18·7 (17·9 to 19·5)	–44·5 (−48·7 to −41·0)	8 175 270 (7 834 190 to 8 543 708)	389·5 (373·5 to 407·2)	–49·7 (−53·3 to −46·6)
Eastern Europe	59 809 (57 983 to 61 663)	17·7 (17·2 to 18·3)	–39·5 (−41·4 to −37·5)	43 943 (42 870 to 45 173)	12·8 (12·5 to 13·1)	–51·9 (−52·9 to −50·8)	1 014 257 (987 340 to 1 043 869)	308·7 (300·7 to 317·3)	–53·5 (−54·5 to −52·5)
Eastern sub-Saharan Africa	10 056 (9361 to 10 804)	6·4 (5·9 to 6·8)	–40·2 (−46·5 to −32·8)	10 087 (9391 to 10 828)	6·8 (6·3 to 7·3)	–39·3 (−45·3 to −32·5)	280 901 (261 364 to 301 544)	155·8 (145·0 to 167·4)	–41·9 (−48·7 to −33·7)
High-income Asia Pacific	131 636 (125 691 to 138 437)	29·5 (28·2 to 31·0)	–48·7 (−51·1 to −45·9)	68 042 (65 688 to 71 099)	14·2 (13·7 to 14·8)	–56·7 (−58·3 to −54·7)	1 099 094 (1 056 842 to 1147 281)	280·0 (268·6 to 292·3)	–62·4 (−63·8 to −60·6)
High-income North America	39 247 (37 998 to 40 539)	6·5 (6·3 to 6·7)	–20·4 (−23·3 to −17·4)	22 159 (21 591 to 22 750)	3·6 (3·5 to 3·7)	–40·0 (−41·7 to −38·3)	419 819 (407 273 to 431 498)	74·5 (72·3 to 76·7)	–39·2 (−41·3 to −37·2)
North Africa and Middle East	35 755 (33 988 to 37 539)	8·7 (8·3 to 9·1)	–35·6 (−40·7 to −29·7)	34 530 (32 838 to 36 201)	8·8 (8·4 to 9·2)	–37·4 (−42·1 to −31·9)	857 927 (809 034 to 906 399)	189·0 (179·0 to 198·9)	–42·2 (−46·9 to −36·5)
Oceania	988 (815 to 1177)	13·8 (11·7 to 16·1)	–14·5 (−25·6 to −3·0)	913 (758 to 1077)	14·0 (11·9 to 16·1)	–14·5 (−24·8 to −3·4)	30 288 (24 766 to 36 707)	358·4 (297·2 to 423·6)	–15·4 (−28·2 to −1·9)
South Asia	96 577 (91 436 to 101 062)	7·2 (6·8 to 7·5)	–30·9 (−37·2 to −25·0)	96 652 (91 276 to 101 052)	7·5 (7·0 to 7·8)	–30·2 (−36·6 to −24·6)	2 626 634 (2 487 690 to 2 747 242)	178·2 (168·5 to 186·2)	–33·0 (−39·4 to −27·3)
Southeast Asia	39 191 (36 559 to 42 265)	6·8 (6·4 to 7·3)	–43·8 (−49·0 to −38·2)	38 871 (36 283 to 41 869)	7·0 (6·6 to 7·6)	–44·7 (−49·7 to −39·4)	960 904 (893 460 to 1038 037)	154·1 (143·5 to 166·3)	–48·1 (−53·3 to −42·3)
Southern Latin America	10 145 (9436 to 10 956)	12·4 (11·5 to 13·4)	–34·1 (−39·0 to −28·5)	10 203 (9515 to 10 988)	12·3 (11·5 to 13·2)	–38·3 (−42·8 to −33·1)	203 392 (188 154 to 220 430)	253·8 (234·7 to 275·1)	–40·4 (−45·0 to −35·0)
Southern sub-Saharan Africa	2839 (2706 to 2979)	5·2 (5·0 to 5·4)	–27·3 (−32·3 to −22·2)	2910 (2779 to 3052)	5·5 (5·3 to 5·8)	–26·2 (−31·3 to −21·0)	73 206 (69 472 to 77 098)	123·3 (117·3 to 129·6)	–30·7 (−35·8 to −25·5)
Tropical Latin America	21 823 (21 369 to 22 331)	9·5 (9·3 to 9·7)	–40·2 (−41·8 to −38·5)	21 140 (20 734 to 21 550)	9·3 (9·1 to 9·4)	–44·9 (−46·3 to −43·6)	482 998 (473 844 to 492 741)	203·0 (199·3 to 207·1)	–44·1 (−45·6 to −42·8)
Western Europe	95 266 (90 462 to 100 111)	10·5 (10·0 to 11·0)	–37·4 (−40·6 to −34·2)	62 213 (59 851 to 64 525)	6·4 (6·2 to 6·7)	–54·3 (−56·0 to −52·6)	1 025 787 (984 851 to 1 066 633)	125·4 (120·2 to 130·4)	–55·1 (−56·9 to −53·3)
Western sub-Saharan Africa	12 890 (11 719 to 14 193)	7·7 (7·1 to 8·5)	–20·5 (−27·9 to −11·3)	13 311 (12 153 to 14 701)	8·4 (7·7 to 9·2)	–19·5 (−27·1 to −9·9)	323 109 (293 082 to 359 119)	167·9 (152·9 to 185·7)	–23·8 (−30·9 to −14·8)

Data in parentheses are 95% uncertainty intervals. DALY=disability-adjusted life-year. GBD=Global Burden of Diseases, Injuries, and Risk Factors Study.

**Figure 2:**
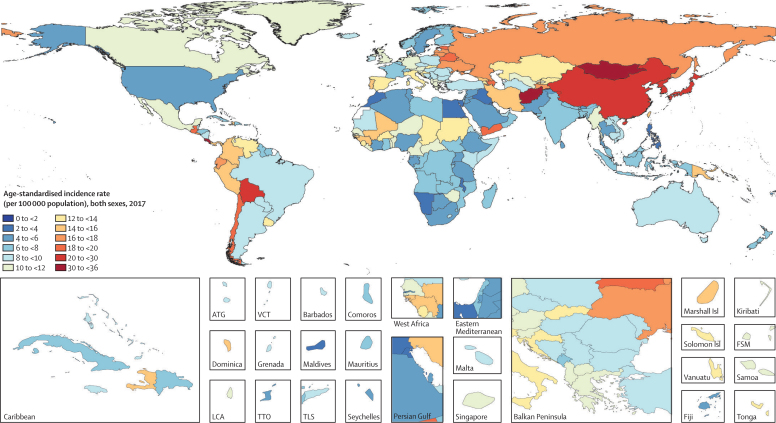
Age-standardised incidence rate of stomach cancer per 100 000 population in 2017, for 195 countries and territories

The world map of age-standardised incidence rates of stomach cancer in 2017 is shown in figure 2. The highest age-standardised incidence rate was seen in the high-income Asia Pacific region (29·5 per 100 000 population [95% UI 28·2–31·0]), particularly in Japan and South Korea, and east Asia (28·6 per 100 000 population [27·3–30·0]; table; appendix pp 2–9, 18–25). In east Asia, China alone had nearly half of the global incident cases in 2017 (562 000 [533 000–591 000]), and contributed to 7·8 million (7·5–8·2) DALYs (appendix pp 2–9, 18–25). The eastern Europe (17·7 [17·2–18·3]) and Andean Latin America (16·6 [15·2–18·0]) regions had the next highest age-standardised incidence rates. Two countries outside these high-incidence regions—Mongolia (35·6 [31·9–39·6]) and Afghanistan (32·8, [26·5–39·6])—had the overall highest age-standardised incidence rates. The lowest incidence rates were seen in southern and eastern sub-Saharan Africa and high-income North America (table). East Asia had the highest age-standardised death rate (18·7 [17·9–19·5]), followed by Andean Latin America (17·1 [15·7–18·5]) and central Asia (14·3 [13·8–14·9]). The high-income Asia Pacific region, which ranked first in age-standardised incidence rate, had the fourth highest age-standardised death rate and the sixth highest DALY rate among all GBD regions in 2017. The two countries with the highest age-standardised incidence rate also had the highest age-standardised death rates: Mongolia (37·6 [33·8–41·8]) and Afghanistan (33·6 [27·2–40·2]). The lowest age-standardised death rates were seen in high-income North America and Australasia.

**Figure 3:**
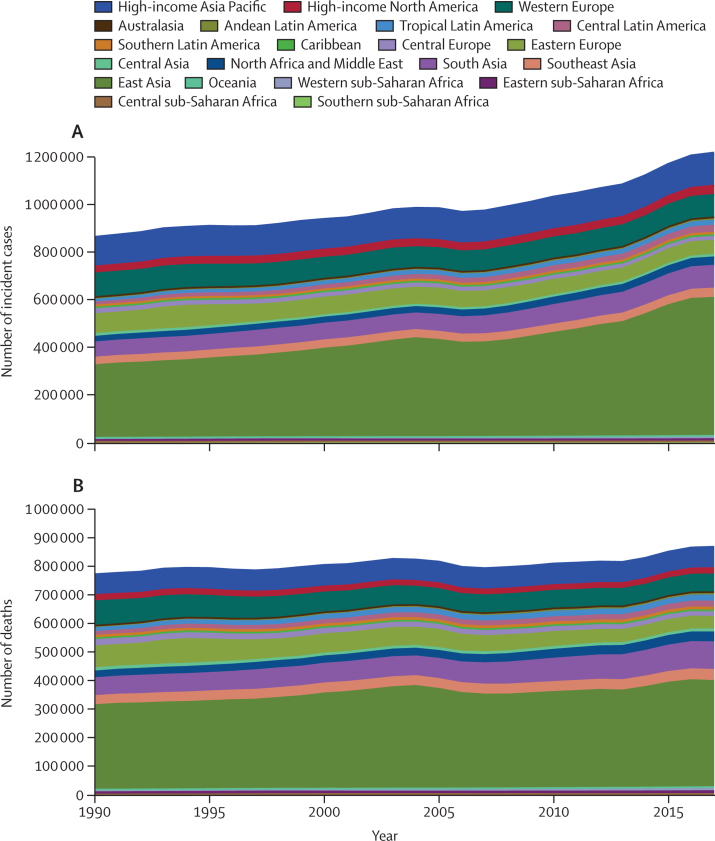
Absolute number of incident cases of (A) and deaths due to (B) stomach cancer, 1990 to 2017, for 21 GBD regions GBD=Global Burden of Diseases, Injuries, and Risk Factors Study.

Compared with 1990, in 2017 the number of incident cases of stomach cancer increased from about 864 000 (95% UI 847 000–890 000) to 1·22 million (1·19–1·25; appendix p 2)—an increase of around 356 000 cases. The number of deaths increased from around 769 000 (752 000–795 000) to 865 000 (848 000–885 000; appendix p 10)—ie, an increase of around 96 000 deaths. But stomach cancer contributed to almost the same number of DALYs in 2017 as in 1990 (appendix pp 18–25). The majority of increases in the absolute number of cases and deaths came from east Asia: from 1990 to 2017, incident cases rose from about 308 000 (296 000–326 000) to almost 584 000 (555 000–613 000) and the number of deaths increased from around 296 000 (285 000–313 000) to more than 371 000 (356 000–387 000). Again, the bulk of these increases occurred in China (appendix pp 2–17). Other regions that made large contributions to increased numbers of cases and deaths included south Asia, central Latin America, and north Africa and the Middle East (figure 3). In high-income Asia Pacific countries, the number of incident cases increased from over 117 000 (115 000–119 000) to almost 132 000 (126 000–138 000), but the number of deaths due to stomach cancer showed very little change in the same period (66 000 [65 300–66 800] in 1990 to 68 000 [65 700–71 100] in 2017; appendix pp 2–17). Both the number of incident cases and number of deaths due to stomach cancer decreased in European regions (central, eastern, and western) between 1990 and 2017.

**Figure 4:**
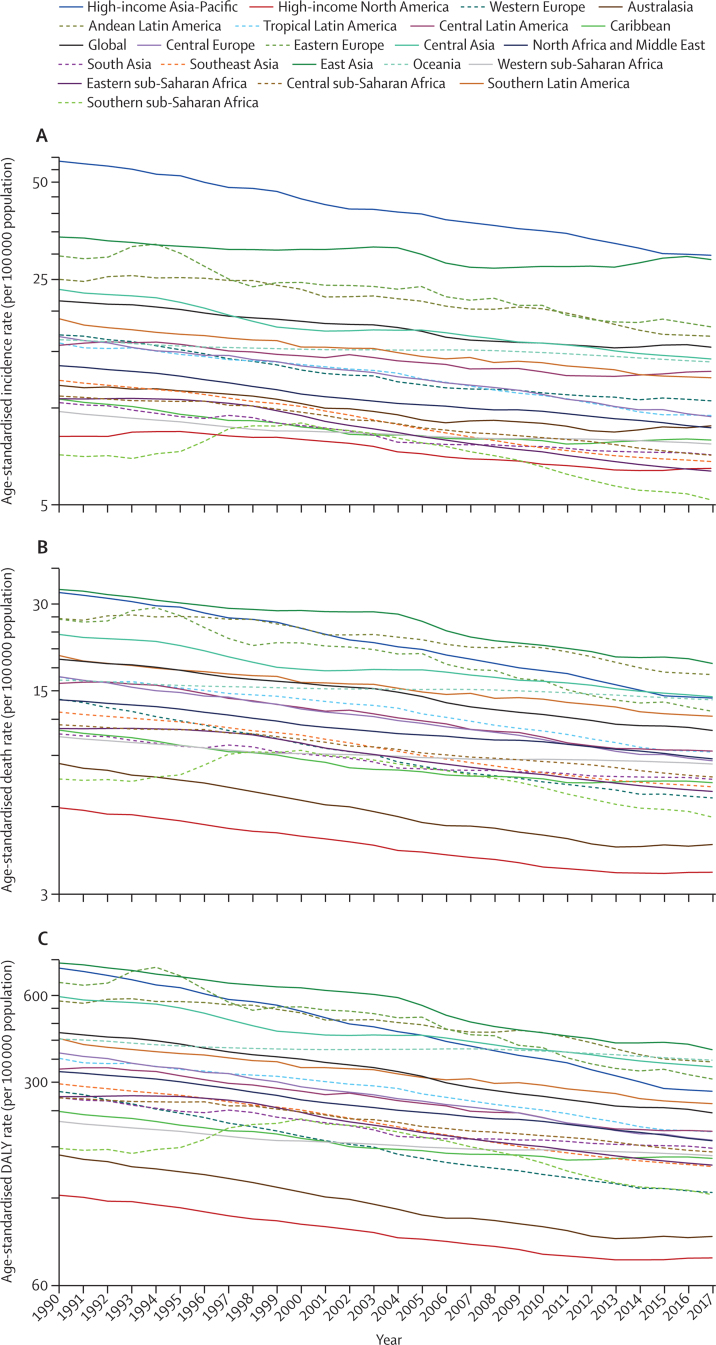
Secular trends of age-standardised incidence (A), death (B), and DALY (C) rates of stomach cancer, 1990–2017, global and for 21 GBD regions DALYs=disability-adjusted life-years. GBD=Global Burden of Diseases, Injuries, and Risk Factors Study.

Despite the increases in absolute numbers, the global age-standardised incidence and death rates of stomach cancer decreased compared with 1990 (figure 4). During this period, the age-standardised incidence rate decreased by 28·0% (95% UI 25·4–30·5) globally, while the age-standardised death rate dropped at a faster rate of 43·2% (41·0–45·1), and age-standaradised DALY rate by 47·1% (45·3–49·0; table; figure 4). The downward trend in age-standardised incidence rates did, however, plateau at the global level and for some regions in the last 5 years of the study period. The decreases in age-standardised incidence and mortality from 1990 to 2017 were greater for females than for males at the global level and in many regions (appendix pp 31–32). The high-income Asia Pacific region had the sharpest drop in age-standardised rates between 1990 and 2017 compared with all other regions (decrease in age-standardised incidence by 48·7% [45·9–51·1]; age-standardised deaths by 56·7% [54·7–58·3]; and age-standardised DALYs by 62·4% [60·6–63·8]). In east Asia, the drop in age-standardised incidence rate was not as sharp (14·7% [8·9–21·5]); however, a 44·5% (41·2–48·7) decrease in age-standardised deaths caused the age-standardised DALY rate to reduce by nearly half (49·7%, 46·6–53·3; table).

**Figure 5:**
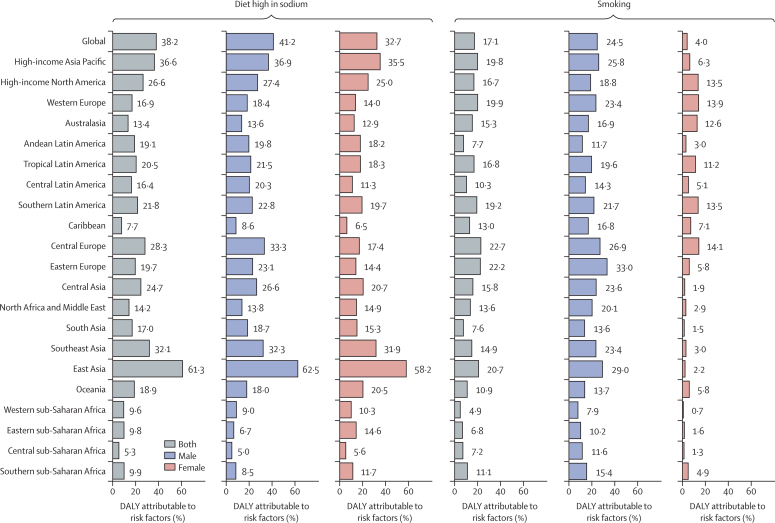
Percentage of stomach cancer age-standardised DALYs attributable to high-sodium diet and smoking in 2017, by sex, globally and for 21 GBD regions DALYs=disability-adjusted life-years. GBD=Global Burden of Diseases, Injuries, and Risk Factors Study.

The percentage of age-standardised DALYs attributable to high-sodium diet and smoking in each region are shown in figure 5. Globally, 38·2% (95% UI 21·1–57·8) of the age-standardised DALYs were attributable to a high-sodium diet, which was moderately higher in males than in females (41·2% [23·6–60·9] for males; and 32·7% [16·4–52·7] for females). In east Asia, this figure was almost twice that of all other regions, with 61·3% (42·8–78·2) of age-standardised DALYs attributable to a high-sodium diet. For males, 24·5% (20·0–28·9) of the age-standardised DALYs globally were attributable to smoking. Eastern Europe (33·0%, 27·5–37·9) and east Asia (29·0%, 23·5–34·2) had the highest percentage of age-standardised DALYs attributable to smoking among males. In females, smoking did not account for a sizeable fraction of age-standardised DALYs globally, but in parts of Europe (particularly western and central), high-income North America, Australasia, and parts of Latin America (particularly tropical and southern), the contribution of smoking to stomach cancer age-standardised DALYs among females was higher than 10%.

## Discussion

Stomach cancer is an important cause of morbidity and mortality in many parts or the world, and the total numbers of incident cases and deaths are increasing worldwide. East Asia, particularly China, contributes the largest number of incident cases, deaths, and DALYs from stomach cancer globally. However, the age-standardised incidence and death rates have declined steadily.

Our findings generally agree with those of the Global Cancer Incidence, Mortality and Prevalence (GLOBOCAN) project,^28^ although our estimates were somewhat higher than theirs, possibly as a result of differences in data sources and estimation methods. From 1990 to 2017, stomach cancer dropped from the fifth leading incident cancer worldwide to the seventh, and from the second leading cause of cancer deaths to third (after lung and colorectal cancers).^22^ As a result, stomach cancer accounts for the third highest cancer-related DALYs after lung and liver cancers. However, this decline in burden relative to other cancers and the dramatic decline in age-standardised rates have not necessarily led to a lower burden of stomach cancer on the health systems in high-risk countries. This is because changes in the population age structure and population growth have meant that numbers of incident cases and deaths of stomach cancer have continued to increase in many locations.^22^ Based on our findings, a further decrease in the absolute number of cases and deaths could be possible if the rates in east Asia, where almost half of the incident cases and deaths occur, are further reduced.

Based on migrant studies^29^ and the secular trends in stomach cancer rates, environmental factors are thought to play a significant role in the pathogenesis of stomach cancer.^3^ In contrast, only about 10% of cases aggregate in families, and only 1–3% occur as part of known hereditary syndromes.^1^ Our results suggest lifestyle factors play a significant role in stomach cancer burden, in particular high-sodium diet in east Asian populations and smoking among males. Both of these are also risk factors for other non-communicable diseases^30^ and minimal exposure to them is generally suggested in guidelines for a healthy lifestyle. Reducing high-salt foods in the diet is one of the ways proposed to tackle the stomach cancer problem in high-risk Asian countries.^31^

*H pylori* infection is the most important established risk factor for stomach cancer, and, as a result, most of the prevention strategies against this cancer focus on this infection. *H pylori* was once a ubiquitous infection,^32^ and in populations where infection rates are high, stomach cancer is a significant public health problem despite other interventions.^3^, ^33^ In some countries in western Europe (eg, Germany, the UK, and Spain) and in the USA, the previously declining burden of stomach cancer has plateaued, especially in the middle-aged (ie, 50–64 years) population, probably due to a low and stable prevalence of *H pylori* infection.^34^ For our study, we were unable to evaluate the role of *H pylori* infection in stomach cancer burden: such data are not included in registries and population-level data sources because they are costly and difficult to obtain at large scales. However, most of the risk reduction due to improved socio-economic status (even in the absence of specific preventive strategies) is thought to stem from reduced *H pylori* infection rates.^35^ Beyond general sanitation and improved socio-economic status, *H pylori* can also be effectively eradicated by different drug regimens. There is some controversy over whether population eradication of *H pylori* would be a cost-effective strategy to lower the burden of stomach cancer, and a trial to address this question would be logistically challenging and resource-intensive because of the period required for follow-up. In a systematic review and meta-analysis, Lee and colleagues^36^ reported that mass eradication of *H pylori* infection was associated with a reduced incidence of stomach cancer. They concluded that the benefit of eradication was dependent on the stomach cancer risk at baseline. In another meta-analysis,^37^ Ford and colleagues limited their study to randomised controlled trials and showed limited, moderate-quality evidence for reduced incidence of stomach cancer associated with screening for and eradicating *H pylori* in healthy asymptomatic infected individuals from Asia. This study concluded that these results might not necessarily be generalisable to other populations.

We found that reductions in age-standardised incidence rates did not necessarily parallel those of age-standardised death and DALY rates, meaning that while age-standardised death rates fell considerably in many locations, age-standardised incidence rates decreased more slowly. For example, east Asia, particularly China, witnessed a relatively small decrease in age-standardised incidence rates over the study period, whereas the decreases in age-standardised death and attributable DALY rates were much larger. A study of cancer registries in China showed that the disparity in cancer mortality rates was far greater than cancer incidence when rural and urban areas were compared.^38^ The authors suggested that this disparity was due to limited medical resources, lower levels of cancer care, and a larger proportion of patients diagnosed with cancer at a late stage in rural and underdeveloped areas. China has taken steps to reduce the cancer care disparities between rural and urban areas,^39^ and we think these efforts might explain the sharp decrease in death rates due to stomach cancer. Another successful strategy to reduce deaths due to stomach cancer has been used in Japan and South Korea, two of the high-income Asia Pacific countries. These countries have implemented population screening programmes leading to early detection of cancer cases^40^ and better survival rates. This has led some investigators to believe that China should adopt a similar strategy to further reduce the burden of stomach cancer.^41^ But population screening for stomach cancer includes invasive methods, and its feasibility and cost-effectiveness outside high-risk regions have never been investigated.^2^ A serum pepsinogen test combined with *H pylori* testing has been suggested as a potential method to triage suitable candidates for more invasive screening methods, but evidence for their clinical application is still mainly limited to Japanese populations.^42^Although regional patterns convey a lot of useful information about the distribution of stomach cancer burden and its trends and correlates, significant variations exist within each region. For example, stomach cancer incidence rates in Canada are almost double those in the USA, and stomach cancer contributes to more age-standardised DALYs per 100 000 population in Portugal, Chile, Guatemala, Bolivia, Haiti, Zimbabwe, and Mali than in other countries in the same regions. In addition, the two countries with the highest age-standardised incidence, death, and DALY rates globally (Mongolia and Afghanistan) have much higher rates than those of their respective regions (central Asia, and north Africa and Middle East, respectively). While making such comparisons, it is important to note that data quality differs across individual countries, and GBD uses a rating system (from zero to five stars) to assess the quality of the available data from each country.^43^ In addition to data quality, differences in data collection and coding systems are other challenges facing such comparisons.

A major limitation of our study was the inability to distinguish cardia and non-cardia forms of stomach cancer. Non-cardia stomach cancer is predominantly associated with *H pylori* infection and is probably the main reason for the changing rates of stomach cancer across the world.^4^ Cardia tumours are estimated to account for about 12% of stomach cancers globally, but are responsible for a higher proportion of stomach cancer burden in some low-risk populations.^44^ Cardia cancer in the USA is more common among the non-Hispanic white population and is not strongly linked to socio-economic status.^45^ Comparing different populations and studying the secular trends of cardia versus non-cardia tumours over time is complex, because the definition of cardia cancer has evolved over time, and some cardia tumours might have been classified as lower oesophageal adenocarcinomas, and vice versa.^8^ We could not determine the burden of stomach cancer directly attributable to *H pylori*, as described above, and the lack of data on some of the other risk factors limited our risk factor analysis. We also did not have information on the molecular subtypes of stomach tumours.^46^ As a general limitation of GBD, we relied on estimates from the modelling process for locations where data had low levels of completeness. But, by providing comprehensive measures of uncertainty, the degree of error in the estimates resulting from data scarcity is clarified.

Beyond general improvements in socio-economic status leading to improved health and lower *H pylori* infection rates, specific local strategies are needed to further reduce the number of incident cases and deaths due to stomach cancer, and these should be tailored to each country's risk factor profile. Targeting the risk factors that affect stomach cancer incidence and mortality (such as smoking and diet), in addition to country-specific feasible and cost-effective interventions aimed at lowering *H pylori* infection rates, early detection of suspected cases, and improved access to standard treatment facilities, can be among such strategies. By providing annual updates to regional and country-level stomach cancer estimates, future iterations of GBD will be useful for monitoring the success of such strategies.

**This online publication has been corrected. The corrected version first appeared at thelancet.com/gastrohep on Feb 12, 2020**
