# Correction to *Lancet Gastroenterol Hepatol* 2020; 5: 42–54

**DOI:** 10.1016/S2468-1253(20)30020-0

**Published:** 2020-02-12

*GBD 2017 Stomach Cancer Collaborators. The global, regional, and national burden of stomach cancer in 195 countries and territories, 1990–2017: a systematic analysis for the Global Burden of Disease Study 2017*. Lancet Gastroenterol Hepatol *2020; **5:** 42–54*—In this Article, the affiliation for C La Vecchia should read “Clinical Medicine and Community Health, University of Milan, Milano, Italy (Prof C La Vecchia MD)”. These changes have been made to the online version as of Feb 12, 2020.

