# Supplementary material for: The global, regional, and national burden of stomach cancer in 195 countries, 1990–2017: a systematic analysis for the Global Burden of Disease study 2017
Source: Lancet Gastroenterol Hepatol. 2019 Oct 21;5(1):42–54. doi: 10.1016/S2468-1253(19)30328-0 (PMC7033564; doi:10.1016/S2468-1253(19)30328-0)
Supplement: Supplementary appendix [file mmc1.pdf]

THE LANCET
Gastroenterology & Hepatology

Supplementary appendix

This appendix formed part of the original submission and has been peer reviewed. We post it as supplied by the authors.

Supplement to: GBD 2017 Stomach Cancer Collaborators. The global, regional, and national burden of stomach cancer in 195 countries, 1990–2017: a systematic analysis for the Global Burden of Disease study 2017. *Lancet Gastroenterol Hepatol* 2019; published online Oct 21. http://dx.doi.org/10.1016/S2468-1253(19)30328-0.

Supplementary appendix

Supplement to: **The global, regional, and national burden of stomach cancer and its attributable risk factors in 195 countries and territories, 1990–2017: a systematic analysis for the Global Burden of Disease Study 2017**

Contents:

Appendix Tables

Appendix Table 1. Incident cases of stomach cancer in 1990 and 2017 for both sexes and percentage change of age-standardised rates by country and region

Appendix Table 2. Deaths due to stomach cancer in 1990 and 2017 for both sexes and percentage change of age-standardised rates by country and region

Appendix Table 3. DALYs of stomach cancer in 1990 and 2017 for both sexes and percentage change of age-standardised rates by country and region

Appendix Figures

Appendix Figure 1. Age-standardised mortality rates of stomach cancer per 100,000 population in 2017, by country and territory

Appendix Figure 2. Age-standardised DALY rates of stomach cancer per 100,000 population in 2017, by country and territory

Appendix Figure 3. Global number of incident cases and incidence rates of stomach cancer per 100,000 population by age and sex, 2017; Dotted and dashed lines indicate 95% upper and lower uncertainty intervals, respectively.

Appendix Figure 4. Global number of deaths and death rates of stomach cancer per 100,000 population by age and sex, 2017; Dotted and dashed lines indicate 95% upper and lower uncertainty intervals, respectively.

Appendix Figure 5. Global number of DALYs and DALY rates of stomach cancer per 100,000 population by age and sex, 2017; Dotted and dashed lines indicate 95% upper and lower uncertainty intervals, respectively.

Appendix Figure 6. The percentage change in age-standardised incidence rates of stomach cancer from 1990 to 2017 for 21 Global Burden of Disease regions by sex.

Appendix Figure 7. The percentage change in age-standardised mortality rates of stomach cancer from 1990 to 2017 for 21 Global Burden of Disease regions by sex.

Appendix Figure 8. Age-standardised DALY rate of stomach cancer versus SDI by country and territory, 2017; black line represents the expected age-standardised DALY rate for stomach cancer based solely on SDI. SDI=Socio-demographic Index.

Appendix Figure 9. Percentage of stomach cancer DALYs attributable to high-sodium diet and smoking in 2017, by sex and age

Appendix Figure 10. Age-standardised (a) incidence, (b) death, and (c) DALY rates of stomach cancer globally and for 21 GBD regions by SDI, 1990-2017. For each region, points from left to right depict estimates from each year from 1990 to 2017. Lines are based on fitted regression models. DALY=disability-adjusted life-year. SDI=Socio-demographic Index. GBD=Global Burden of Diseases, Injuries, and Risk Factors Study.

Appendix Table 1. Incident cases of stomach cancer in 1990 and 2017 for both sexes and percentage change of age-standardised rates by country and region

	1990		2017		Percentage change in age-standardised rates between 1990 and 2017
	Counts (95% UI)	Rate (95% UI)	Counts (95% UI)	Rate (95% UI)	
**Global**	864372 (846682, 890421)	21·3 (20·9, 21·9)	1220662 (1189032, 1254563)	15·4 (15, 15·8)	-28 (-30·5, -25·4)
**High-income North America**	29797 (29287, 30385)	8·2 (8, 8·3)	39247 (37998, 40539)	6·5 (6·3, 6·7)	-20·4 (-23·3, -17·4)
**Canada**	4291 (4038, 4601)	12·9 (12·2, 13·8)	6999 (6186, 7825)	10·5 (9·3, 11·7)	-18·8 (-28·3, -8·4)
**Greenland**	7 (7, 8)	20 (18·5, 21·5)	8 (7, 8)	11·4 (10·4, 12·5)	-43 (-49·3, -36·1)
**USA**	25498 (25032, 25992)	7·7 (7·5, 7·8)	32239 (31247, 33230)	6 (5·8, 6·2)	-21·7 (-24·8, -18·7)
**Australasia**	2800 (2722, 2882)	11·7 (11·4, 12)	4263 (3845, 4692)	8·8 (7·9, 9·7)	-24·9 (-32·4, -17·1)
**Australia**	2323 (2256, 2394)	11·6 (11·3, 12)	3673 (3264, 4101)	9 (8, 10)	-23·1 (-31·8, -14)
**New Zealand**	477 (446, 510)	11·9 (11·2, 12·7)	589 (528, 657)	7·8 (7·1, 8·7)	-34·3 (-41·7, -26·4)
**High-income Asia-Pacific**	117390 (115459, 119276)	57·5 (56·5, 58·4)	131636 (125691, 138437)	29·5 (28·2, 31)	-48·7 (-51·1, -45·9)
**Brunei**	26 (24, 29)	23·6 (21·5, 25·9)	44 (39, 49)	13·6 (12·2, 15·3)	-42·3 (-49·7, -33·9)
**Japan**	100751 (98939, 102516)	58·8 (57·7, 59·8)	106507 (101418, 111555)	29·6 (28·3, 30·9)	-49·7 (-52, -47·2)
**Singapore**	445 (423, 469)	19·6 (18·7, 20·7)	685 (619, 748)	10·2 (9·2, 11·1)	-48 (-53·5, -42·7)
**South Korea**	16169 (15702, 16672)	49·2 (47·9, 50·7)	24401 (21766, 27240)	29·2 (26·1, 32·5)	-40·8 (-47·7, -33·5)
**Western Europe**	100144 (98442, 101993)	16·7 (16·5, 17)	95266 (90462, 100111)	10·5 (10, 11)	-37·4 (-40·6, -34·2)
**Andorra**	6 (6, 7)	11·2 (9·7, 13)	13 (11, 15)	9·2 (7·8, 10·7)	-18·5 (-33·8, -0·7)
**Austria**	2087 (1992, 2200)	17·3 (16·5, 18·1)	1835 (1643, 2033)	10·2 (9·1, 11·2)	-40·9 (-47·5, -33·7)
**Belgium**	2071 (1966, 2198)	13·1 (12·4, 13·8)	1780 (1580, 2009)	7·7 (6·9, 8·7)	-40·7 (-47·9, -33)
**Cyprus**	68 (62, 75)	8 (7·3, 8·8)	161 (139, 185)	8·5 (7·4, 9·8)	6 (-9·7, 24)
**Denmark**	852 (825, 880)	10·4 (10·1, 10·8)	753 (686, 824)	6·8 (6·2, 7·5)	-34·8 (-40·6, -28·3)
**Finland**	1209 (1173, 1247)	16·8 (16·3, 17·3)	880 (808, 971)	7·5 (6·9, 8·2)	-55·3 (-59·2, -50·5)
**France**	9668 (9279, 10096)	11·2 (10·8, 11·7)	9682 (8647, 10935)	7·2 (6·4, 8)	-36 (-42·8, -28·2)
**Germany**	25461 (24346, 26652)	19·3 (18·5, 20·2)	22026 (19305, 24965)	11·8 (10·4, 13·3)	-38·8 (-46·5, -30·5)
**Greece**	1846 (1759, 1949)	12 (11·5, 12·6)	2379 (2150, 2669)	10·4 (9·4, 11·5)	-13·8 (-22·1, -2·8)
**Iceland**	51 (47, 56)	17·3 (15·9, 18·9)	47 (42, 52)	8·8 (7·9, 9·8)	-48·9 (-55·7, -42·1)
**Ireland**	560 (536, 586)	13·3 (12·8, 13·9)	711 (630, 803)	9·8 (8·7, 11)	-26·6 (-35·3, -16·6)
**Israel**	504 (483, 524)	10·3 (9·9, 10·7)	743 (679, 814)	6·6 (6·1, 7·3)	-35·6 (-41·3, -29·5)
**Italy**	19139 (18259, 20134)	20·9 (20, 22)	18639 (16544, 20965)	12·7 (11·3, 14·3)	-39·3 (-46·5, -31·6)
**Luxembourg**	76 (71, 81)	13·6 (12·8, 14·5)	86 (75, 100)	8·9 (7·7, 10·3)	-34·8 (-43·9, -24·1)

**Malta**	51<br>(48, 54)	11·8<br>(11·2, 12·4)	72<br>(65, 81)	8·2<br>(7·4, 9·1)	-30·6<br>(-37·7, -21·4)
**Netherlands**	3204<br>(3027, 3450)	15·6<br>(14·7, 16·7)	3801<br>(3371, 4280)	11·3<br>(10·1, 12·7)	-27·4<br>(-35·8, -17·6)
**Norway**	966<br>(943, 990)	13·8<br>(13·5, 14·2)	667<br>(626, 713)	7·1<br>(6·7, 7·6)	-48·7<br>(-51·9, -45)
**Portugal**	3521<br>(3421, 3632)	25·8<br>(25, 26·5)	3426<br>(3134, 3751)	15·7<br>(14·2, 17·2)	-39<br>(-45·1, -32·8)
**Spain**	10770<br>(10410, 11116)	19·3<br>(18·7, 19·9)	11881<br>(10506, 13294)	12·3<br>(11, 13·7)	-36·2<br>(-44, -28·7)
**Sweden**	1825<br>(1766, 1884)	11·8<br>(11·4, 12·1)	1100<br>(1022, 1182)	5·4<br>(5, 5·8)	-54·2<br>(-57·6, -50·3)
**Switzerland**	1340<br>(1255, 1450)	12·4<br>(11·7, 13·4)	1132<br>(999, 1289)	6·6<br>(5·9, 7·5)	-46·8<br>(-54, -38·9)
**United Kingdom**	14776<br>(14425, 15126)	15·5<br>(15·2, 15·9)	13351<br>(12969, 13769)	10·3<br>(10·1, 10·7)	-33·5<br>(-35·8, -30·8)
**Southern Latin America**	8770<br>(8580, 8970)	18·8<br>(18·4, 19·2)	10145<br>(9436, 10956)	12·4<br>(11·5, 13·4)	-34·1<br>(-39, -28·5)
**Argentina**	4910<br>(4743, 5079)	15<br>(14·5, 15·5)	5111<br>(4579, 5712)	9·6<br>(8·6, 10·7)	-36·2<br>(-43·4, -28·1)
**Chile**	3079<br>(2985, 3172)	30·6<br>(29·8, 31·6)	4313<br>(3864, 4824)	18·6<br>(16·7, 20·8)	-39·3<br>(-46, -31·9)
**Uruguay**	782<br>(753, 812)	19·7<br>(19·1, 20·5)	721<br>(646, 798)	13·4<br>(11·9, 14·8)	-32·3<br>(-39·8, -24·2)
**Eastern Europe**	84567<br>(82245, 87075)	29·3<br>(28·5, 30·1)	59809<br>(57983, 61663)	17·7<br>(17·2, 18·3)	-39·5<br>(-41·4, -37·5)
**Belarus**	4224<br>(4093, 4361)	32<br>(31, 33)	2885<br>(2612, 3186)	18·6<br>(16·8, 20·5)	-42<br>(-47·8, -35·7)
**Estonia**	570<br>(549, 592)	27·4<br>(26·4, 28·4)	465<br>(400, 538)	18·7<br>(16·2, 21·8)	-31·8<br>(-41·3, -20·8)
**Latvia**	931<br>(899, 966)	25·4<br>(24·5, 26·3)	674<br>(587, 772)	17·2<br>(15·1, 19·6)	-32·2<br>(-40·6, -22·9)
**Lithuania**	1179<br>(1138, 1224)	25·5<br>(24·6, 26·5)	973<br>(885, 1077)	17·4<br>(15·9, 19·1)	-31·8<br>(-38, -24·6)
**Moldova**	844<br>(816, 874)	18·1<br>(17·5, 18·7)	556<br>(522, 591)	9·9<br>(9·3, 10·5)	-45·4<br>(-49·2, -41·7)
**Russia**	56123<br>(54321, 58031)	30<br>(29·1, 31)	40871<br>(39789, 42060)	17·8<br>(17·4, 18·3)	-40·6<br>(-42·6, -38·6)
**Ukraine**	20695<br>(19666, 21809)	28·4<br>(27, 29·8)	13385<br>(12408, 14482)	17·8<br>(16·6, 19·3)	-37·2<br>(-42·5, -31·3)
**Central Europe**	24952<br>(24408, 25423)	16·5<br>(16·2, 16·8)	19794<br>(19194, 20462)	9·4<br>(9·1, 9·7)	-43·3<br>(-45·4, -41·2)
**Albania**	305<br>(289, 322)	13·6<br>(12·9, 14·5)	435<br>(361, 522)	10·5<br>(8·7, 12·6)	-23·2<br>(-37, -6·2)
**Bosnia and Herzegovina**	513<br>(489, 540)	12·2<br>(11·7, 12·8)	553<br>(507, 599)	9·3<br>(8·6, 10·1)	-23·6<br>(-31, -15·9)
**Bulgaria**	2162<br>(2073, 2249)	16·8<br>(16·2, 17·5)	1384<br>(1281, 1491)	9·5<br>(8·8, 10·3)	-43·4<br>(-48·1, -38·4)
**Croatia**	1475<br>(1406, 1550)	22·7<br>(21·7, 23·9)	1107<br>(1006, 1227)	12·5<br>(11·4, 13·8)	-45<br>(-50·4, -38·6)
**Czech Republic**	2482<br>(2398, 2568)	17·8<br>(17·2, 18·4)	1765<br>(1591, 1960)	8·6<br>(7·7, 9·5)	-51·7<br>(-56·6, -46·5)
**Hungary**	2844<br>(2733, 2959)	18·9<br>(18·2, 19·7)	1615<br>(1507, 1731)	8·4<br>(7·9, 9)	-55·5<br>(-59·3, -51·4)
**Macedonia**	354<br>(330, 378)	18·5<br>(17·2, 19·8)	381<br>(348, 419)	11·3<br>(10·3, 12·3)	-39<br>(-45·7, -31·7)
**Montenegro**	46<br>(41, 54)	7·4<br>(6·5, 8·5)	62<br>(55, 69)	6·2<br>(5·5, 6·9)	-16·2<br>(-31·1, 0·6)
**Poland**	7569<br>(7385, 7752)	16·7<br>(16·3, 17·1)	5923<br>(5535, 6308)	8·7<br>(8·1, 9·2)	-48·1<br>(-51·5, -44·4)
**Romania**	3974<br>(3805, 4132)	13·8<br>(13·2, 14·3)	3453<br>(3245, 3686)	9·5<br>(9, 10·2)	-31<br>(-36·1, -25·4)

**Serbia**	1433 (1273, 1622)	12·4 (11·1, 13·9)	1360 (1252, 1476)	8·7 (8, 9·5)	-29·7 (-39·4, -19·9)
**Slovakia**	1284 (1230, 1336)	21·2 (20·3, 22)	1254 (1107, 1416)	13·9 (12·3, 15·6)	-34·4 (-42·4, -25·1)
**Slovenia**	512 (488, 539)	20·6 (19·6, 21·6)	504 (453, 565)	11·8 (10·6, 13·2)	-42·7 (-49·2, -35·4)
**Central Asia**	11320 (11074, 11570)	23·1 (22·6, 23·6)	10513 (10059, 10965)	14·1 (13·5, 14·7)	-38·9 (-41·6, -36·2)
**Armenia**	569 (540, 600)	20 (19·1, 21)	518 (491, 545)	12·5 (11·8, 13·1)	-37·5 (-41·9, -32·7)
**Azerbaijan**	1217 (1153, 1282)	23 (21·9, 24·2)	1749 (1538, 1964)	19·5 (17·2, 21·7)	-15·4 (-25·9, -4·4)
**Georgia**	937 (887, 988)	14·8 (14, 15·6)	827 (782, 875)	14·2 (13·4, 15)	-4 (-11·1, 3·8)
**Kazakhstan**	3900 (3764, 4027)	29·1 (28·1, 30)	2237 (2076, 2410)	13 (12·1, 14)	-55·2 (-58·2, -51·9)
**Kyrgyzstan**	885 (838, 936)	28·3 (26·8, 29·8)	626 (588, 666)	14 (13·2, 14·9)	-50·5 (-53·9, -46·5)
**Mongolia**	623 (574, 677)	59·5 (55, 64·4)	764 (685, 852)	35·6 (31·9, 39·6)	-40·1 (-47·2, -32·5)
**Tajikistan**	680 (652, 708)	23·2 (22·3, 24·2)	928 (832, 1026)	17·4 (15·6, 19·2)	-25·3 (-33·4, -16·9)
**Turkmenistan**	386 (372, 401)	19·1 (18·5, 19·8)	375 (346, 408)	9·7 (9, 10·5)	-49·5 (-53·5, -44·8)
**Uzbekistan**	2123 (2048, 2203)	17·7 (17·1, 18·4)	2491 (2224, 2807)	10·8 (9·7, 12·1)	-39 (-45·8, -30·9)
**Central Latin America**	13043 (12833, 13342)	15·6 (15·3, 15·9)	29601 (28255, 31008)	12·9 (12·3, 13·5)	-16·9 (-20·8, -12·7)
**Colombia**	4128 (4003, 4300)	23·7 (23, 24·5)	8559 (7418, 9825)	15·9 (13·8, 18·2)	-32·9 (-41·5, -23·1)
**Costa Rica**	673 (636, 715)	38·5 (36·3, 41)	1501 (1354, 1654)	30·9 (27·8, 34)	-19·9 (-29·4, -10·4)
**El Salvador**	396 (373, 422)	13·2 (12·4, 14·1)	956 (807, 1129)	16·7 (14·1, 19·7)	26·7 (6·5, 51)
**Guatemala**	743 (719, 770)	21 (20·3, 21·7)	2076 (1873, 2280)	19·4 (17·5, 21·3)	-7·6 (-17, 2·3)
**Honduras**	176 (156, 198)	8 (7·1, 9)	414 (338, 501)	7·1 (5·8, 8·5)	-11·7 (-28, 10·3)
**Mexico**	4786 (4707, 4876)	11·1 (10·9, 11·3)	11300 (10968, 11639)	10·1 (9·8, 10·4)	-9·4 (-12·5, -6·2)
**Nicaragua**	206 (189, 224)	12·5 (11·5, 13·6)	429 (378, 488)	9·4 (8·3, 10·8)	-24·4 (-34·6, -12·5)
**Panama**	216 (206, 226)	14·2 (13·5, 14·9)	594 (537, 654)	15 (13·5, 16·5)	5·8 (-5·6, 17·2)
**Venezuela**	1721 (1662, 1785)	18 (17·4, 18·7)	3772 (3188, 4440)	14 (11·8, 16·4)	-22·4 (-34·8, -8·9)
**Andean Latin America**	5147 (4906, 5393)	24·8 (23·7, 26)	8925 (8194, 9658)	16·6 (15·2, 18)	-33·2 (-39·2, -26·6)
**Bolivia**	1352 (1193, 1548)	42 (37·4, 47·7)	2071 (1696, 2499)	24·9 (20·6, 30)	-40·8 (-52·4, -26·5)
**Ecuador**	1440 (1405, 1479)	26·7 (26·1, 27·5)	2512 (2287, 2762)	17·3 (15·7, 19)	-35·4 (-41·4, -28·9)
**Peru**	2356 (2178, 2521)	19·4 (18, 20·8)	4343 (3782, 4921)	14·1 (12·3, 16)	-27·2 (-37·7, -14·8)
**Caribbean**	2798 (2661, 2958)	10·6 (10·1, 11·2)	4033 (3769, 4342)	7·9 (7·4, 8·5)	-25·2 (-30·4, -19·6)
**Antigua and Barbuda**	7 (7, 8)	13·8 (12·9, 14·7)	9 (8, 10)	9·2 (8·5, 10)	-33 (-39·4, -25·7)
**The Bahamas**	20 (18, 21)	12·2 (11·6, 12·9)	31 (28, 34)	8·3 (7·6, 9·2)	-31·7 (-38·7, -23·3)
**Barbados**	45 (42, 47)	14·5 (13·8, 15·2)	43 (39, 48)	8·9 (8·1, 10)	-38·5 (-44·8, -31)

**Belize**	11<br>(10, 12)	11·2<br>(10·4, 12·2)	22<br>(21, 23)	8·4<br>(7·9, 8·9)	-25·6<br>(-32·8, -17·9)
**Bermuda**	7<br>(6, 7)	11<br>(10·3, 11·8)	10<br>(9, 11)	7·7<br>(6·7, 8·8)	-29·9<br>(-39·1, -19·5)
**Cuba**	769<br>(744, 797)	7·4<br>(7·1, 7·6)	1174<br>(1038, 1327)	6·3<br>(5·5, 7·1)	-15<br>(-25·3, -3·5)
**Dominica**	15<br>(14, 16)	20·6<br>(19·5, 21·7)	14<br>(13, 16)	15·5<br>(14·4, 16·8)	-24·6<br>(-31·1, -17·5)
**Dominican Republic**	264<br>(243, 286)	6·8<br>(6·3, 7·4)	660<br>(568, 754)	7·1<br>(6·1, 8·2)	4·2<br>(-11·7, 22)
**Grenada**	9<br>(9, 10)	13·3<br>(12·6, 14·1)	13<br>(12, 14)	8·2<br>(7·6, 8·7)	-38·7<br>(-43·8, -33)
**Guyana**	39<br>(38, 41)	10·1<br>(9·6, 10·6)	40<br>(35, 44)	6·5<br>(5·8, 7·3)	-35<br>(-42·4, -26·5)
**Haiti**	743<br>(616, 891)	23·1<br>(19·3, 27·7)	979<br>(782, 1198)	15·1<br>(12·3, 18·5)	-34·4<br>(-46·3, -20·5)
**Jamaica**	238<br>(224, 253)	13<br>(12·2, 13·8)	268<br>(231, 311)	9·2<br>(8, 10·7)	-28·8<br>(-39·5, -16·5)
**Puerto Rico**	393<br>(377, 411)	10·5<br>(10·1, 11)	460<br>(409, 519)	6·4<br>(5·7, 7·1)	-39·5<br>(-45·6, -32)
**Saint Lucia**	15<br>(14, 16)	16·9<br>(16·1, 17·8)	21<br>(20, 23)	10·2<br>(9·5, 11)	-39·9<br>(-44·9, -34·6)
**Saint Vincent and the Grenadines**	10<br>(9, 10)	13·1<br>(12·4, 13·8)	13<br>(12, 14)	9·4<br>(8·7, 10·1)	-28·4<br>(-34·9, -21·7)
**Suriname**	22<br>(21, 24)	8·8<br>(8·1, 9·4)	35<br>(31, 38)	6·1<br>(5·4, 6·7)	-30·6<br>(-39·2, -21·3)
**Trinidad and Tobago**	85<br>(81, 90)	10·1<br>(9·6, 10·6)	79<br>(66, 94)	4·5<br>(3·8, 5·4)	-55·2<br>(-63, -46·4)
**Virgin Islands**	9<br>(8, 10)	10·8<br>(10, 11·6)	19<br>(15, 21)	10<br>(8·3, 11·4)	-7·5<br>(-24·6, 8)
**Tropical Latin America**	14375<br>(14105, 14644)	15·8<br>(15·5, 16·1)	21823<br>(21369, 22331)	9·5<br>(9·3, 9·7)	-40·2<br>(-41·8, -38·5)
**Brazil**	14145<br>(13870, 14412)	15·9<br>(15·6, 16·2)	21399<br>(20946, 21887)	9·5<br>(9·3, 9·7)	-40·5<br>(-42, -38·8)
**Paraguay**	230<br>(213, 248)	10·5<br>(9·7, 11·3)	423<br>(358, 505)	8·1<br>(6·9, 9·7)	-22·8<br>(-36·5, -6·1)
**East Asia**	307683<br>(296319, 325735)	33·5<br>(32·3, 35·5)	583758<br>(554933, 612688)	28·6<br>(27·3, 30)	-14·7<br>(-21·5, -8·9)
**China**	295862<br>(284788, 312862)	34·1<br>(32·8, 36·1)	561938<br>(533157, 590626)	29<br>(27·6, 30·4)	-14·9<br>(-21·9, -8·9)
**North Korea**	3536<br>(2913, 4224)	20·8<br>(17·3, 24·5)	6407<br>(5181, 7787)	20·4<br>(16·6, 24·6)	-2·2<br>(-24·5, 25·6)
**Taiwan (Province of China)**	3160<br>(3068, 3255)	19·1<br>(18·6, 19·6)	6009<br>(5445, 6617)	15·8<br>(14·4, 17·4)	-17<br>(-25, -8·3)
**Southeast Asia**	31755<br>(29156, 34304)	12·1<br>(11·2, 13·1)	39191<br>(36559, 42265)	6·8<br>(6·4, 7·3)	-43·8<br>(-49, -38·2)
**Cambodia**	902<br>(739, 1088)	19·4<br>(15·9, 23·2)	1047<br>(894, 1247)	9·6<br>(8·3, 11·3)	-50·6<br>(-58·9, -40·5)
**Indonesia**	10514<br>(9314, 11735)	10·6<br>(9·3, 11·9)	14835<br>(13416, 16570)	7·5<br>(6·8, 8·3)	-29·4<br>(-37·8, -20·4)
**Laos**	396<br>(319, 481)	18·5<br>(15, 22·4)	348<br>(285, 421)	8·5<br>(7·1, 10·2)	-53·9<br>(-63·4, -42·4)
**Malaysia**	957<br>(872, 1046)	11<br>(10, 12)	1680<br>(1476, 1886)	7·1<br>(6·3, 8)	-35·3<br>(-46·2, -24·2)
**Maldives**	7<br>(6, 8)	7·5<br>(6·4, 8·7)	7<br>(6, 8)	2·4<br>(2·2, 2·7)	-67·6<br>(-73·6, -60·3)
**Mauritius**	94<br>(89, 99)	12·8<br>(12·2, 13·5)	110<br>(101, 120)	6·8<br>(6·3, 7·4)	-47·1<br>(-51·9, -41·7)
**Myanmar**	5326<br>(4337, 6543)	22·2<br>(18·2, 27·1)	4366<br>(3712, 5215)	10<br>(8·5, 12)	-54·9<br>(-64·2, -45)
**Philippines**	1947<br>(1835, 2068)	6·6<br>(6·2, 6·9)	2734<br>(2382, 3115)	4<br>(3·5, 4·5)	-39·5<br>(-47·5, -29·7)

**Sri Lanka**	834<br>(770, 900)	8<br>(7·3, 8·6)	1016<br>(841, 1207)	4·3<br>(3·6, 5)	-46·3<br>(-56·2, -35·1)
**Seychelles**	6<br>(6, 7)	11<br>(10, 12)	6<br>(6, 7)	5·7<br>(5·2, 6·2)	-48·2<br>(-54·5, -41·4)
**Thailand**	3190<br>(2938, 3440)	8·7<br>(8, 9·4)	4625<br>(4113, 5239)	4·8<br>(4·3, 5·4)	-44·7<br>(-52·4, -35·9)
**East Timor**	37<br>(30, 45)	12·8<br>(10·5, 15·4)	62<br>(50, 77)	8·1<br>(6·5, 9·9)	-36·3<br>(-49·9, -20·1)
**Vietnam**	7502<br>(6636, 8424)	17·8<br>(15·8, 20)	8302<br>(7182, 9588)	9<br>(7·8, 10·3)	-49·6<br>(-58·6, -39·2)
**Oceania**	523<br>(439, 611)	16·2<br>(13·9, 18·6)	988<br>(815, 1177)	13·8<br>(11·7, 16·1)	-14·5<br>(-25·6, -3)
**American Samoa**	4<br>(3, 4)	15·6<br>(14·1, 17·1)	5<br>(4, 6)	12·6<br>(11·2, 13·9)	-19·5<br>(-30·8, -7·2)
**Federated States of Micronesia**	7<br>(6, 9)	15<br>(12·8, 17·5)	7<br>(6, 9)	11·4<br>(9·5, 13·4)	-24·4<br>(-38·3, -7·7)
**Fiji**	22<br>(20, 25)	6·3<br>(5·6, 7)	37<br>(32, 42)	5·5<br>(4·8, 6·2)	-13·1<br>(-26·4, 3·4)
**Guam**	4<br>(4, 5)	5·9<br>(5·4, 6·3)	9<br>(8, 10)	5·3<br>(4·8, 5·8)	-10<br>(-20·1, 2)
**Kiribati**	6<br>(5, 6)	14·8<br>(13·5, 16·3)	8<br>(6, 9)	11·3<br>(9·8, 12·9)	-23·6<br>(-35, -10·2)
**Marshall Islands**	3<br>(3, 4)	18·2<br>(16·2, 20·5)	5<br>(4, 6)	15·1<br>(12·8, 17·7)	-17·5<br>(-29·1, -4·1)
**Northern Mariana Islands**	2<br>(2, 2)	10·1<br>(8·9, 11·7)	3<br>(3, 4)	7·3<br>(6·5, 8·2)	-27·3<br>(-39·9, -12·8)
**Papua New Guinea**	387<br>(313, 466)	18·9<br>(15·5, 22·4)	774<br>(618, 947)	16<br>(12·9, 19)	-15·6<br>(-28·9, -1·2)
**Samoa**	10<br>(8, 11)	11·5<br>(9·8, 13·4)	13<br>(11, 15)	10·1<br>(8·7, 11·6)	-12·3<br>(-26·8, 4·9)
**Solomon Islands**	24<br>(20, 29)	16·5<br>(13·8, 19·7)	41<br>(34, 50)	12·5<br>(10·5, 14·9)	-24·3<br>(-36·6, -9·3)
**Tonga**	9<br>(8, 10)	17·2<br>(15·9, 18·6)	11<br>(9, 12)	13·6<br>(12, 15·4)	-21·1<br>(-32·4, -7)
**Vanuatu**	11<br>(8, 14)	15·6<br>(12·3, 19·5)	21<br>(16, 27)	13·1<br>(10·2, 16·6)	-16<br>(-33·1, 6·3)
**North Africa and Middle East**	23680<br>(21451, 25723)	13·5<br>(12·3, 14·6)	35755<br>(33988, 37539)	8·7<br>(8·3, 9·1)	-35·6<br>(-40·7, -29·7)
**Afghanistan**	2561<br>(1472, 3403)	36·4<br>(21·3, 48·2)	3794<br>(2994, 4765)	32·8<br>(26·5, 39·6)	-9·8<br>(-28·9, 45·8)
**Algeria**	891<br>(799, 981)	7·1<br>(6·4, 7·8)	1401<br>(1273, 1532)	4·3<br>(3·9, 4·7)	-39·2<br>(-45·9, -30·9)
**Bahrain**	18<br>(17, 19)	10·3<br>(9·5, 11·1)	33<br>(29, 36)	3·8<br>(3·4, 4·3)	-62·6<br>(-67·3, -57·3)
**Egypt**	1215<br>(1104, 1339)	4·6<br>(4·2, 5·1)	1895<br>(1679, 2134)	3·5<br>(3·1, 3·9)	-23·6<br>(-33·6, -11·4)
**Iran**	5300<br>(4876, 5732)	20·5<br>(18·9, 22·1)	9825<br>(9488, 10246)	14·6<br>(14·1, 15·2)	-28·7<br>(-34, -22·3)
**Iraq**	578<br>(490, 677)	7·1<br>(6·1, 8·3)	672<br>(623, 728)	2·8<br>(2·6, 3)	-60·9<br>(-67·3, -53·2)
**Jordan**	107<br>(92, 124)	7·3<br>(6·3, 8·3)	228<br>(202, 258)	4·2<br>(3·7, 4·7)	-42·9<br>(-53·3, -31)
**Kuwait**	30<br>(28, 32)	4·3<br>(4, 4·6)	59<br>(53, 65)	2·3<br>(2, 2·5)	-47<br>(-52·9, -40·1)
**Lebanon**	260<br>(222, 298)	11·6<br>(10, 13·2)	469<br>(419, 528)	7·7<br>(6·9, 8·7)	-33·4<br>(-44·3, -18·9)
**Libya**	133<br>(114, 155)	6·9<br>(5·9, 8)	298<br>(255, 346)	6·4<br>(5·5, 7·4)	-6·6<br>(-25·5, 16·6)
**Morocco**	736<br>(667, 814)	5·2<br>(4·7, 5·7)	1143<br>(958, 1351)	3·8<br>(3·2, 4·5)	-26·8<br>(-40·7, -11·3)
**Palestine**	83<br>(69, 99)	8·9<br>(7·4, 10·6)	128<br>(118, 140)	5·2<br>(4·8, 5·6)	-42·1<br>(-52·7, -29·3)

**Oman**	95 (78, 114)	13·2 (10·9, 15·8)	139 (114, 165)	7 (5·9, 8·2)	-46·7 (-57·4, -32·2)
**Qatar**	11 (9, 12)	10·1 (8·8, 11·7)	48 (39, 57)	5·6 (4·7, 6·7)	-44·7 (-55·7, -31·1)
**Saudi Arabia**	423 (350, 518)	7 (5·8, 8·6)	731 (638, 846)	4·8 (4·3, 5·5)	-31 (-45·6, -13·6)
**Sudan**	1689 (1289, 2085)	17·6 (13·7, 21·5)	2492 (1909, 3137)	13·9 (10·8, 17·4)	-20·8 (-37·9, 1·9)
**Syria**	314 (278, 350)	6 (5·3, 6·6)	501 (427, 586)	4·1 (3·5, 4·7)	-31·3 (-43·7, -16·3)
**Tunisia**	333 (302, 372)	6·9 (6·3, 7·6)	591 (480, 712)	5 (4·1, 6)	-27·3 (-42, -9·7)
**Turkey**	7727 (6957, 8491)	20·5 (18·4, 22·6)	8629 (7794, 9497)	9·9 (8·9, 10·9)	-51·8 (-58, -44·7)
**United Arab Emirates**	37 (30, 45)	9·3 (7·5, 11·4)	266 (206, 332)	8·5 (6·8, 10·3)	-9·2 (-31·7, 23·2)
**Yemen**	1127 (739, 1530)	21·3 (14·5, 28·3)	2381 (1781, 3042)	18·5 (14, 23·2)	-13 (-35·4, 27·9)
**South Asia**	64363 (59932, 69062)	10·3 (9·6, 11·1)	96577 (91436, 101062)	7·2 (6·8, 7·5)	-30·9 (-37·2, -25)
**Bangladesh**	6115 (5229, 7326)	12·4 (10·7, 14·7)	7490 (6339, 8802)	6·2 (5·2, 7·2)	-50·1 (-61·7, -38·2)
**Bhutan**	27 (22, 33)	10 (8·3, 12·3)	35 (28, 42)	5·7 (4·6, 7)	-43·2 (-57·1, -28)
**India**	53885 (49744, 57873)	10·7 (9·8, 11·6)	81672 (77287, 85894)	7·5 (7, 7·8)	-30·5 (-36·9, -23·9)
**Nepal**	1125 (932, 1366)	11·2 (9·3, 13·5)	1624 (1361, 1932)	7·6 (6·4, 9·1)	-32·1 (-45·4, -16·7)
**Pakistan**	3212 (2881, 3587)	5·5 (5, 6·2)	5756 (4763, 6842)	5·2 (4·4, 6·1)	-5·7 (-22·7, 13·7)
**Southern sub-Saharan Africa**	2055 (1947, 2177)	7·1 (6·8, 7·6)	2839 (2706, 2979)	5·2 (5, 5·4)	-27·3 (-32·3, -22·2)
**Botswana**	43 (37, 51)	7·7 (6·6, 8·9)	57 (50, 65)	4·5 (4, 5·2)	-41·2 (-51, -29·1)
**Lesotho**	100 (87, 115)	10·3 (8·9, 11·7)	112 (92, 132)	9·6 (7·9, 11·2)	-7·3 (-24·1, 12·9)
**Namibia**	38 (34, 43)	5·5 (4·9, 6·1)	42 (36, 49)	3·1 (2·7, 3·6)	-43·9 (-52·9, -33·4)
**South Africa**	1415 (1333, 1502)	6·4 (6, 6·8)	1880 (1806, 1962)	4·4 (4·2, 4·5)	-31·7 (-35·7, -27·2)
**Swaziland**	28 (24, 33)	9·7 (8·4, 11·2)	40 (33, 48)	7·3 (6·1, 8·6)	-25·4 (-40·2, -7·7)
**Zimbabwe**	428 (377, 479)	10·4 (9·1, 11·6)	708 (608, 816)	10·5 (9·1, 11·9)	0·9 (-17, 21·5)
**Western sub-Saharan Africa**	8393 (7594, 9250)	9·7 (8·8, 10·7)	12890 (11719, 14193)	7·7 (7·1, 8·5)	-20·5 (-27·9, -11·3)
**Benin**	289 (255, 325)	14·5 (12·8, 16·2)	497 (414, 589)	11·5 (9·7, 13·5)	-20·7 (-34·5, -4)
**Burkina Faso**	664 (537, 773)	15·7 (12·8, 18·1)	1055 (903, 1218)	12·8 (11, 14·8)	-18·2 (-32·4, 4·4)
**Cameroon**	626 (540, 704)	14·6 (12·6, 16·4)	1210 (987, 1452)	11·6 (9·6, 13·8)	-20·8 (-35·4, -4·6)
**Cape Verde**	56 (52, 61)	24·4 (22·3, 26·4)	69 (63, 76)	15·6 (14·2, 17·2)	-36·1 (-43·5, -27·9)
**Chad**	369 (320, 423)	13·1 (11·4, 15)	677 (565, 799)	13·5 (11·3, 15·8)	3 (-13·6, 24·6)
**Côte d'Ivoire**	258 (224, 294)	6·7 (5·9, 7·6)	507 (431, 599)	5·5 (4·7, 6·4)	-19·1 (-33, -2·9)
**The Gambia**	24 (20, 29)	7·2 (6·1, 8·5)	49 (42, 57)	5·7 (4·9, 6·5)	-21·9 (-35·4, -5)
**Ghana**	658 (560, 758)	11·1 (9·5, 12·7)	1069 (928, 1220)	7·7 (6·7, 8·7)	-30·7 (-42·5, -17)

**Guinea**	484 (430, 544)	14·2 (12·7, 16)	782 (666, 908)	14·5 (12·5, 16·8)	1·9 (-16·5, 22·2)
**Guinea-Bissau**	87 (73, 103)	21·7 (18·3, 25·6)	99 (84, 117)	15·2 (13, 17·6)	-30·2 (-41·9, -15·1)
**Liberia**	142 (123, 166)	12·8 (11·1, 14·7)	189 (160, 222)	10·6 (9·1, 12·4)	-16·6 (-30·8, 0·7)
**Mali**	953 (848, 1070)	22·9 (20·4, 25·7)	1201 (1002, 1415)	14·6 (12·3, 17·2)	-36·3 (-46·4, -23·5)
**Mauritania**	150 (132, 170)	14·7 (13, 16·7)	178 (147, 212)	9·6 (8, 11·5)	-34·6 (-45·8, -21·4)
**Niger**	403 (322, 481)	14·6 (11·9, 17·4)	786 (636, 943)	11·9 (9·8, 14·2)	-18·5 (-32·3, 0·5)
**Nigeria**	2337 (1776, 3003)	5·6 (4·3, 7)	2957 (2168, 4096)	4 (3, 5·4)	-28 (-47·4, -0·3)
**São Tomé and Príncipe**	10 (9, 11)	14·4 (13, 16·2)	15 (12, 17)	15·5 (12·8, 18·4)	7·4 (-13·7, 31·1)
**Senegal**	458 (402, 522)	14·5 (12·7, 16·5)	802 (686, 933)	12 (10·2, 13·8)	-17·5 (-30·4, -3)
**Sierra Leone**	261 (207, 313)	13·5 (10·8, 16·1)	390 (331, 455)	12·2 (10·4, 14·1)	-9·4 (-26·6, 18·3)
**Togo**	163 (143, 184)	13·4 (11·8, 15·1)	356 (294, 423)	11·3 (9·4, 13·2)	-15·9 (-30·5, 1·7)
**Eastern sub-Saharan Africa**	8340 (7311, 9313)	10·6 (9·5, 11·8)	10056 (9361, 10804)	6·4 (5·9, 6·8)	-40·2 (-46·5, -32·8)
**Burundi**	297 (244, 367)	13·1 (11, 16)	305 (247, 371)	7·4 (6·1, 8·8)	-43·4 (-53·7, -32·8)
**Comoros**	22 (19, 26)	10·6 (9, 12·6)	28 (23, 33)	6·2 (5·2, 7·3)	-41·6 (-52·8, -28·7)
**Djibouti**	14 (11, 20)	9·2 (7·1, 12·2)	34 (24, 46)	6·1 (4·4, 8)	-33·9 (-51·5, -9)
**Eritrea**	163 (125, 197)	15·9 (13, 18·7)	220 (180, 263)	9·1 (7·6, 10·7)	-42·6 (-53·1, -27·4)
**Ethiopia**	3041 (2415, 3812)	14·2 (11·5, 17·5)	2222 (1952, 2623)	5·5 (4·8, 6·5)	-61·4 (-69·3, -52·3)
**Kenya**	841 (727, 965)	9·9 (8·6, 11·3)	1919 (1732, 2170)	9 (8·2, 10·2)	-8·8 (-17·2, -0·9)
**Madagascar**	499 (442, 564)	9·2 (8·2, 10·5)	715 (582, 855)	6·6 (5·4, 7·9)	-28·4 (-41·3, -13·9)
**Malawi**	198 (130, 241)	5·2 (3·6, 6·1)	232 (200, 266)	3·3 (2·8, 3·7)	-37 (-48·8, -8·7)
**Mozambique**	473 (407, 547)	8·2 (7·1, 9·4)	687 (577, 798)	6·7 (5·7, 7·7)	-18·5 (-33·6, 1·2)
**Rwanda**	390 (326, 452)	13 (10·8, 15)	308 (255, 365)	5·5 (4·6, 6·4)	-57·6 (-66·1, -48·4)
**Somalia**	316 (192, 443)	11·8 (8·1, 15·8)	562 (426, 730)	8·7 (6·7, 11)	-26·4 (-46·6, 8·9)
**South Sudan**	257 (172, 359)	10·6 (7·6, 14·3)	302 (227, 396)	8 (6·1, 10·2)	-25 (-46·2, 9·2)
**Tanzania**	966 (739, 1156)	8·7 (6·9, 10·3)	1378 (1174, 1597)	5·8 (4·9, 6·6)	-33·6 (-45·6, -15·7)
**Uganda**	501 (429, 585)	7·6 (6·5, 8·8)	692 (594, 793)	5·1 (4·4, 5·9)	-32·5 (-44·6, -17·5)
**Zambia**	357 (294, 433)	12 (10·1, 14·5)	446 (374, 531)	6·9 (5·8, 8·2)	-42·6 (-52·6, -30·6)
**Central sub-Saharan Africa**	2477 (2119, 2854)	10·8 (9·4, 12·4)	3555 (3106, 4009)	7·1 (6·3, 8)	-34·3 (-43·2, -24·4)
**Angola**	542 (421, 657)	13·4 (10·8, 16)	706 (586, 829)	6·7 (5·6, 7·9)	-49·5 (-59, -36·4)
**Central African Republic**	177 (141, 210)	14·8 (12·3, 17·3)	225 (176, 273)	10·4 (8·3, 12·3)	-30 (-42·7, -15·6)
**Congo**	145 (126, 166)	13 (11·5, 14·8)	181 (150, 215)	7·3 (6·2, 8·5)	-43·7 (-54·1, -31·5)

**DR Congo**	1529<br>(1264, 1810)	9·8<br>(8·2, 11·5)	2365<br>(1953, 2772)	7·1<br>(6, 8·3)	-27·4<br>(-40·8, -10·9)
**Equatorial Guinea**	30<br>(23, 37)	14·7<br>(11·8, 18·2)	20<br>(14, 28)	4·5<br>(3·3, 6·1)	-69·5<br>(-78·6, -56·6)
**Gabon**	54<br>(44, 64)	9·6<br>(7·8, 11·3)	58<br>(49, 67)	5·7<br>(4·9, 6·5)	-40·5<br>(-50·8, -27·4)

Appendix Table 2. Deaths due to stomach cancer in 1990 and 2017 for both sexes and percentage change of age-standardised rates by country and region

	1990		2017		Percentage change in age-standardised rates between 1990 and 2017
	Counts (95% UI)	Rate (95% UI)	Counts (95% UI)	Rate (95% UI)	
**Global**	769069 (752137, 794640)	19·3 (18·9, 20)	864989 (848254, 884655)	11 (10·8, 11·2)	-43·2 (-45·1, -41·4)
**High-income North America**	21948 (21718, 22213)	5·9 (5·9, 6)	22159 (21591, 22750)	3·6 (3·5, 3·7)	-40 (-41·7, -38·3)
**Canada**	3429 (3348, 3519)	10·3 (10·1, 10·6)	4401 (4110, 4698)	6·3 (5·9, 6·7)	-39·1 (-43·2, -34·4)
**Greenland**	7 (6, 8)	20·7 (19·1, 22·3)	8 (7, 8)	11·7 (10·7, 12·8)	-43·5 (-49·6, -36·5)
**USA**	18511 (18305, 18748)	5·5 (5·4, 5·6)	17751 (17297, 18237)	3·2 (3·1, 3·3)	-41·5 (-43·2, -39·6)
**Australasia**	2025 (1983, 2071)	8·4 (8·3, 8·6)	2233 (2052, 2429)	4·4 (4·1, 4·8)	-47·4 (-51·7, -42·8)
**Australia**	1628 (1590, 1668)	8·2 (8, 8·4)	1880 (1707, 2071)	4·4 (4, 4·9)	-45·9 (-51, -40·3)
**New Zealand**	397 (383, 412)	9·9 (9·5, 10·2)	353 (331, 379)	4·6 (4·3, 4·9)	-53·8 (-56·9, -50·1)
**High-income Asia-Pacific**	66062 (65266, 66836)	32·8 (32·4, 33·2)	68042 (65688, 71099)	14·2 (13·7, 14·8)	-56·7 (-58·3, -54·7)
**Brunei**	23 (21, 25)	22·8 (20·9, 24·8)	32 (29, 35)	10·9 (9·9, 12)	-52·2 (-57·8, -45·8)
**Japan**	50929 (50297, 51533)	29·9 (29·6, 30·3)	55885 (54141, 58225)	14·2 (13·8, 14·8)	-52·5 (-54·1, -50·5)
**Singapore**	382 (369, 395)	17·7 (17·1, 18·3)	336 (312, 360)	5·1 (4·7, 5·5)	-71·2 (-73·4, -68·9)
**South Korea**	14728 (14376, 15104)	47·1 (46, 48·3)	11790 (10778, 12821)	14·1 (12·9, 15·3)	-70·1 (-72·7, -67·2)
**Western Europe**	85510 (84636, 86423)	14·1 (13·9, 14·2)	62213 (59851, 64525)	6·4 (6·2, 6·7)	-54·3 (-56, -52·6)
**Andorra**	5 (5, 6)	9·4 (8·3, 10·7)	8 (7, 9)	5·8 (5, 6·5)	-38·5 (-48·6, -27·3)
**Austria**	1901 (1850, 1952)	15·3 (14·9, 15·7)	1095 (1022, 1166)	5·8 (5·4, 6·2)	-62·1 (-64·6, -59·3)
**Belgium**	1982 (1918, 2049)	12·3 (11·9, 12·7)	1304 (1213, 1406)	5·3 (4·9, 5·7)	-57·1 (-60·4, -53·5)
**Cyprus**	71 (64, 78)	8·3 (7·6, 9·1)	119 (106, 133)	6·2 (5·5, 6·9)	-25·4 (-35·3, -14·2)
**Denmark**	743 (723, 763)	8·8 (8·6, 9)	570 (530, 614)	4·9 (4·6, 5·3)	-44 (-48·1, -39·5)
**Finland**	939 (916, 966)	12·8 (12·5, 13·2)	581 (542, 626)	4·7 (4·3, 5)	-63·7 (-66·4, -60·8)
**France**	9697 (9439, 10001)	10·9 (10·6, 11·2)	7386 (6837, 7985)	5 (4·6, 5·4)	-54·3 (-57·8, -50·6)
**Germany**	19202 (18724, 19684)	14·4 (14·1, 14·8)	13519 (12254, 14963)	6·8 (6·2, 7·6)	-52·6 (-57·2, -47·3)
**Greece**	1727 (1670, 1785)	11·1 (10·8, 11·5)	1878 (1745, 2020)	7·5 (6·9, 8·1)	-32·9 (-38, -27·4)
**Iceland**	39 (37, 41)	13·2 (12·6, 13·9)	28 (26, 30)	5 (4·7, 5·3)	-62·1 (-65·2, -59·2)
**Ireland**	554 (535, 574)	13·1 (12·7, 13·6)	463 (427, 503)	6·2 (5·8, 6·8)	-52·4 (-56·4, -48·2)
**Israel**	521 (501, 542)	10·7 (10·2, 11·1)	663 (615, 721)	5·7 (5·3, 6·2)	-46·1 (-50·2, -41·8)
**Italy**	17857 (17421, 18279)	19·3 (18·8, 19·7)	12117 (11267, 12996)	7·7 (7·2, 8·3)	-60 (-62·9, -57·1)
**Luxembourg**	69 (65, 72)	12·2 (11·7, 12·8)	54 (48, 60)	5·4 (4·7, 5·9)	-56·2 (-61·3, -50·8)

**Malta**	50<br>(48, 53)	11·8<br>(11·2, 12·4)	56<br>(52, 60)	6·2<br>(5·7, 6·6)	-47·8<br>(-51·7, -43·3)
**Netherlands**	2765<br>(2698, 2845)	13·3<br>(12·9, 13·6)	2422<br>(2268, 2582)	6·9<br>(6·5, 7·3)	-48<br>(-51·6, -44·4)
**Norway**	793<br>(781, 807)	11·1<br>(10·9, 11·3)	444<br>(428, 464)	4·5<br>(4·3, 4·7)	-59·3<br>(-60·9, -57·6)
**Portugal**	3611<br>(3508, 3721)	26·3<br>(25·6, 27)	2829<br>(2632, 3019)	11·5<br>(10·7, 12·3)	-56<br>(-59·6, -52·6)
**Spain**	8231<br>(8012, 8440)	14·6<br>(14·2, 14·9)	7009<br>(6530, 7505)	6·8<br>(6·3, 7·3)	-53·4<br>(-56·7, -49·8)
**Sweden**	1441<br>(1403, 1482)	8·9<br>(8·7, 9·2)	839<br>(793, 886)	3·8<br>(3·6, 4)	-57·6<br>(-60·1, -55)
**Switzerland**	1003<br>(972, 1033)	9·1<br>(8·8, 9·3)	688<br>(636, 744)	3·8<br>(3·5, 4·1)	-58·1<br>(-61·6, -54·4)
**United Kingdom**	12227<br>(12099, 12372)	12·6<br>(12·5, 12·8)	8077<br>(7931, 8256)	6<br>(5·9, 6·1)	-52·6<br>(-53·6, -51·4)
**Southern Latin America**	9220<br>(9015, 9437)	19·9<br>(19·5, 20·3)	10203<br>(9515, 10988)	12·3<br>(11·5, 13·2)	-38·3<br>(-42·8, -33·1)
**Argentina**	5163<br>(4982, 5348)	15·9<br>(15·4, 16·5)	5295<br>(4750, 5901)	9·8<br>(8·8, 11)	-38·3<br>(-45, -30·6)
**Chile**	3223<br>(3130, 3322)	32·7<br>(31·8, 33·7)	4150<br>(3720, 4614)	17·8<br>(16, 19·9)	-45·4<br>(-51·2, -39·1)
**Uruguay**	833<br>(803, 866)	20·8<br>(20·1, 21·6)	757<br>(681, 835)	13·5<br>(12·1, 14·9)	-35·1<br>(-42·3, -27·9)
**Eastern Europe**	76543<br>(75068, 77816)	26·6<br>(26, 27)	43943<br>(42870, 45173)	12·8<br>(12·5, 13·1)	-51·9<br>(-52·9, -50·8)
**Belarus**	4022<br>(3910, 4129)	30·3<br>(29·5, 31·1)	2123<br>(1968, 2280)	13·3<br>(12·3, 14·2)	-56·2<br>(-59·5, -53)
**Estonia**	536<br>(521, 553)	25·6<br>(24·8, 26·3)	289<br>(253, 326)	10·8<br>(9·5, 12·3)	-57·7<br>(-63, -52·2)
**Latvia**	882<br>(858, 907)	23·9<br>(23·2, 24·5)	489<br>(438, 540)	12·1<br>(10·8, 13·4)	-49·3<br>(-54·7, -43·6)
**Lithuania**	1123<br>(1092, 1155)	24·1<br>(23·4, 24·8)	742<br>(691, 798)	12·8<br>(11·9, 13·8)	-46·8<br>(-50·6, -42·8)
**Moldova**	816<br>(790, 846)	17·7<br>(17·2, 18·3)	505<br>(478, 534)	8·9<br>(8·4, 9·4)	-49·8<br>(-52·9, -46·6)
**Russia**	51964<br>(50970, 52899)	27·9<br>(27·4, 28·4)	30566<br>(29862, 31332)	13·1<br>(12·8, 13·4)	-53·1<br>(-53·9, -52·1)
**Ukraine**	17198<br>(16629, 17749)	23·4<br>(22·7, 24·1)	9230<br>(8725, 9828)	12·2<br>(11·5, 13)	-48<br>(-51·2, -44·6)
**Central Europe**	25293<br>(24762, 25750)	16·8<br>(16·5, 17·1)	18570<br>(18014, 19135)	8·6<br>(8·4, 8·9)	-48·7<br>(-50·5, -46·8)
**Albania**	306<br>(289, 323)	14·1<br>(13·3, 14·9)	446<br>(369, 535)	10·6<br>(8·8, 12·7)	-24·4<br>(-37·9, -7·9)
**Bosnia and Herzegovina**	518<br>(494, 544)	12·7<br>(12·1, 13·3)	580<br>(531, 628)	9·7<br>(8·9, 10·5)	-23·8<br>(-31·2, -16·1)
**Bulgaria**	2220<br>(2131, 2309)	17·4<br>(16·7, 18)	1343<br>(1246, 1441)	9·1<br>(8·4, 9·7)	-47·8<br>(-51·9, -43·2)
**Croatia**	1354<br>(1301, 1402)	21<br>(20·2, 21·7)	806<br>(758, 860)	8·9<br>(8·4, 9·5)	-57·5<br>(-60·4, -54)
**Czech Republic**	2504<br>(2434, 2577)	17·9<br>(17·4, 18·4)	1251<br>(1168, 1340)	6<br>(5·6, 6·4)	-66·5<br>(-68·9, -64·1)
**Hungary**	3002<br>(2884, 3122)	19·9<br>(19·1, 20·7)	1621<br>(1516, 1725)	8·3<br>(7·7, 8·8)	-58·4<br>(-61·9, -54·7)
**Macedonia**	359<br>(334, 384)	19·1<br>(17·8, 20·5)	387<br>(354, 424)	11·4<br>(10·4, 12·5)	-40·4<br>(-47·1, -33·2)
**Montenegro**	47<br>(42, 55)	7·6<br>(6·7, 8·7)	62<br>(56, 69)	6·2<br>(5·5, 6·9)	-18·8<br>(-32·8, -3·6)
**Poland**	7973<br>(7781, 8165)	17·6<br>(17·2, 18)	6178<br>(5776, 6580)	8·8<br>(8·3, 9·4)	-49·7<br>(-52·9, -46·3)
**Romania**	4051<br>(3874, 4209)	14·2<br>(13·6, 14·7)	3516<br>(3310, 3743)	9·5<br>(8·9, 10·1)	-33·1<br>(-37·9, -27·6)

**Serbia**	1441 (1288, 1630)	12·7 (11·4, 14·2)	1302 (1204, 1405)	8·1 (7·5, 8·7)	-36 (-44·4, -26·8)
**Slovakia**	1021 (980, 1062)	16·8 (16·2, 17·5)	716 (655, 785)	7·9 (7·3, 8·7)	-52·8 (-57·3, -48)
**Slovenia**	498 (479, 517)	20·1 (19·3, 20·8)	363 (336, 392)	8·2 (7·6, 8·9)	-59·1 (-62·5, -55·3)
**Central Asia**	11278 (11040, 11524)	23·5 (23, 24)	10331 (9891, 10769)	14·3 (13·8, 14·9)	-39 (-41·7, -36·4)
**Armenia**	563 (535, 593)	20·3 (19·4, 21·3)	527 (498, 554)	12·7 (12, 13·3)	-37·7 (-41·9, -32·8)
**Azerbaijan**	1216 (1155, 1279)	23·6 (22·5, 24·8)	1740 (1530, 1947)	20·2 (17·9, 22·5)	-14·5 (-25, -3·5)
**Georgia**	937 (887, 989)	14·9 (14·1, 15·7)	851 (806, 902)	14·3 (13·5, 15·1)	-3·9 (-11·4, 4)
**Kazakhstan**	3884 (3755, 4013)	29·6 (28·6, 30·6)	2182 (2029, 2343)	13 (12·1, 13·9)	-56·1 (-59, -53)
**Kyrgyzstan**	873 (829, 923)	28·3 (26·9, 29·9)	616 (580, 655)	14·2 (13·4, 15·1)	-49·8 (-53·2, -45·8)
**Mongolia**	640 (591, 695)	62·8 (58·4, 67·9)	760 (680, 849)	37·6 (33·8, 41·8)	-40·1 (-47·2, -32·5)
**Tajikistan**	680 (652, 708)	23·8 (22·8, 24·8)	904 (811, 995)	17·8 (16, 19·7)	-24·9 (-33, -16·5)
**Turkmenistan**	385 (371, 399)	19·7 (19, 20·4)	365 (339, 398)	9·8 (9·1, 10·6)	-50·2 (-54·2, -45·5)
**Uzbekistan**	2101 (2028, 2180)	18 (17·4, 18·7)	2385 (2135, 2683)	10·9 (9·8, 12·2)	-39·3 (-45·9, -31·4)
**Central Latin America**	12960 (12786, 13135)	16 (15·7, 16·2)	21226 (20432, 22007)	9·3 (9, 9·7)	-41·6 (-43·8, -39·4)
**Colombia**	4108 (4006, 4207)	24·4 (23·8, 25)	5761 (5186, 6385)	10·7 (9·6, 11·8)	-56·2 (-60·5, -51·4)
**Costa Rica**	591 (575, 608)	34·4 (33·4, 35·3)	838 (784, 895)	17·2 (16·1, 18·4)	-49·8 (-53·5, -46)
**El Salvador**	406 (384, 431)	13·9 (13·1, 14·7)	773 (653, 895)	13·5 (11·4, 15·6)	-2·7 (-17·4, 13·2)
**Guatemala**	757 (734, 781)	22·7 (21·9, 23·4)	2025 (1832, 2219)	19·4 (17·6, 21·2)	-14·5 (-23·1, -5·6)
**Honduras**	173 (151, 195)	8·4 (7·4, 9·4)	411 (338, 495)	7·3 (6, 8·8)	-13·3 (-29·2, 8·4)
**Mexico**	4828 (4754, 4904)	11·6 (11·4, 11·7)	8118 (7942, 8320)	7·3 (7·1, 7·5)	-37 (-38·7, -35·2)
**Nicaragua**	203 (188, 221)	12·9 (11·9, 14)	357 (318, 399)	8 (7·1, 8·9)	-38·3 (-46·2, -29·8)
**Panama**	198 (190, 206)	13·3 (12·8, 13·9)	346 (322, 369)	8·7 (8·1, 9·3)	-34·5 (-39·6, -29·2)
**Venezuela**	1694 (1640, 1750)	18·2 (17·7, 18·8)	2598 (2267, 2960)	9·7 (8·4, 11)	-46·9 (-53·6, -39·4)
**Andean Latin America**	5351 (5110, 5603)	26·7 (25·5, 27·9)	9130 (8408, 9901)	17·1 (15·7, 18·5)	-35·9 (-41·4, -29·7)
**Bolivia**	1391 (1226, 1590)	45·1 (40·1, 51·2)	2199 (1813, 2655)	27·1 (22·5, 32·6)	-39·9 (-51, -25·8)
**Ecuador**	1516 (1479, 1557)	29 (28·2, 29·8)	2579 (2360, 2828)	18 (16·5, 19·7)	-38 (-43·4, -31·6)
**Peru**	2445 (2265, 2618)	20·8 (19·3, 22·3)	4353 (3795, 4919)	14·2 (12·3, 16)	-32 (-41·7, -20·2)
**Caribbean**	2862 (2722, 3022)	11 (10·5, 11·6)	3684 (3438, 3966)	7·3 (6·8, 7·8)	-34·1 (-38·6, -29·3)
**Antigua and Barbuda**	8 (7, 8)	14·4 (13·5, 15·3)	9 (8, 9)	8·7 (8·1, 9·4)	-39·4 (-44·7, -33·6)
**The Bahamas**	19 (18, 21)	12·6 (11·9, 13·3)	29 (26, 32)	8·1 (7·4, 8·9)	-35·5 (-41·6, -28)
**Barbados**	48 (46, 51)	15·3 (14·6, 16)	41 (37, 45)	8·3 (7·7, 9·1)	-45·4 (-50·6, -39·3)

**Belize**	11 (10, 12)	12·1 (11·2, 13·1)	22 (21, 23)	8·6 (8·1, 9·2)	-28·8 (-35·8, -21·2)
**Bermuda**	7 (7, 8)	11·3 (10·6, 12·1)	6 (6, 7)	5 (4·6, 5·4)	-56·3 (-60·6, -51·8)
**Cuba**	787 (763, 812)	7·6 (7·3, 7·8)	986 (890, 1097)	5·2 (4·7, 5·8)	-31·2 (-38, -23·4)
**Dominica**	16 (15, 17)	21·9 (20·7, 23·2)	15 (14, 16)	15·9 (14·7, 17·1)	-27·6 (-33·8, -20·8)
**Dominican Republic**	271 (249, 294)	7·3 (6·7, 7·9)	651 (562, 742)	7·1 (6·1, 8·1)	-2·4 (-17·2, 14·2)
**Grenada**	10 (10, 11)	14·1 (13·3, 15)	14 (13, 14)	8·4 (7·8, 9)	-40·4 (-45·5, -34·7)
**Guyana**	40 (38, 42)	10·7 (10·1, 11·2)	40 (35, 44)	6·8 (6·1, 7·6)	-36 (-43·4, -27·8)
**Haiti**	743 (617, 891)	24·4 (20·5, 29·2)	994 (800, 1221)	16·2 (13·2, 19·7)	-33·5 (-45·2, -19·3)
**Jamaica**	256 (241, 272)	13·8 (13, 14·7)	267 (232, 309)	9·2 (8, 10·7)	-33·5 (-43·2, -22·1)
**Puerto Rico**	399 (385, 414)	10·7 (10·3, 11·1)	313 (291, 335)	4·3 (4, 4·6)	-60·2 (-63·3, -56·8)
**Saint Lucia**	16 (15, 17)	18 (17·1, 18·9)	21 (20, 23)	10·2 (9·5, 10·9)	-43·4 (-47·9, -38·4)
**Saint Vincent and the Grenadines**	10 (10, 11)	13·9 (13·1, 14·7)	13 (12, 14)	9·6 (8·9, 10·3)	-31·1 (-37·2, -24·7)
**Suriname**	23 (21, 25)	9·4 (8·7, 10)	36 (32, 39)	6·4 (5·7, 7)	-31·9 (-40·1, -23·1)
**Trinidad and Tobago**	91 (86, 95)	10·9 (10·4, 11·4)	80 (67, 94)	4·6 (3·8, 5·4)	-57·8 (-64·9, -49·6)
**Virgin Islands**	9 (9, 10)	11·4 (10·5, 12·2)	16 (13, 18)	8·6 (7·2, 9·6)	-24·6 (-37·5, -13·1)
**Tropical Latin America**	14695 (14434, 14953)	16·8 (16·5, 17·1)	21140 (20734, 21550)	9·3 (9·1, 9·4)	-44·9 (-46·3, -43·6)
**Brazil**	14453 (14193, 14707)	16·9 (16·6, 17·2)	20708 (20316, 21124)	9·3 (9·1, 9·5)	-45·2 (-46·6, -44)
**Paraguay**	242 (223, 262)	11·4 (10·5, 12·3)	432 (367, 516)	8·4 (7·2, 10)	-25·8 (-38·3, -10·4)
**East Asia**	295718 (284777, 312773)	33·6 (32·4, 35·7)	371288 (356398, 387201)	18·7 (17·9, 19·5)	-44·5 (-48·7, -41.0)
**China**	284772 (274485, 301470)	34·2 (33, 36·2)	355379 (340850, 371542)	18·8 (18·1, 19·7)	-44·9 (-49·4, -41·4)
**North Korea**	3484 (2869, 4142)	21·3 (17·6, 25)	6401 (5220, 7708)	20·6 (16·9, 24·8)	-3·6 (-24·9, 21·8)
**Taiwan (Province of China)**	2535 (2462, 2607)	16·3 (15·9, 16·7)	3527 (3320, 3745)	9·2 (8·6, 9·7)	-43·8 (-47·2, -39·8)
**Southeast Asia**	31675 (29163, 34305)	12·7 (11·7, 13·8)	38871 (36283, 41869)	7 (6·6, 7·6)	-44·7 (-49·7, -39·4)
**Cambodia**	895 (736, 1064)	20·3 (16·7, 24·1)	1069 (915, 1268)	10·2 (8·9, 12)	-49·5 (-57·4, -39·6)
**Indonesia**	10380 (9179, 11593)	11·1 (9·8, 12·6)	15210 (13759, 17001)	8 (7·3, 8·9)	-27·6 (-36, -18·7)
**Laos**	393 (317, 477)	19·3 (15·7, 23·2)	352 (289, 426)	9·1 (7·5, 10·9)	-52·9 (-62·6, -41·5)
**Malaysia**	997 (909, 1090)	12 (11, 13·1)	1599 (1409, 1799)	7·1 (6·3, 8)	-40·6 (-49·9, -30·8)
**Maldives**	7 (6, 8)	8 (6·8, 9·2)	6 (6, 7)	2·4 (2·2, 2·6)	-70·3 (-75·5, -63·9)
**Mauritius**	96 (91, 100)	13·6 (12·9, 14·3)	104 (96, 112)	6·6 (6·1, 7·1)	-51·8 (-56·2, -47·3)
**Myanmar**	5261 (4278, 6433)	23·1 (19, 28·1)	4489 (3815, 5370)	10·7 (9·1, 12·7)	-53·7 (-62·9, -43·9)
**Philippines**	1950 (1839, 2066)	7 (6·6, 7·4)	2722 (2378, 3093)	4·2 (3·7, 4·7)	-40·3 (-48·1, -31·1)

**Sri Lanka**	861<br>(794, 929)	8·6<br>(7·9, 9·3)	938<br>(783, 1099)	4·1<br>(3·4, 4·7)	-52·6<br>(-61·2, -43·4)
**Seychelles**	7<br>(6, 7)	11·4<br>(10·4, 12·5)	6<br>(5, 6)	5·5<br>(5·1, 6)	-51·5<br>(-57·3, -44·8)
**Thailand**	3179<br>(2930, 3430)	9·1<br>(8·4, 9·8)	4209<br>(3767, 4756)	4·4<br>(3·9, 4·9)	-52·1<br>(-58·4, -44·3)
**East Timor**	36<br>(29, 44)	13·6<br>(11·2, 16·4)	65<br>(52, 80)	8·8<br>(7·2, 10·8)	-35<br>(-48·1, -19·2)
**Vietnam**	7574<br>(6692, 8504)	18·3<br>(16·2, 20·6)	8051<br>(7027, 9272)	8·9<br>(7·9, 10·2)	-51·2<br>(-59·7, -41·3)
**Oceania**	483<br>(407, 562)	16·4<br>(14·2, 18·8)	913<br>(758, 1077)	14<br>(11·9, 16·1)	-14·5<br>(-24·8, -3·4)
**American Samoa**	4<br>(3, 4)	16·6<br>(15, 18·1)	5<br>(4, 5)	12·9<br>(11·4, 14·4)	-22·2<br>(-33·4, -9·9)
**Federated States of Micronesia**	7<br>(6, 9)	15·8<br>(13·5, 18·4)	7<br>(6, 9)	12·1<br>(10·3, 14·1)	-23·9<br>(-36·9, -7·5)
**Fiji**	22<br>(20, 25)	6·8<br>(6, 7·5)	37<br>(32, 42)	5·8<br>(5·1, 6·6)	-13·5<br>(-26·5, 3·2)
**Guam**	4<br>(4, 5)	6·1<br>(5·7, 6·6)	8<br>(8, 9)	5<br>(4·5, 5·4)	-19·4<br>(-28·3, -9·2)
**Kiribati**	6<br>(5, 6)	15·5<br>(14·1, 17·1)	7<br>(6, 8)	11·8<br>(10·3, 13·5)	-23·8<br>(-35, -10·8)
**Marshall Islands**	3<br>(3, 3)	19·2<br>(17, 21·6)	5<br>(4, 5)	15·7<br>(13·5, 18·3)	-18<br>(-29·3, -4·9)
**Northern Mariana Islands**	2<br>(1, 2)	10·6<br>(9·3, 12·3)	3<br>(3, 3)	6·6<br>(6, 7·4)	-37·6<br>(-48·3, -25·9)
**Papua New Guinea**	352<br>(285, 421)	18·9<br>(15·6, 22·3)	705<br>(564, 856)	16·2<br>(13·2, 19·2)	-14·6<br>(-27·3, -0·5)
**Samoa**	10<br>(8, 11)	12·1<br>(10·3, 14·1)	14<br>(12, 16)	10·8<br>(9·3, 12·4)	-10·7<br>(-25·3, 6·3)
**Solomon Islands**	23<br>(19, 28)	17<br>(14·2, 20·3)	40<br>(33, 48)	13<br>(11, 15·4)	-23·5<br>(-35·3, -9·1)
**Tonga**	9<br>(8, 10)	18·5<br>(17·1, 20·1)	11<br>(10, 13)	14·4<br>(12·7, 16·4)	-22·1<br>(-33·5, -8·2)
**Vanuatu**	10<br>(8, 13)	16·5<br>(13·1, 20·2)	21<br>(17, 27)	14<br>(11·1, 17·5)	-15<br>(-31·7, 5·8)
**North Africa and Middle East**	23487<br>(21315, 25441)	14<br>(12·8, 15·2)	34530<br>(32838, 36201)	8·8<br>(8·4, 9·2)	-37·4<br>(-42·1, -31·9)
**Afghanistan**	2539<br>(1505, 3343)	36·9<br>(22·5, 48·3)	3614<br>(2880, 4485)	33·6<br>(27·2, 40·2)	-9·1<br>(-27·4, 40·5)
**Algeria**	899<br>(800, 995)	7·6<br>(6·8, 8·3)	1428<br>(1300, 1563)	4·6<br>(4·2, 5)	-39·8<br>(-46·3, -31·5)
**Bahrain**	18<br>(17, 19)	11·2<br>(10·4, 12·2)	30<br>(27, 33)	3·9<br>(3·5, 4·4)	-64·8<br>(-69·2, -59·9)
**Egypt**	1211<br>(1106, 1336)	4·9<br>(4·6, 5·4)	1864<br>(1655, 2091)	3·7<br>(3·3, 4·2)	-24·3<br>(-34, -11·8)
**Iran**	5298<br>(4875, 5724)	21·9<br>(20·2, 23·6)	9681<br>(9363, 10043)	14·9<br>(14·4, 15·4)	-31·9<br>(-36·8, -25·9)
**Iraq**	578<br>(491, 676)	7·5<br>(6·4, 8·7)	661<br>(613, 717)	2·9<br>(2·7, 3·2)	-61·1<br>(-67·4, -53·5)
**Jordan**	104<br>(90, 120)	7·6<br>(6·5, 8·6)	217<br>(192, 246)	4·3<br>(3·8, 4·9)	-42·8<br>(-53, -30·9)
**Kuwait**	27<br>(25, 29)	4·4<br>(4·1, 4·7)	48<br>(44, 52)	2·1<br>(1·9, 2·3)	-53·2<br>(-58·1, -47·7)
**Lebanon**	259<br>(221, 296)	12·1<br>(10·4, 13·7)	343<br>(309, 377)	6<br>(5·4, 6·6)	-50·3<br>(-58·1, -40·1)
**Libya**	134<br>(115, 157)	7·3<br>(6·2, 8·5)	279<br>(241, 321)	6·4<br>(5·6, 7·3)	-11·6<br>(-29·2, 10·1)
**Morocco**	763<br>(693, 844)	5·6<br>(5·1, 6·1)	1200<br>(1014, 1409)	4·1<br>(3·5, 4·8)	-26·4<br>(-39·8, -10·7)
**Palestine**	83<br>(69, 99)	9·2<br>(7·7, 10·9)	126<br>(116, 137)	5·4<br>(4·9, 5·9)	-41·8<br>(-52·3, -28·9)

**Oman**	93<br>(76, 112)	13·9<br>(11·5, 16·5)	119<br>(99, 140)	6·8<br>(5·8, 8)	-50·7<br>(-60·4, -37·5)
**Qatar**	10<br>(9, 11)	11·1<br>(9·7, 12·7)	35<br>(29, 42)	5·1<br>(4·3, 6·1)	-53·6<br>(-62·6, -42·3)
**Saudi Arabia**	440<br>(364, 541)	7·6<br>(6·3, 9·3)	614<br>(544, 701)	4·7<br>(4·3, 5·3)	-38<br>(-51, -22·8)
**Sudan**	1705<br>(1303, 2094)	18·6<br>(14·4, 22·7)	2539<br>(1960, 3156)	14·9<br>(11·6, 18·4)	-19·8<br>(-37·2, 2·7)
**Syria**	318<br>(283, 354)	6·4<br>(5·7, 7·1)	498<br>(426, 580)	4·3<br>(3·7, 5)	-32·9<br>(-44·9, -19)
**Tunisia**	331<br>(299, 367)	7·2<br>(6·6, 8)	578<br>(472, 694)	5<br>(4·1, 6)	-30·7<br>(-44·6, -13·5)
**Turkey**	7527<br>(6792, 8278)	20·7<br>(18·6, 22·8)	8022<br>(7237, 8790)	9·2<br>(8·3, 10·1)	-55·3<br>(-61·1, -48·6)
**United Arab Emirates**	35<br>(28, 43)	10·2<br>(8·2, 12·5)	232<br>(179, 290)	8·8<br>(7·1, 10·7)	-13·9<br>(-35·2, 16·8)
**Yemen**	1099<br>(737, 1478)	22<br>(15·3, 29)	2371<br>(1785, 2991)	19·6<br>(15·1, 24·3)	-11·1<br>(-33·4, 26·8)
**South Asia**	62585<br>(58209, 67100)	10·7<br>(9·9, 11·5)	96652<br>(91276, 101052)	7·5<br>(7, 7·8)	-30·2<br>(-36·6, -24·6)
**Bangladesh**	6112<br>(5238, 7278)	12·9<br>(11·1, 15·3)	7688<br>(6467, 9028)	6·5<br>(5·5, 7·7)	-49·5<br>(-61·3, -37·4)
**Bhutan**	26<br>(21, 32)	10·4<br>(8·7, 12·8)	35<br>(28, 43)	6·1<br>(4·9, 7·4)	-41·9<br>(-55·4, -26·9)
**India**	52117<br>(48014, 56032)	11·1<br>(10·1, 11·9)	81573<br>(76969, 85604)	7·7<br>(7·3, 8·1)	-30<br>(-36·3, -23·6)
**Nepal**	1102<br>(915, 1330)	11·6<br>(9·7, 14)	1673<br>(1410, 1977)	8·1<br>(6·9, 9·6)	-30·1<br>(-43·3, -15·1)
**Pakistan**	3227<br>(2908, 3604)	5·8<br>(5·2, 6·4)	5682<br>(4729, 6729)	5·5<br>(4·6, 6·5)	-4·1<br>(-21·1, 15·3)
**Southern sub-Saharan Africa**	2056<br>(1936, 2199)	7·5<br>(7, 8)	2910<br>(2779, 3052)	5·5<br>(5·3, 5·8)	-26·2<br>(-31·3, -21)
**Botswana**	45<br>(39, 53)	8·3<br>(7·2, 9·6)	60<br>(53, 69)	5<br>(4·4, 5·7)	-40·2<br>(-49·8, -28·1)
**Lesotho**	104<br>(90, 118)	11<br>(9·6, 12·5)	114<br>(95, 134)	10·2<br>(8·5, 11·9)	-7·9<br>(-24·2, 11·5)
**Namibia**	40<br>(36, 45)	6<br>(5·4, 6·7)	45<br>(39, 52)	3·5<br>(3, 4)	-42·8<br>(-51·7, -32·2)
**South Africa**	1402<br>(1303, 1497)	6·6<br>(6·1, 7)	1940<br>(1867, 2022)	4·6<br>(4·5, 4·8)	-29·8<br>(-34, -24·7)
**Swaziland**	29<br>(25, 33)	10·4<br>(9, 12)	41<br>(34, 49)	7·8<br>(6·5, 9·3)	-25·3<br>(-40, -8)
**Zimbabwe**	437<br>(383, 489)	11·1<br>(9·8, 12·4)	710<br>(613, 814)	11·1<br>(9·7, 12·7)	0·3<br>(-17·2, 20)
**Western sub-Saharan Africa**	8626<br>(7794, 9504)	10·4<br>(9·5, 11·5)	13311<br>(12153, 14701)	8·4<br>(7·7, 9·2)	-19·5<br>(-27·1, -9·9)
**Benin**	301<br>(266, 342)	15·5<br>(13·7, 17·5)	514<br>(432, 606)	12·5<br>(10·6, 14·6)	-19·7<br>(-33·9, -3·3)
**Burkina Faso**	668<br>(543, 775)	16·6<br>(13·6, 19·1)	1063<br>(912, 1226)	13·7<br>(11·8, 15·7)	-17·4<br>(-31·3, 4·5)
**Cameroon**	635<br>(547, 714)	15·7<br>(13·4, 17·6)	1248<br>(1018, 1498)	12·6<br>(10·5, 15·1)	-19·4<br>(-33·9, -3·3)
**Cape Verde**	62<br>(56, 67)	26·5<br>(24·3, 28·7)	76<br>(70, 84)	17·1<br>(15·5, 18·9)	-35·3<br>(-42·5, -27)
**Chad**	386<br>(336, 442)	14·1<br>(12·3, 16·1)	702<br>(587, 825)	14·6<br>(12·3, 17)	4<br>(-12·5, 25·3)
**Côte d'Ivoire**	252<br>(219, 286)	7·2<br>(6·3, 8)	507<br>(433, 595)	5·9<br>(5·1, 6·8)	-17·9<br>(-31·9, -1·8)
**The Gambia**	25<br>(21, 29)	7·9<br>(6·7, 9·2)	52<br>(45, 60)	6·2<br>(5·4, 7·1)	-20·7<br>(-34·6, -4·4)
**Ghana**	664<br>(565, 765)	12<br>(10·3, 13·7)	1096<br>(949, 1251)	8·4<br>(7·3, 9·4)	-30·1<br>(-41·8, -16·5)

**Guinea**	492 (438, 553)	15 (13·4, 16·8)	794 (680, 920)	15·3 (13·1, 17·7)	2·2 (-16·1, 22·4)
**Guinea-Bissau**	86 (72, 103)	22·8 (19·2, 26·9)	99 (84, 116)	16·2 (13·9, 18·7)	-29 (-40·7, -14·3)
**Liberia**	147 (126, 171)	13·6 (11·8, 15·6)	196 (167, 231)	11·7 (10, 13·6)	-14·2 (-28·5, 3·7)
**Mali**	937 (832, 1053)	23·8 (21·2, 26·6)	1205 (1009, 1418)	15·4 (13, 18)	-35·1 (-45·4, -22·2)
**Mauritania**	153 (134, 173)	15·5 (13·7, 17·6)	187 (155, 222)	10·5 (8·7, 12·4)	-32·4 (-43·7, -19·2)
**Niger**	404 (323, 481)	15·7 (12·8, 18·6)	802 (650, 958)	13 (10·8, 15·4)	-17 (-30·6, 1·9)
**Nigeria**	2493 (1906, 3180)	6·2 (4·8, 7·7)	3141 (2339, 4302)	4·5 (3·4, 6)	-27·4 (-46·4, -0·5)
**São Tomé and Príncipe**	10 (9, 11)	15·8 (14·3, 17·6)	15 (13, 18)	17·1 (14·2, 20·2)	8·3 (-12·7, 31·9)
**Senegal**	473 (416, 538)	15·6 (13·7, 17·6)	843 (720, 974)	13·1 (11·1, 15)	-16·1 (-29, -1·5)
**Sierra Leone**	274 (217, 327)	14·5 (11·7, 17·2)	406 (346, 471)	13·3 (11·5, 15·4)	-8·1 (-25·3, 19·4)
**Togo**	164 (144, 186)	14·4 (12·7, 16·2)	362 (300, 429)	12·3 (10·3, 14·3)	-14·9 (-29·3, 3)
**Eastern sub-Saharan Africa**	8238 (7265, 9178)	11·1 (10, 12·3)	10087 (9391, 10828)	6·8 (6·3, 7·3)	-39·3 (-45·3, -32·5)
**Burundi**	294 (244, 361)	13·7 (11·6, 16·7)	305 (249, 370)	7·9 (6·6, 9·4)	-42·3 (-52·7, -31·8)
**Comoros**	22 (19, 26)	11·2 (9·5, 13·2)	28 (24, 34)	6·6 (5·6, 7·8)	-40·8 (-52·1, -28)
**Djibouti**	14 (10, 19)	9·7 (7·5, 12·9)	34 (24, 45)	6·5 (4·8, 8·4)	-33·4 (-50·7, -9·8)
**Eritrea**	159 (124, 192)	16·5 (13·8, 19·5)	214 (176, 256)	9·6 (8, 11·2)	-42·1 (-52·7, -28·1)
**Ethiopia**	2911 (2326, 3629)	14·6 (12, 17·9)	2210 (1945, 2621)	5·8 (5·1, 6·9)	-60·3 (-68·1, -51·2)
**Kenya**	845 (730, 966)	10·4 (9, 11·9)	1913 (1731, 2151)	9·5 (8·6, 10·8)	-8·6 (-17·1, -0·9)
**Madagascar**	497 (439, 562)	9·7 (8·6, 11)	707 (575, 845)	7 (5·8, 8·3)	-27·8 (-40·6, -13·3)
**Malawi**	207 (140, 250)	5·7 (4·1, 6·7)	246 (212, 281)	3·6 (3·1, 4·1)	-37·2 (-48·6, -11·6)
**Mozambique**	488 (420, 562)	8·9 (7·8, 10·3)	702 (594, 813)	7·2 (6·2, 8·3)	-19 (-33·7, 0·2)
**Rwanda**	390 (324, 452)	13·7 (11·4, 15·7)	315 (261, 371)	6 (5, 6·9)	-56·6 (-65·3, -47·7)
**Somalia**	308 (194, 429)	12·5 (8·8, 16·5)	557 (426, 720)	9·2 (7·2, 11·6)	-26·1 (-45·7, 6·1)
**South Sudan**	260 (178, 359)	11·3 (8·2, 15·1)	300 (226, 391)	8·4 (6·5, 10·8)	-25·4 (-45·9, 6·7)
**Tanzania**	975 (758, 1168)	9·2 (7·5, 10·9)	1402 (1195, 1618)	6·1 (5·2, 7·1)	-33·4 (-45·3, -16·9)
**Uganda**	508 (435, 589)	8·1 (7, 9·4)	701 (604, 803)	5·5 (4·8, 6·3)	-32·2 (-44·2, -17·4)
**Zambia**	356 (296, 434)	12·7 (10·8, 15·4)	445 (375, 532)	7·3 (6·2, 8·7)	-42·3 (-52·1, -30·4)
**Central sub-Saharan Africa**	2454 (2104, 2827)	11·5 (9·9, 13·1)	3551 (3110, 3990)	7·6 (6·7, 8·5)	-33·9 (-42·7, -24·2)
**Angola**	529 (416, 635)	14 (11·5, 16·7)	698 (580, 819)	7·2 (6, 8·3)	-48·8 (-58·1, -36·4)
**Central African Republic**	174 (139, 206)	15·5 (13, 18)	222 (175, 268)	10·9 (8·7, 12·9)	-29·6 (-42, -15·4)
**Congo**	145 (126, 165)	13·6 (12, 15·4)	181 (151, 212)	7·8 (6·6, 9)	-42·8 (-52·9, -30·9)

**DR Congo**	1522<br>(1259, 1802)	10·5<br>(8·8, 12·2)	2371<br>(1970, 2775)	7·6<br>(6·4, 8·8)	-27·3<br>(-40·5, -11·1)
**Equatorial Guinea**	29<br>(23, 36)	15·4<br>(12·4, 18·9)	20<br>(14, 28)	4·8<br>(3·6, 6·5)	-68·6<br>(-77·7, -56·2)
**Gabon**	56<br>(45, 66)	10·1<br>(8·3, 11·9)	59<br>(51, 68)	6·1<br>(5·2, 6·9)	-40·1<br>(-50·3, -27·3)

Appendix Table 3. DALYs of stomach cancer in 1990 and 2017 for both sexes and percentage change of age-standardised rates by country and region

	1990		2017		Percentage change in age-standardised rates between 1990 and 2017
	Counts (95% UI)	Rate (95% UI)	Counts (95% UI)	Rate (95% UI)	
**Global**	19143257 (18668093, 19805149)	445·9 (435, 461·2)	19130771 (18738585, 19569409)	235·9 (231·1, 241·3)	-47·1 (-49.0, -45·3)
**High-income North America**	427610 (422515, 433335)	122·6 (121·1, 124·2)	419819 (407273, 431498)	74·5 (72·3, 76·7)	-39·2 (-41·3, -37·2)
**Canada**	67245 (65708, 68965)	204·7 (199·9, 209·9)	76140 (70795, 81983)	120·7 (112·2, 129·9)	-41 (-45·3, -36·4)
**Greenland**	202 (186, 221)	484·1 (446·7, 525·2)	180 (164, 198)	251·8 (229·8, 275·1)	-48 (-54, -41·4)
**USA**	360153 (355718, 365242)	114 (112·6, 115·6)	343491 (332586, 353797)	68·9 (66·6, 71)	-39·6 (-41·9, -37·3)
**Australasia**	40199 (39330, 41106)	168·7 (165·1, 172·4)	39703 (36268, 43401)	88·4 (80·7, 96·6)	-47·6 (-52·2, -42·5)
**Australia**	32169 (31325, 32979)	161·9 (157·8, 165·9)	33169 (29845, 36799)	87·5 (78·5, 97)	-46 (-51·4, -39·9)
**New Zealand**	8030 (7748, 8325)	203·5 (196·6, 210·7)	6534 (6125, 7022)	93·4 (87·8, 100·1)	-54·1 (-57·2, -50·6)
**High-income Asia-Pacific**	1553346 (1531220, 1575287)	743·9 (733·5, 754·3)	1099094 (1056842, 1147281)	280 (268·6, 292·3)	-62·4 (-63·8, -60·6)
**Brunei**	652 (591, 714)	510·9 (463·5, 557·6)	882 (796, 975)	244·3 (222·5, 270·3)	-52·2 (-57·9, -45·8)
**Japan**	1118980 (1100943, 1136136)	648·6 (638, 658·3)	842005 (812715, 877828)	275·3 (265·7, 286·3)	-57·6 (-59, -55·7)
**Singapore**	9047 (8727, 9387)	364·6 (352·3, 377·9)	6737 (6228, 7269)	97·5 (90·1, 105·2)	-73·3 (-75·5, -71)
**South Korea**	424666 (413797, 435760)	1185·4 (1156·6, 1215·1)	249470 (227327, 271801)	297·4 (271·7, 324·1)	-74·9 (-77·2, -72·5)
**Western Europe**	1593267 (1576488, 1610918)	279·3 (276·3, 282·3)	1025787 (984851, 1066633)	125·4 (120·2, 130·4)	-55·1 (-56·9, -53·3)
**Andorra**	108 (94, 124)	179·1 (156·3, 206)	141 (122, 162)	107·5 (93, 123·3)	-40 (-51, -29)
**Austria**	35718 (34806, 36632)	309·8 (301·8, 317·7)	18336 (17041, 19638)	111·4 (103·3, 119·8)	-64 (-66·6, -61·3)
**Belgium**	33952 (32829, 35070)	221·6 (214·5, 228·7)	20702 (19196, 22366)	98·3 (90·9, 106·3)	-55·6 (-59·2, -51·7)
**Cyprus**	1470 (1345, 1611)	172·2 (157·8, 188·7)	2324 (2057, 2603)	125·4 (111·1, 140·3)	-27·2 (-37·2, -15·9)
**Denmark**	14110 (13703, 14517)	183 (177·8, 188·4)	10095 (9382, 10886)	96·2 (89·3, 103·7)	-47·4 (-51·4, -43)
**Finland**	18500 (18016, 19005)	261·7 (254·9, 268·7)	10044 (9330, 10855)	93·1 (86·5, 100·7)	-64·4 (-67·2, -61·4)
**France**	169356 (164815, 174674)	207·3 (201·8, 213·5)	117964 (108987, 127303)	98·2 (90·7, 106)	-52·6 (-56·4, -48·6)
**Germany**	367807 (358745, 377109)	297·1 (289·9, 304·8)	230923 (208347, 257782)	138·5 (124·4, 154·3)	-53·4 (-58·2, -47·9)
**Greece**	34146 (33049, 35316)	226·7 (219·7, 234·1)	31690 (29493, 34231)	153·8 (143·1, 166·2)	-32·1 (-37·2, -26·5)
**Iceland**	753 (717, 792)	265·4 (252·5, 279·5)	483 (452, 517)	95·4 (89·1, 101·9)	-64·1 (-66·8, -61·1)
**Ireland**	10573 (10205, 10963)	255·2 (246·3, 264·7)	8138 (7481, 8885)	114·8 (105·5, 125)	-55 (-58·9, -50·9)
**Israel**	10127 (9727, 10515)	206 (197·9, 213·7)	12117 (11234, 13102)	114·2 (105·9, 123·6)	-44·6 (-48·9, -39·9)
**Italy**	329721 (321629, 337868)	372·7 (363·8, 381·6)	190697 (176375, 206017)	147·7 (136·5, 159·7)	-60·4 (-63·4, -57·2)

**Luxembourg**	1328<br>(1266, 1394)	242·8<br>(231·6, 254·6)	928<br>(820, 1038)	99·7<br>(88·1, 111·6)	-58·9<br>(-64·1, -53·7)
**Malta**	998<br>(947, 1053)	227·7<br>(216·3, 240·1)	978<br>(911, 1053)	116·3<br>(108·5, 125)	-48·9<br>(-53, -44·5)
**Netherlands**	50963<br>(49633, 52371)	255<br>(248·5, 261·9)	39643<br>(37104, 42303)	124·6<br>(116·6, 133·2)	-51·1<br>(-54·4, -47·7)
**Norway**	14287<br>(14062, 14531)	221·2<br>(217·7, 224·9)	7527<br>(7242, 7857)	85·3<br>(82·2, 88·9)	-61·4<br>(-62·9, -59·7)
**Portugal**	74142<br>(72266, 76302)	544·5<br>(530·8, 559·9)	50147<br>(46179, 53700)	241·9<br>(221·9, 260)	-55·6<br>(-59·4, -52)
**Spain**	161553<br>(157354, 165506)	298·3<br>(290·9, 305·4)	117793<br>(109608, 126461)	136·4<br>(126·9, 146·7)	-54·3<br>(-57·7, -50·6)
**Sweden**	25322<br>(24629, 26005)	175·8<br>(171·2, 180·3)	13402<br>(12654, 14226)	70·9<br>(66·9, 75·3)	-59·7<br>(-62, -57)
**Switzerland**	18601<br>(18022, 19136)	182·9<br>(177·2, 188·4)	11492<br>(10585, 12438)	73·1<br>(67·2, 79·1)	-60<br>(-63·6, -56·4)
**United Kingdom**	218196<br>(215737, 220865)	241·5<br>(238·8, 244·3)	129160<br>(126238, 132447)	109·7<br>(107·2, 112·4)	-54·6<br>(-55·6, -53·4)
**Southern Latin America**	203098<br>(198576, 207625)	425·4<br>(416·1, 434·8)	203392<br>(188154, 220430)	253·8<br>(234·7, 275·1)	-40·4<br>(-45, -35)
**Argentina**	114634<br>(110783, 118629)	345·1<br>(333·5, 356·9)	109141<br>(97552, 122304)	210·8<br>(188·2, 236·3)	-38·9<br>(-45·9, -30·9)
**Chile**	71229<br>(69025, 73387)	677·7<br>(656·7, 697·6)	80238<br>(71663, 89963)	347<br>(310·2, 388·5)	-48·8<br>(-54·4, -42·5)
**Uruguay**	17227<br>(16579, 17908)	446<br>(429·5, 462·8)	14004<br>(12456, 15640)	282<br>(251·3, 315)	-36·8<br>(-43·9, -29)
**Eastern Europe**	1915763<br>(1870174, 1951155)	664·1<br>(648·5, 676·3)	1014257<br>(987340, 1043869)	308·7<br>(300·7, 317·3)	-53·5<br>(-54·5, -52·5)
**Belarus**	100992<br>(98100, 103645)	770·2<br>(749·1, 789·5)	47313<br>(43564, 51039)	309·4<br>(285·3, 333·1)	-59·8<br>(-63, -56·6)
**Estonia**	12635<br>(12223, 13042)	614·8<br>(595, 634·2)	5699<br>(4982, 6490)	244<br>(212·6, 277·8)	-60·3<br>(-65·4, -54·9)
**Latvia**	21115<br>(20465, 21755)	584·6<br>(566·6, 601·5)	9867<br>(8798, 11003)	278·3<br>(247·2, 311·3)	-52·4<br>(-57·6, -46·9)
**Lithuania**	26617<br>(25852, 27421)	580·2<br>(563·5, 598)	15116<br>(14048, 16317)	298·2<br>(276·9, 322·2)	-48·6<br>(-52·2, -44·5)
**Moldova**	22437<br>(21700, 23248)	472·3<br>(457·3, 489·8)	12858<br>(12152, 13652)	231·3<br>(218·3, 244·9)	-51<br>(-54·2, -47·7)
**Russia**	1289473<br>(1257851, 1315916)	684·9<br>(668, 698·9)	695242<br>(678582, 712147)	308·4<br>(301·1, 316)	-55<br>(-55·9, -54)
**Ukraine**	442495<br>(427142, 457174)	617·8<br>(597·3, 638·1)	228162<br>(214961, 242907)	321·4<br>(303·2, 341·9)	-48<br>(-51·2, -44·5)
**Central Europe**	578358<br>(565062, 589710)	379·4<br>(370·9, 386·5)	379211<br>(367809, 391374)	189·9<br>(184, 196)	-50<br>(-51·8, -48)
**Albania**	8034<br>(7595, 8510)	339·9<br>(320·8, 360·4)	9780<br>(8034, 11812)	242·6<br>(200·3, 292·9)	-28·6<br>(-42·1, -12·6)
**Bosnia and Herzegovina**	13524<br>(12806, 14289)	299·8<br>(285·4, 315·5)	12255<br>(11199, 13286)	211·8<br>(193·3, 228·9)	-29·3<br>(-36·5, -21·9)
**Bulgaria**	51241<br>(49091, 53321)	397·7<br>(382·2, 413·6)	27919<br>(25868, 30050)	210·3<br>(194·6, 226·1)	-47·1<br>(-51·6, -42·2)
**Croatia**	30216<br>(29004, 31389)	457·6<br>(440, 474·6)	14964<br>(14037, 15999)	182<br>(170·1, 194·6)	-60·2<br>(-63·2, -56·8)
**Czech Republic**	51386<br>(49903, 52904)	371·3<br>(360·4, 382)	24026<br>(22366, 25778)	124·2<br>(115·5, 133·1)	-66·6<br>(-69·1, -64)
**Hungary**	62555<br>(60193, 65038)	419·5<br>(403·9, 435·6)	31319<br>(29127, 33442)	174·6<br>(162·4, 186·8)	-58·4<br>(-62, -54·5)
**Macedonia**	9058<br>(8438, 9725)	450·4<br>(419·8, 482·4)	8824<br>(8041, 9712)	265·5<br>(242, 291·6)	-41·1<br>(-47·7, -34)
**Montenegro**	1091<br>(951, 1273)	166·8<br>(145·8, 193·5)	1267<br>(1129, 1424)	128·4<br>(114·8, 143·8)	-23<br>(-36·7, -6·7)
**Poland**	181591<br>(176937, 186157)	399·1<br>(388·8, 409)	126466<br>(117733, 134747)	193·8<br>(180·5, 206·8)	-51·5<br>(-54·7, -47·9)

**Romania**	100235 (95989, 104313)	344·7 (330·8, 358·2)	74381 (69934, 79420)	219·3 (205·8, 233·8)	-36·4 (-41·1, -30·9)
**Serbia**	35641 (31717, 40384)	298·6 (267, 335·7)	26791 (24772, 28970)	178·1 (165, 192·2)	-40·3 (-48·7, -31·6)
**Slovakia**	22878 (21938, 23842)	377·3 (361·5, 393·2)	14764 (13461, 16259)	167·1 (152·6, 183·3)	-55·7 (-60·3, -50·6)
**Slovenia**	10910 (10449, 11362)	434·8 (416·8, 452·6)	6455 (5949, 6982)	163 (149·8, 176·3)	-62·5 (-65·8, -58·9)
**Central Asia**	303319 (296618, 309917)	593·4 (580·6, 605·9)	273093 (260966, 285850)	340 (325, 354·6)	-42·7 (-45·3, -40·1)
**Armenia**	15411 (14636, 16250)	513 (488·2, 539·1)	11503 (10877, 12163)	278·8 (264, 294·3)	-45·7 (-49·6, -41·5)
**Azerbaijan**	32633 (30841, 34479)	585·5 (555·1, 616·2)	44165 (38653, 49665)	446·8 (392·7, 499·4)	-23·7 (-33·2, -13·5)
**Georgia**	23905 (22629, 25203)	372·5 (353·5, 392·5)	18848 (17770, 19989)	337·2 (317·8, 357·3)	-9·5 (-16·4, -2)
**Kazakhstan**	104346 (100757, 107913)	743·1 (718·8, 767·4)	55570 (51597, 59892)	308·7 (287·4, 331·7)	-58·5 (-61·3, -55·2)
**Kyrgyzstan**	24354 (23044, 25759)	757·8 (718·7, 799)	16870 (15855, 18038)	350·1 (329·4, 372·5)	-53·8 (-57·4, -49·8)
**Mongolia**	15910 (14642, 17355)	1427·5 (1315·4, 1552·8)	20987 (18685, 23471)	842·9 (754·7, 941)	-41 (-48·1, -33·1)
**Tajikistan**	18370 (17599, 19177)	600·2 (575·2, 625·5)	26260 (23470, 29149)	438·4 (392·7, 485·5)	-27 (-35·1, -18·5)
**Turkmenistan**	10441 (10042, 10849)	486 (467·9, 504·5)	10076 (9295, 11001)	238·9 (221·1, 260)	-50·8 (-54·9, -46·1)
**Uzbekistan**	57949 (55949, 60057)	463·1 (447·5, 479·4)	68814 (61280, 77850)	268·4 (239·2, 302·3)	-42 (-48·6, -33·9)
**Central Latin America**	307213 (303029, 311526)	333·8 (329·2, 338·5)	487298 (468124, 506160)	203·8 (195·8, 211·7)	-38·9 (-41·4, -36·6)
**Colombia**	98967 (96575, 101449)	514·9 (502·1, 527·6)	128665 (114902, 144229)	239·5 (213·9, 268·7)	-53·5 (-58·4, -48)
**Costa Rica**	13205 (12840, 13619)	707·8 (688·4, 728·6)	17828 (16635, 19180)	359·7 (335·4, 386·3)	-49·2 (-53·2, -45)
**El Salvador**	9572 (9046, 10200)	300·5 (283·5, 320·7)	16426 (13853, 19189)	289 (244·1, 337)	-3·8 (-19·6, 14·2)
**Guatemala**	19311 (18702, 20014)	471·4 (456·5, 487·4)	47541 (42779, 52455)	409·2 (368·9, 450·5)	-13·2 (-22·6, -3·6)
**Honduras**	4626 (4010, 5230)	188·4 (163·6, 212·1)	9060 (7371, 11040)	144·9 (118·3, 176·7)	-23·1 (-37·5, -4·4)
**Mexico**	111535 (109715, 113337)	235·5 (231·8, 239·2)	191047 (186393, 196595)	160·4 (156·5, 165)	-31·9 (-33·8, -29·8)
**Nicaragua**	5327 (4882, 5813)	293·2 (268·6, 319·6)	8632 (7616, 9772)	179·5 (158·8, 202·5)	-38·8 (-47, -29·6)
**Panama**	4756 (4552, 4967)	294 (281·2, 306·5)	7585 (7029, 8152)	191·3 (177·5, 205·5)	-34·9 (-40·4, -29·6)
**Venezuela**	39913 (38622, 41338)	380·1 (367·8, 393·5)	60513 (52644, 69530)	208·4 (181·4, 239·1)	-45·2 (-52·7, -37·1)
**Andean Latin America**	128878 (122532, 135382)	573·2 (545·7, 602)	193905 (176702, 210939)	353·5 (322·4, 384·4)	-38·3 (-44·2, -31·9)
**Bolivia**	35202 (30814, 40679)	991·4 (870·4, 1141·6)	48211 (39027, 59099)	544 (441, 663)	-45·1 (-56·8, -31)
**Ecuador**	34905 (34044, 35867)	601·4 (586·1, 617·4)	53419 (48569, 59079)	355·4 (323·3, 392·8)	-40·9 (-46·4, -34·4)
**Peru**	58770 (54104, 63068)	446·4 (411·3, 478·6)	92275 (79543, 105271)	298·9 (257·2, 341·1)	-33 (-43·1, -21·1)
**Caribbean**	65268 (61575, 69462)	238·5 (225·3, 254)	83446 (77493, 90753)	164·4 (152·5, 178·7)	-31·1 (-36·2, -25·2)
**Antigua and Barbuda**	161 (151, 172)	313·4 (293·6, 333·7)	182 (168, 196)	177·9 (164·8, 192)	-43·2 (-48·4, -37·4)

**The Bahamas**	500 (471, 529)	290·2 (273·7, 306·7)	720 (651, 792)	181·5 (164·6, 199·8)	-37·5 (-44·1, -30·1)
**Barbados**	902 (857, 949)	314·3 (298·5, 330·6)	785 (717, 865)	170·3 (155·6, 187)	-45·8 (-51·4, -40)
**Belize**	247 (228, 268)	250·9 (231·3, 272·9)	553 (521, 588)	193·7 (182·9, 206)	-22·8 (-30·3, -14·4)
**Bermuda**	153 (144, 164)	235·4 (221·1, 251·6)	116 (106, 126)	96·1 (88·1, 104·9)	-59·2 (-63·3, -54·3)
**Cuba**	16599 (16062, 17147)	157·4 (152·3, 162·6)	20054 (17872, 22550)	110·5 (98·6, 124·3)	-29·8 (-37·4, -21·3)
**Dominica**	324 (307, 343)	464·9 (440·7, 490·9)	297 (275, 321)	334·9 (309, 361·1)	-28 (-34·2, -21·2)
**Dominican Republic**	6705 (6133, 7320)	158·6 (145·1, 172·7)	15719 (13363, 18030)	164·6 (140·1, 188·5)	3·7 (-12·9, 22·3)
**Grenada**	213 (201, 226)	310 (292·9, 328·3)	262 (243, 280)	183·6 (170·6, 196·6)	-40·8 (-45·7, -35·2)
**Guyana**	1030 (977, 1084)	239·3 (227, 252·1)	1026 (908, 1153)	156·2 (138·6, 174·8)	-34·7 (-42·5, -25·4)
**Haiti**	20226 (16678, 24114)	556·7 (461·1, 664·4)	26031 (20629, 31890)	352·2 (281, 431·9)	-36·7 (-48·4, -22·4)
**Jamaica**	4972 (4680, 5292)	276·3 (260, 294·6)	5668 (4868, 6613)	197 (169·2, 230·3)	-28·7 (-39·6, -16·6)
**Puerto Rico**	7702 (7411, 7994)	206·2 (198·2, 214·3)	5467 (5064, 5876)	85·2 (78·9, 91·6)	-58·7 (-61·9, -55·2)
**Saint Lucia**	359 (340, 379)	390·2 (370·2, 412)	460 (425, 497)	218·6 (202·2, 235·8)	-44 (-48·9, -38·9)
**Saint Vincent and the Grenadines**	224 (212, 238)	300·3 (284, 318·5)	294 (272, 316)	217·2 (201·4, 233·4)	-27·7 (-34·1, -20·8)
**Suriname**	552 (511, 595)	201·8 (186·8, 216·9)	825 (731, 923)	138 (122·8, 154·3)	-31·6 (-40·6, -21·9)
**Trinidad and Tobago**	1946 (1849, 2050)	218·6 (207·8, 230)	1650 (1359, 1959)	92 (75·7, 109·3)	-57·9 (-65·7, -49·3)
**Virgin Islands**	219 (202, 239)	239·2 (220·6, 259·6)	336 (277, 383)	183·4 (152·3, 208·8)	-23·3 (-36·6, -11)
**Tropical Latin America**	362829 (356305, 369520)	363·5 (356·8, 370·1)	482998 (473844, 492741)	203 (199·3, 207·1)	-44·1 (-45·6, -42·8)
**Brazil**	357204 (350725, 363869)	366·6 (359·8, 373·2)	473063 (463803, 482562)	203·6 (199·6, 207·7)	-44·5 (-45·9, -43·1)
**Paraguay**	5625 (5143, 6117)	237·3 (217·6, 257·3)	9936 (8300, 11932)	180·6 (151·4, 216·5)	-23·9 (-38·1, -7)
**East Asia**	7702886 (7416378, 8143851)	774·9 (747, 820)	8175270 (7834190, 8543708)	389·5 (373·5, 407·2)	-49·7 (-53·3, -46·6)
**China**	7414666 (7139853, 7840195)	787·3 (758·5, 832·7)	7814923 (7475515, 8187412)	391·7 (374·8, 410·2)	-50·3 (-54, -47)
**North Korea**	96601 (78953, 117090)	524·2 (432·9, 627·5)	160354 (126080, 198071)	496·8 (394·6, 612·3)	-5·2 (-28·7, 23·7)
**Taiwan (Province of China)**	63287 (61557, 64980)	361 (351·5, 370·6)	68295 (64051, 73000)	182·7 (171·4, 195·2)	-49·4 (-52·7, -45·7)
**Southeast Asia**	864202 (791291, 936368)	296·9 (272·6, 321·7)	960904 (893460, 1038037)	154·1 (143·5, 166·3)	-48·1 (-53·3, -42·3)
**Cambodia**	24889 (20511, 29773)	480·3 (394·5, 573)	26898 (22871, 32054)	222·8 (190·6, 265·6)	-53·6 (-62·1, -43·4)
**Indonesia**	292447 (259443, 325787)	257·1 (228·1, 286·6)	373166 (337005, 415804)	166·5 (150·6, 185·7)	-35·2 (-43·5, -26·4)
**Laos**	11028 (8839, 13507)	464·4 (374·2, 565·1)	9236 (7273, 11241)	199·3 (163·4, 241·5)	-57·1 (-66·7, -45·1)
**Malaysia**	24121 (21992, 26408)	242·8 (221·5, 266·1)	35627 (31109, 40109)	137 (120, 154)	-43·6 (-53·7, -33·5)
**Maldives**	188 (158, 227)	179·1 (152·2, 211·3)	139 (125, 154)	44·7 (40·3, 49·3)	-75 (-80·1, -69·1)
**Mauritius**	2330 (2214, 2453)	291·7 (276·9, 306·5)	2305 (2116, 2507)	136·6 (125·9, 148·3)	-53·2 (-57·7, -48·5)

**Myanmar**	149152 (120414, 183534)	564·9 (456·3, 694·3)	111183 (93545, 134012)	234·4 (198·8, 281)	-58·5 (-67·9, -48·4)
**Philippines**	52433 (49274, 55868)	152·4 (143·4, 161·9)	71187 (61527, 81765)	91·2 (79·3, 104·1)	-40·2 (-48·9, -30)
**Sri Lanka**	21103 (19519, 22835)	180·6 (167, 195·2)	20658 (17067, 24562)	82·4 (68·3, 97·7)	-54·4 (-63, -44·4)
**Seychelles**	156 (141, 170)	270·3 (244, 295·4)	138 (126, 150)	123·7 (113·3, 133·7)	-54·3 (-60·1, -48·2)
**Thailand**	86008 (79270, 93145)	209·1 (192·3, 226)	99989 (88943, 114310)	102·4 (91·2, 116·5)	-51 (-57·8, -43·3)
**East Timor**	1101 (889, 1347)	309·1 (252·1, 374)	1501 (1153, 1866)	181·2 (142, 223·8)	-41·4 (-55·4, -25)
**Vietnam**	198099 (174140, 223327)	454·7 (399·8, 512)	207614 (177123, 244328)	211·9 (182·7, 247·2)	-53·4 (-62, -43)
**Oceania**	16024 (13276, 18896)	423·8 (356·5, 492·2)	30288 (24766, 36707)	358·4 (297·2, 423·6)	-15·4 (-28·2, -1·9)
**American Samoa**	98 (89, 107)	361·1 (327·1, 396·3)	128 (113, 143)	278·9 (248, 311)	-22·7 (-33·3, -10)
**Federated States of Micronesia**	203 (168, 240)	364·7 (305·5, 427·7)	205 (153, 252)	266 (208·2, 319·4)	-27·1 (-43·6, -8·1)
**Fiji**	630 (555, 708)	145 (128·5, 162·4)	959 (826, 1100)	123·3 (106·5, 141·3)	-15 (-28·7, 1·9)
**Guam**	110 (101, 121)	125·7 (115·6, 137·3)	200 (182, 219)	110·9 (100·9, 121·7)	-11·7 (-22·2, 0·3)
**Kiribati**	157 (141, 172)	361·8 (328, 397·8)	219 (183, 258)	277·3 (236·6, 321·6)	-23·4 (-35·7, -8·5)
**Marshall Islands**	88 (78, 100)	439·3 (386·4, 493·8)	142 (118, 170)	365·6 (306·2, 431·6)	-16·8 (-29·2, -1·8)
**Northern Mariana Islands**	54 (46, 64)	214·9 (186·6, 253·4)	71 (62, 80)	134 (119·4, 150·1)	-37·6 (-49·1, -25·1)
**Papua New Guinea**	12120 (9645, 14692)	508 (412·4, 607·4)	24318 (19119, 30246)	417·3 (335·6, 504·4)	-17·9 (-32·1, -1·9)
**Samoa**	258 (219, 305)	280·3 (237·2, 329·2)	320 (267, 371)	227·7 (192·9, 262·3)	-18·8 (-33·6, -1·8)
**Solomon Islands**	723 (594, 883)	424·9 (350·5, 511·7)	1222 (999, 1481)	312·5 (259·4, 377·1)	-26·4 (-39·7, -9·9)
**Tonga**	221 (202, 242)	378·4 (347·6, 412·5)	252 (221, 291)	304·4 (268·1, 350·2)	-19·6 (-31·9, -4)
**Vanuatu**	305 (230, 403)	383·6 (296·1, 491)	585 (441, 771)	314·6 (241·3, 407·2)	-18 (-36·4, 7·6)
**North Africa and Middle East**	635667 (571681, 693916)	327·2 (295·3, 356)	857927 (809034, 906399)	189 (179, 198·9)	-42·2 (-46·9, -36·5)
**Afghanistan**	68794 (37623, 93199)	933·4 (511·9, 1267·1)	113665 (87457, 146424)	836·1 (664·4, 1033·5)	-10·4 (-30·8, 58·3)
**Algeria**	23364 (20741, 25794)	168·3 (149·6, 185·7)	33303 (30074, 36642)	95·8 (86·6, 105·3)	-43·1 (-49·5, -35·1)
**Bahrain**	467 (434, 504)	223·1 (206·6, 242)	820 (731, 921)	76·8 (68·8, 85·5)	-65·6 (-70·1, -60·7)
**Egypt**	32899 (29899, 36476)	106 (96·8, 116·5)	50308 (44640, 56807)	78·7 (69·8, 88·7)	-25·7 (-36, -13·6)
**Iran**	140206 (128793, 151678)	472·6 (435·6, 510·4)	214818 (207189, 223817)	296·8 (286·3, 308·7)	-37·2 (-41·9, -31·3)
**Iraq**	16332 (13687, 19352)	182 (153·6, 213·9)	18690 (17176, 20529)	68·4 (63·2, 74·4)	-62·4 (-69, -54·3)
**Jordan**	3037 (2607, 3518)	178·8 (154, 206·8)	5507 (4860, 6244)	84·1 (74·3, 95·2)	-53 (-61·8, -43·1)
**Kuwait**	791 (741, 847)	92·8 (86·9, 99·3)	1254 (1136, 1379)	41·6 (37·9, 45·5)	-55·1 (-59·7, -50·2)
**Lebanon**	6722 (5722, 7806)	275·5 (235·1, 317·5)	7950 (7145, 8830)	125·3 (112·7, 139)	-54·5 (-62·3, -44·7)
**Libya**	3435 (2932, 4050)	162·2 (138·9, 190·7)	7464 (6313, 8753)	142·8 (122·4, 164·8)	-12 (-30·3, 9·7)

**Morocco**	18470 (16733, 20518)	119·1 (108·1, 132·3)	26741 (22268, 31594)	82·1 (68·7, 97)	-31·1 (-44, -15·8)
**Palestine**	2259 (1876, 2692)	223·2 (185·4, 265·5)	3421 (3137, 3732)	121·4 (111·4, 132·1)	-45·6 (-56·1, -33·4)
**Oman**	2600 (2125, 3180)	314·9 (257·8, 382·1)	3280 (2695, 3948)	140·4 (116·5, 165·9)	-55·4 (-65·2, -42·8)
**Qatar**	307 (261, 357)	213·2 (183·6, 246·1)	1060 (874, 1287)	95·7 (79·2, 114·7)	-55·1 (-64·3, -43·5)
**Saudi Arabia**	10301 (8499, 12767)	150·5 (123·7, 186·7)	17023 (14712, 20004)	88·7 (78·4, 100·5)	-41·1 (-54·2, -25·3)
**Sudan**	45259 (34336, 56240)	427·6 (326·2, 528·2)	64471 (49586, 80541)	322·2 (247·2, 404·9)	-24·7 (-41·2, -2·2)
**Syria**	8248 (7256, 9235)	138·8 (122·8, 154·8)	12009 (10097, 14149)	87·9 (74·6, 103)	-36·7 (-48·9, -22·2)
**Tunisia**	7469 (6735, 8385)	140·1 (126·9, 156·8)	11987 (9727, 14584)	97·1 (78·9, 118)	-30·7 (-45·1, -12·5)
**Turkey**	211654 (190584, 232893)	525·6 (473·8, 578·1)	192018 (171959, 211043)	216·1 (193·9, 237·3)	-58·9 (-64·2, -52·8)
**United Arab Emirates**	1060 (852, 1291)	201·6 (162·6, 247·6)	7580 (5881, 9484)	174·5 (138·8, 215·2)	-13·4 (-36·2, 18·7)
**Yemen**	31582 (19796, 43312)	531·3 (352, 718·7)	63757 (47491, 82395)	436·9 (328·3, 555·2)	-17·8 (-40·5, 27·4)
**South Asia**	1868954 (1743482, 2009485)	266 (247·9, 285·5)	2626634 (2487690, 2747242)	178·2 (168·5, 186·2)	-33 (-39·4, -27·3)
**Bangladesh**	171018 (147701, 207324)	313·8 (269·9, 376·7)	195045 (165574, 230287)	149·4 (126·7, 176·1)	-52·4 (-64, -40·8)
**Bhutan**	760 (627, 933)	254 (209·5, 311·8)	898 (714, 1100)	133·1 (106·2, 162·9)	-47·6 (-60·4, -33·2)
**India**	1579511 (1462880, 1698510)	276·1 (254·6, 296·5)	2230917 (2107856, 2343723)	187·5 (177·1, 196·9)	-32·1 (-38·4, -25·7)
**Nepal**	32575 (26421, 39913)	289·7 (239·6, 350·9)	42130 (34853, 49964)	183·4 (152·5, 217·4)	-36·7 (-49·8, -20·9)
**Pakistan**	85089 (76169, 95104)	135·7 (122, 151·6)	157644 (130016, 188465)	123·3 (102·2, 146·8)	-9·2 (-26·2, 10·7)
**Southern sub-Saharan Africa**	56013 (52743, 59733)	177·8 (167·2, 189·8)	73206 (69472, 77098)	123·3 (117·3, 129·6)	-30·7 (-35·8, -25·5)
**Botswana**	1147 (955, 1375)	180·5 (152·3, 213·5)	1377 (1206, 1591)	95·8 (84·4, 110·3)	-46·9 (-56·4, -34·9)
**Lesotho**	2662 (2289, 3044)	251·8 (217·5, 287·7)	3043 (2503, 3601)	235·2 (194·3, 276·7)	-6·6 (-24·8, 15·2)
**Namibia**	984 (874, 1106)	127·9 (113·9, 142·5)	973 (832, 1154)	66·7 (57·3, 78·3)	-47·8 (-56·6, -37·4)
**South Africa**	39152 (36536, 41753)	161·6 (150·2, 172·6)	46903 (44914, 49174)	100·9 (96·7, 105·6)	-37·6 (-41·5, -33·1)
**Swaziland**	776 (663, 911)	233·1 (199·5, 270·2)	1127 (928, 1362)	178·6 (147·9, 214·5)	-23·4 (-39·5, -4·5)
**Zimbabwe**	11292 (9856, 12708)	244·7 (213·9, 274·9)	19783 (16912, 22913)	255·5 (220, 293·2)	4·4 (-14·7, 26·6)
**Western sub-Saharan Africa**	212419 (192151, 234225)	220·3 (198·9, 242·8)	323108 (293081, 359119)	167·9 (152·9, 185·7)	-23·8 (-30·9, -14·8)
**Benin**	7076 (6218, 8008)	327·4 (287·8, 370·1)	12327 (10149, 14682)	249·6 (207, 295·7)	-23·8 (-37·6, -7·3)
**Burkina Faso**	16785 (13636, 19590)	355·1 (289·3, 412)	26537 (22690, 31058)	281·5 (240·6, 326·9)	-20·7 (-34·8, 1·6)
**Cameroon**	16405 (14222, 18372)	335·3 (289·4, 376)	30990 (24983, 37301)	254·2 (206·4, 305·8)	-24·2 (-39·1, -8)
**Cape Verde**	1251 (1141, 1363)	556·2 (506·4, 605·7)	1478 (1329, 1632)	333·1 (299·7, 368·7)	-40·1 (-47·7, -31·4)
**Chad**	9013 (7776, 10410)	298 (257·1, 343·6)	16967 (14049, 20031)	296 (245·7, 350·1)	-0·7 (-17·7, 21·6)
**Côte d'Ivoire**	7056 (6078, 8072)	150·4 (131·2, 170·7)	13425 (11336, 15978)	119·4 (101·7, 140·4)	-20·6 (-35·1, -2·7)

**The Gambia**	613 (504, 733)	159·8 (132·7, 189·5)	1167 (991, 1354)	121 (103·7, 139·4)	-24·3 (-38·5, -6·3)
**Ghana**	17130 (14462, 19799)	244·8 (207·7, 281·8)	26790 (23076, 30791)	164 (142, 187·7)	-33 (-45, -18·7)
**Guinea**	12510 (11124, 14076)	344·5 (306·2, 387·4)	20490 (17259, 23925)	348·5 (295·1, 405·8)	1·2 (-17·3, 22·3)
**Guinea-Bissau**	2318 (1920, 2775)	513·5 (427·1, 611·5)	2680 (2236, 3182)	345·9 (292·6, 404·4)	-32·6 (-44·8, -16·9)
**Liberia**	3452 (2977, 4023)	288 (249, 333·6)	4823 (4051, 5688)	232·1 (196·2, 273·6)	-19·4 (-33·8, -1·6)
**Mali**	26671 (23734, 29917)	575·5 (512·6, 644·5)	32380 (26974, 38458)	350·3 (292·5, 412·9)	-39·1 (-49·2, -26·5)
**Mauritania**	3657 (3191, 4164)	336·4 (294·2, 382·7)	4092 (3378, 4900)	200·8 (165·2, 239·4)	-40·3 (-51, -27·4)
**Niger**	10889 (8605, 12980)	338 (269·7, 404·4)	20319 (16246, 24454)	260·6 (211·4, 311·7)	-22·9 (-37·3, -3·9)
**Nigeria**	55095 (41113, 72474)	117·7 (88·3, 152·9)	69661 (49961, 98969)	81·2 (59·4, 112·9)	-31 (-51·1, -2)
**São Tomé and Príncipe**	218 (195, 246)	317 (283·7, 357·1)	350 (288, 421)	332·2 (272·9, 398·7)	4·8 (-16·9, 29·4)
**Senegal**	11475 (10072, 13048)	327·6 (287, 373·6)	19388 (16493, 22555)	259·4 (220·9, 301·6)	-20·8 (-34, -6·1)
**Sierra Leone**	6440 (5028, 7744)	309 (242·2, 370·7)	9802 (8230, 11546)	268 (226·5, 313·5)	-13·3 (-30·4, 15·3)
**Togo**	4359 (3796, 4946)	308·1 (269, 348·6)	9439 (7716, 11380)	251·6 (207·5, 298·7)	-18·3 (-33·1, 0·2)
**Eastern sub-Saharan Africa**	237973 (207534, 270136)	268·3 (235·8, 300·2)	280901 (261364, 301544)	155·8 (145, 167·4)	-41·9 (-48·7, -33·7)
**Burundi**	8732 (7101, 10880)	338·4 (279·7, 418·2)	8886 (7130, 10897)	182·8 (148·9, 221·9)	-46 (-56·2, -34·6)
**Comoros**	633 (531, 758)	270·4 (226·7, 322·3)	745 (617, 898)	151 (125·6, 181·1)	-44·1 (-55·3, -31)
**Djibouti**	440 (321, 606)	228·9 (171·5, 311)	978 (684, 1349)	147·3 (105·3, 197)	-35·6 (-54·2, -9·3)
**Eritrea**	5176 (3805, 6430)	428·7 (336·6, 517·1)	6858 (5591, 8354)	238·7 (197, 284·1)	-44·3 (-55·4, -27·1)
**Ethiopia**	90612 (71088, 114148)	370·2 (295·7, 463)	61993 (54517, 72873)	133·8 (117·7, 157·8)	-63·9 (-71·5, -54·7)
**Kenya**	22750 (19607, 26174)	240·5 (207·4, 275·7)	52970 (47853, 59533)	219·7 (198·9, 247·1)	-8·7 (-17·4, -0·4)
**Madagascar**	14486 (12772, 16407)	239·1 (211·1, 271·3)	21404 (17475, 25694)	168·9 (137·5, 201·4)	-29·4 (-42·9, -13·6)
**Malawi**	5019 (2987, 6268)	113·9 (73·3, 138·9)	5766 (4929, 6661)	72·6 (62·6, 83·4)	-36·2 (-49·9, 1·9)
**Mozambique**	12580 (10754, 14614)	188·9 (162·7, 217·5)	18746 (15655, 21813)	156·9 (132·1, 181·9)	-17 (-32·6, 4·1)
**Rwanda**	11063 (9346, 12834)	327·3 (273·5, 378·7)	8469 (6895, 10084)	131·9 (108·8, 156)	-59·7 (-68·3, -50·3)
**Somalia**	9351 (5284, 13587)	296·2 (188·4, 410·9)	16392 (12355, 21548)	219·2 (166·7, 284)	-26 (-48·1, 18·4)
**South Sudan**	7081 (4437, 10275)	260·8 (174·1, 365·9)	8755 (6467, 11555)	197·8 (148·2, 258·4)	-24·1 (-47·5, 18·1)
**Tanzania**	26083 (19171, 31560)	210·2 (159·1, 252)	36838 (31292, 42834)	138·1 (117·3, 160·9)	-34·3 (-47·3, -12·4)
**Uganda**	13673 (11591, 16075)	183·8 (156·9, 214·9)	19268 (16374, 22331)	124·4 (106·6, 143·1)	-32·3 (-45·7, -15·8)
**Zambia**	10175 (7959, 12303)	298·6 (246·5, 362·9)	12655 (10560, 15097)	168·4 (141·5, 201·7)	-43·6 (-53·7, -30·4)
**Central sub-Saharan Africa**	69972 (59629, 80501)	265·6 (228·1, 305)	100529 (87599, 113903)	173 (151·3, 195)	-34·9 (-44·5, -24)
**Angola**	15836 (12048, 19294)	334·3 (263·2, 401·7)	20157 (16694, 23607)	162·7 (135·2, 190·9)	-51·3 (-61·3, -36·9)

**Central African Republic**	5208<br>(4047, 6251)	379·9<br>(304·5, 450·6)	6838<br>(5267, 8418)	269·1<br>(211·7, 323·4)	-29·2<br>(-42·6, -13)
**Congo**	4105<br>(3541, 4732)	331·7<br>(289·1, 379·4)	5173<br>(4224, 6222)	179<br>(148·6, 210·9)	-46<br>(-57·2, -32·6)
**DR Congo**	42562<br>(34753, 50833)	235·4<br>(194·3, 280)	66282<br>(54421, 78404)	171·8<br>(142·5, 201·5)	-27<br>(-41·5, -9·2)
**Equatorial Guinea**	841<br>(651, 1065)	375·8<br>(295·8, 471·1)	544<br>(368, 784)	103·7<br>(72·8, 146·1)	-72·4<br>(-81·3, -60)
**Gabon**	1420<br>(1152, 1683)	234·2<br>(190·6, 276·6)	1536<br>(1283, 1801)	135·9<br>(115·2, 157·9)	-42<br>(-52·9, -28·4)

Appendix Figure 1. Age-standardised mortality rates of stomach cancer per 100 000 population in 2017, by country and territory

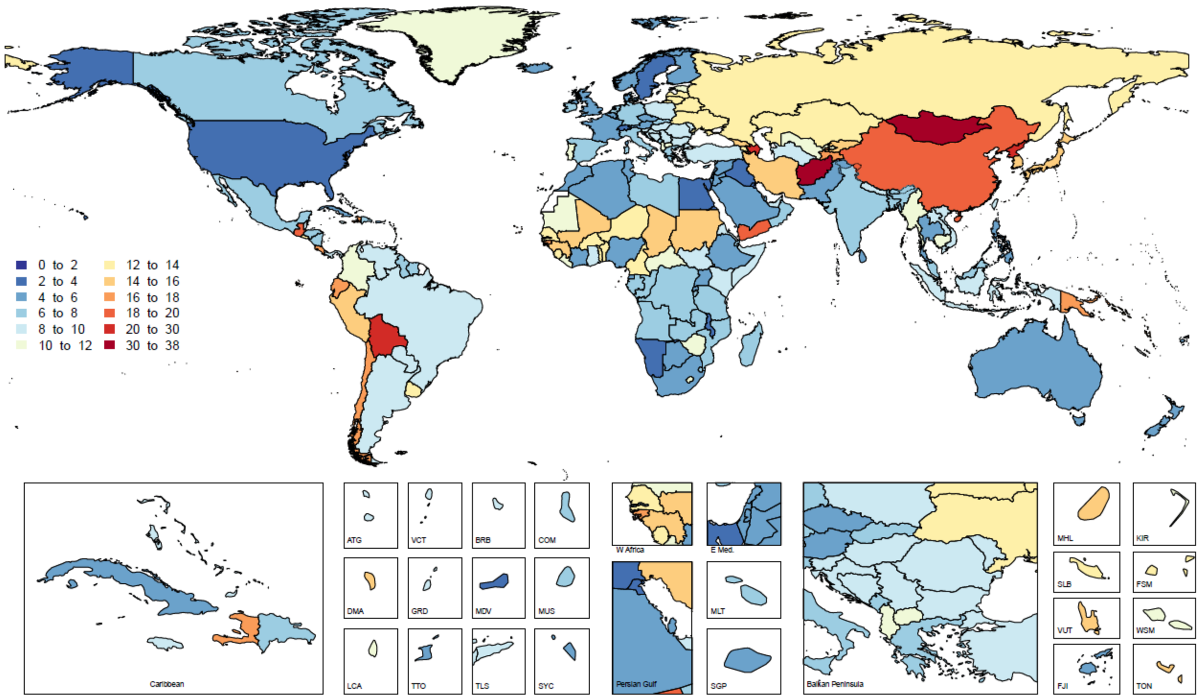

ATG=Antigua and Barbuda, VCT=Saint Vincent and the Grenadines, BRB=Barbados, COM=Comoros, DMA=Dominica, GRD=Grenada, MDV=Maldives, MUS=Mauritius, LCA=Saint Lucia, TTO=Trinidad and Tobago, TLS=Timor-Leste, SYC=Seychelles, MLT=Malta, SGP=Singapore, MHL=Marshall Islands, KIR=Kiribati, SLB=Solomon Islands, FSM=Federated States of Micronesia, VUT=Vanuatu, WSM=Samoa, FJI=Fiji, TON=Tonga.

Appendix Figure 2. Age-standardised DALY rates of stomach cancer per 100 000 population in 2017, by country and territory

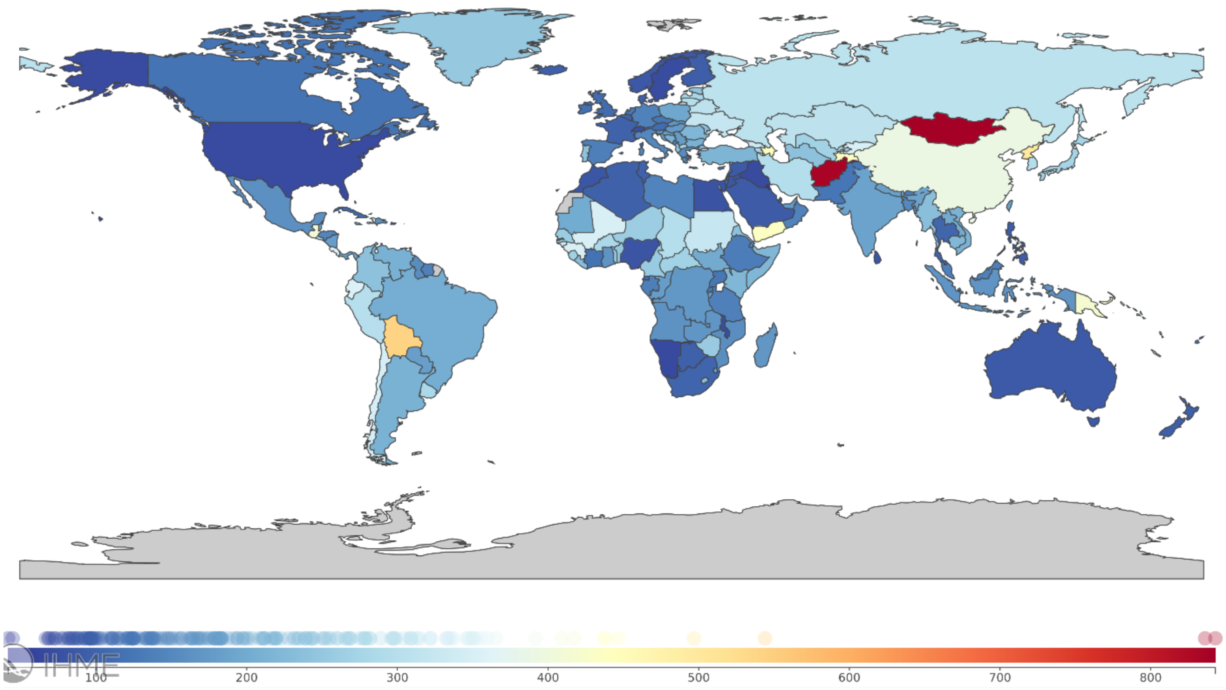

Appendix Figure 3. Global number of incident cases and incidence rates of stomach cancer per 100 000 population by age and sex, 2017

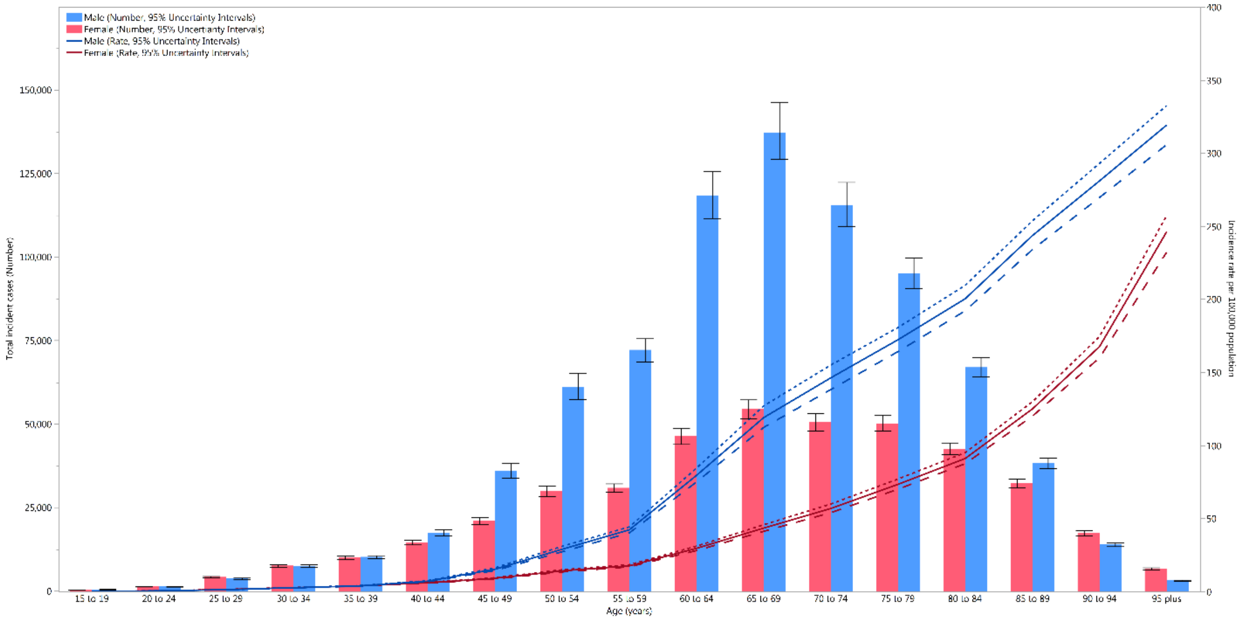

Dotted and dashed lines indicate 95% upper and lower uncertainty intervals, respectively.

Appendix Figure 4. Global number of deaths and death rates of stomach cancer per 100 000 population by age and sex, 2017

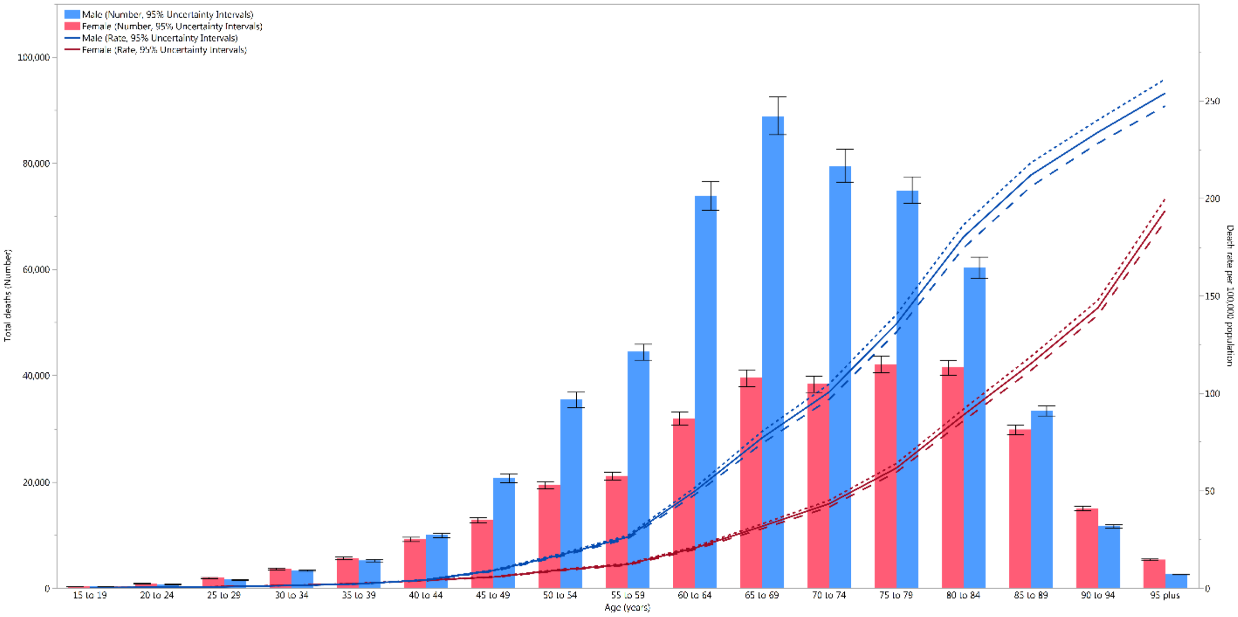

Dotted and dashed lines indicate 95% upper and lower uncertainty intervals, respectively.

Appendix Figure 5. Global number of DALYs and DALY rates of stomach cancer per 100,000 population by age and sex, 2017

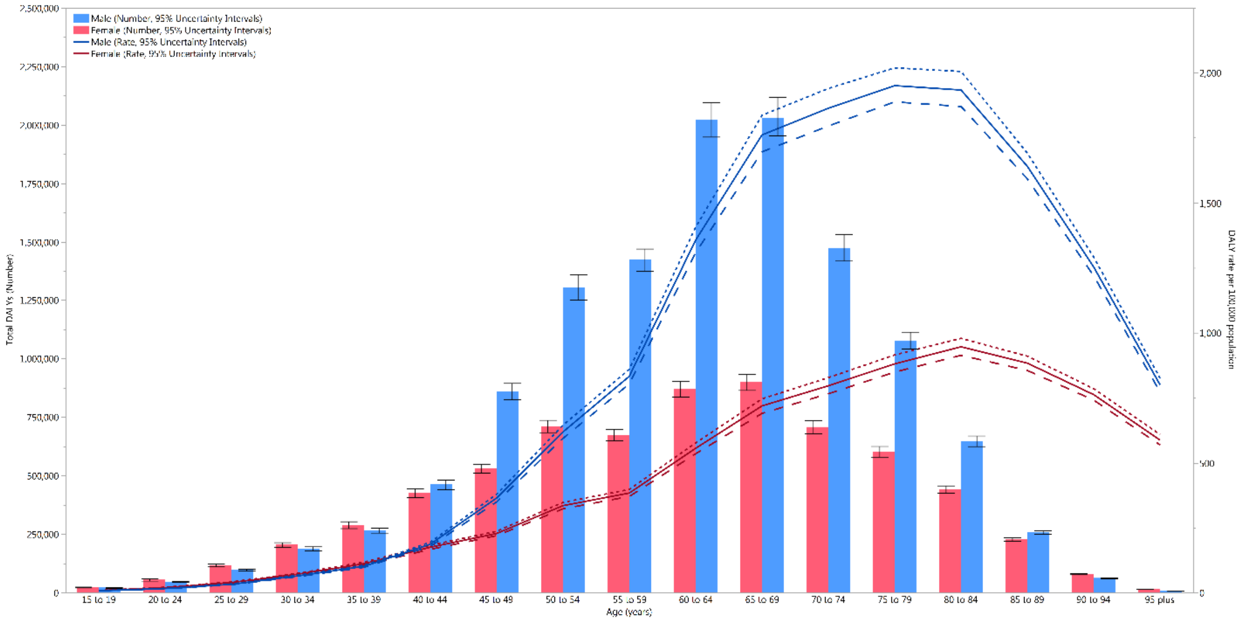

Dotted and dashed lines indicate 95% upper and lower uncertainty intervals, respectively.

Appendix Figure 6. The percentage change in age-standardised incidence rates of stomach cancer from 1990 to 2017 for 21 Global Burden of Disease regions by sex

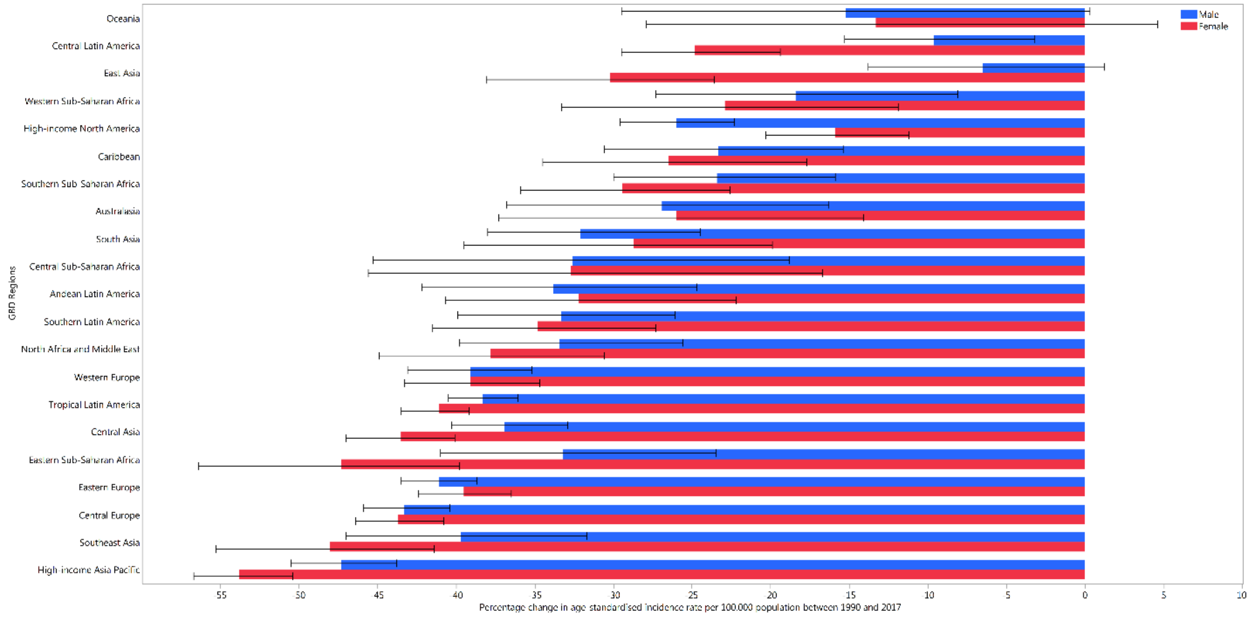

Appendix Figure 7. The percentage change in age-standardised mortality rates of stomach cancer from 1990 to 2017 for 21 Global Burden of Disease regions by sex

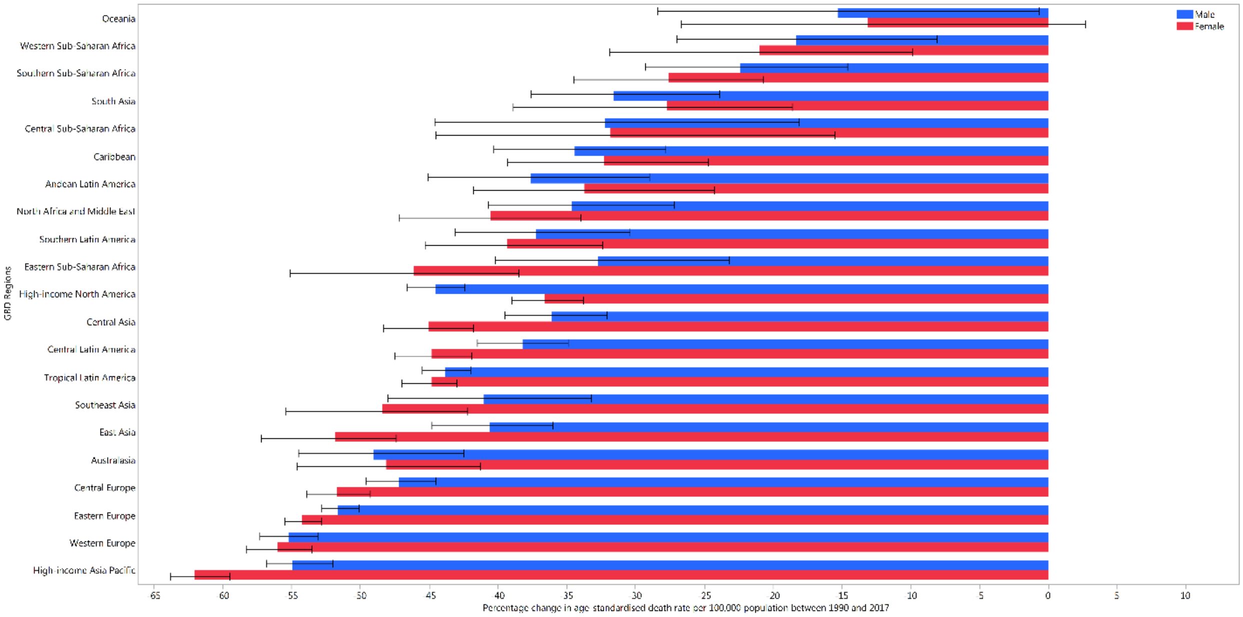

Appendix Figure 8. Age-standardised DALY rates for stomach cancer versus SDI by country and territory, 2017

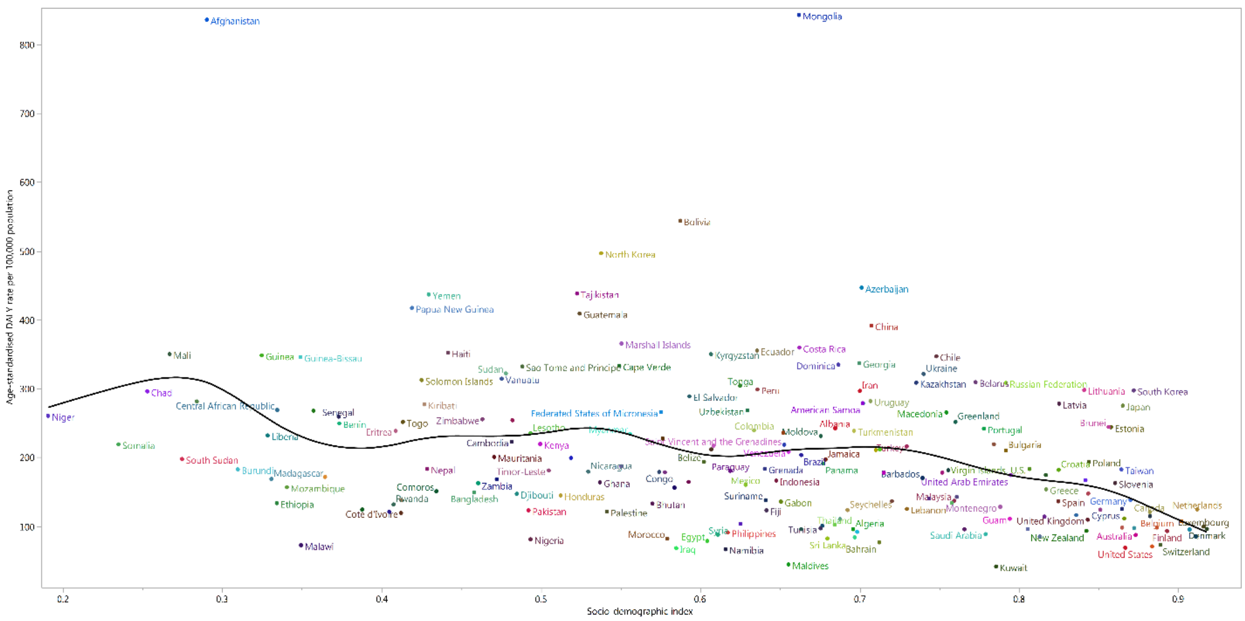

Black line represents the expected age-standardised DALY rate of stomach cancer based solely on SDI. SDI=Socio-demographic Index.

Appendix Figure 9. Percentage of stomach cancer DALYs attributable to high-sodium diet and smoking in 2017, by sex and age

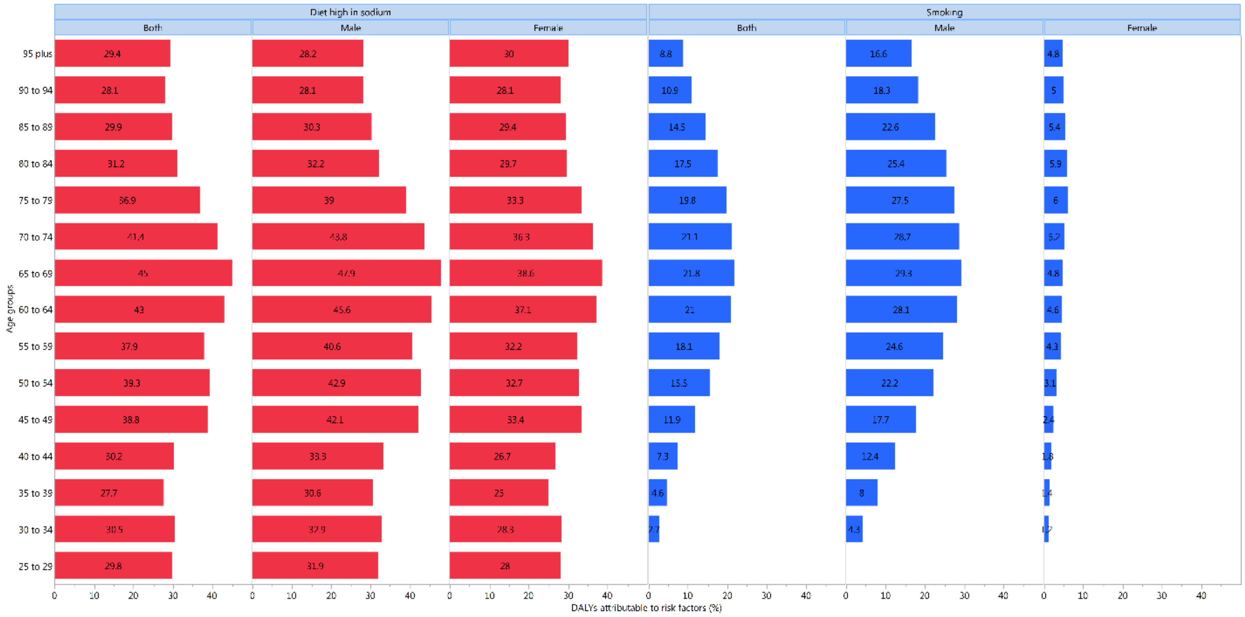

Appendix Figure 10. **Age-standardised (a) incidence, (b) death, and (c) DALY rates of stomach cancer globally and for 21 GBD regions by SDI, 1990-2017.**

Figure 10a

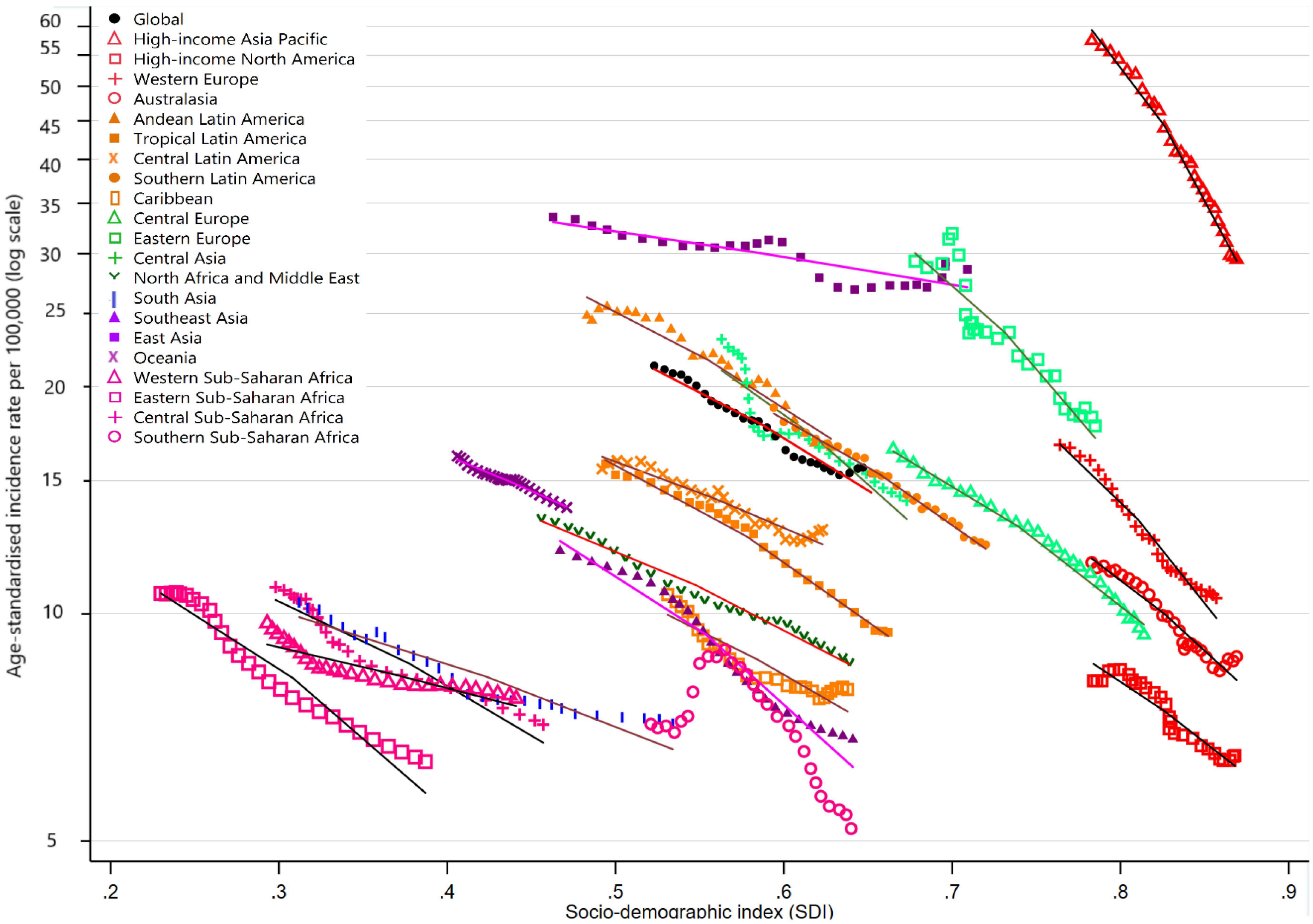

For each region, points from left to right depict estimates from each year from 1990 to 2017. Lines are based on fitted regression models. SDI=Socio-demographic Index. GBD=Global Burden of Diseases, Injuries, and Risk Factors Study.

Figure 10b

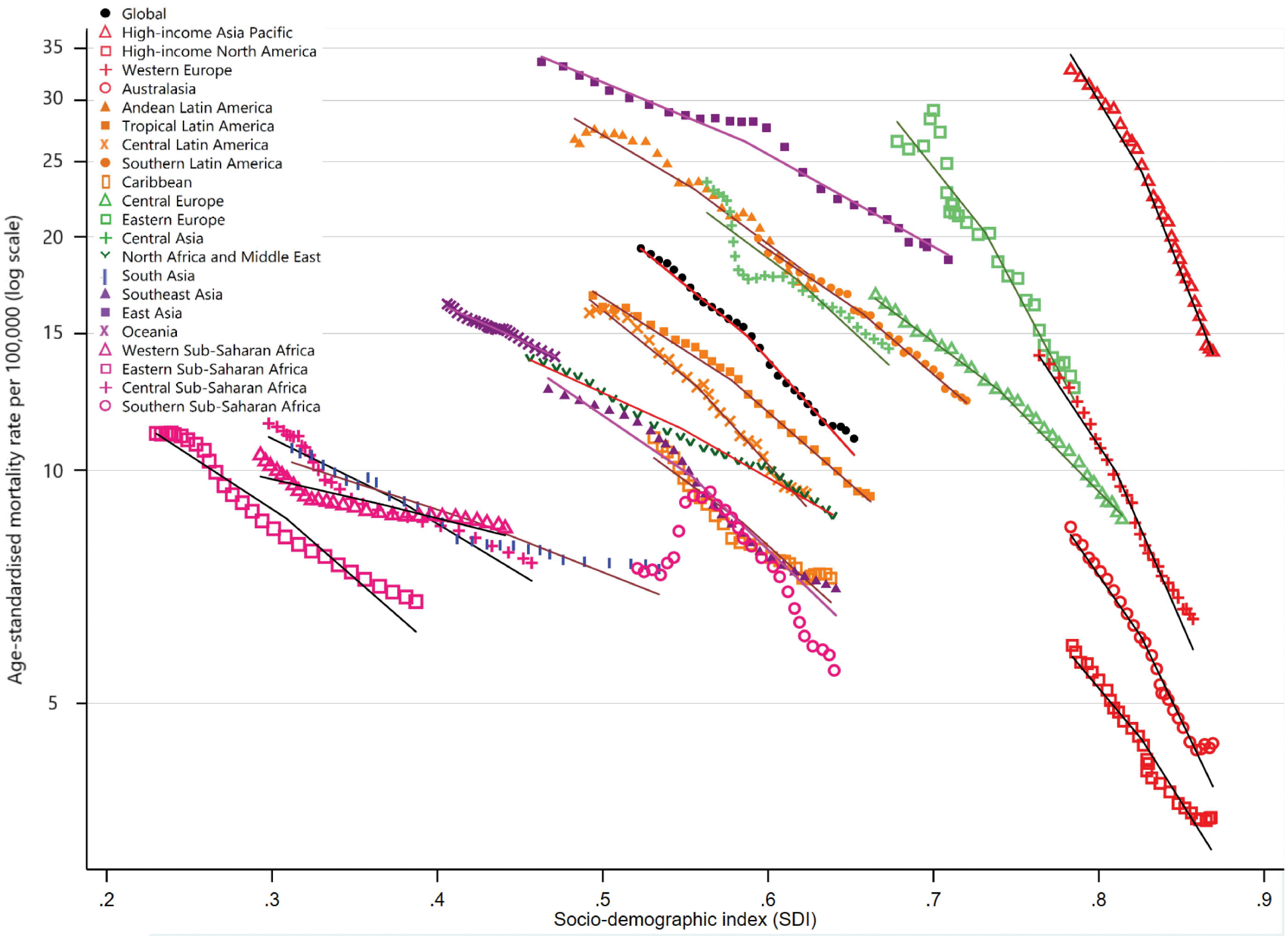

For each region, points from left to right depict estimates from each year from 1990 to 2017. Lines are based on fitted regression models. SDI=Socio-demographic Index. GBD=Global Burden of Diseases, Injuries, and Risk Factors Study.

Figure 10c

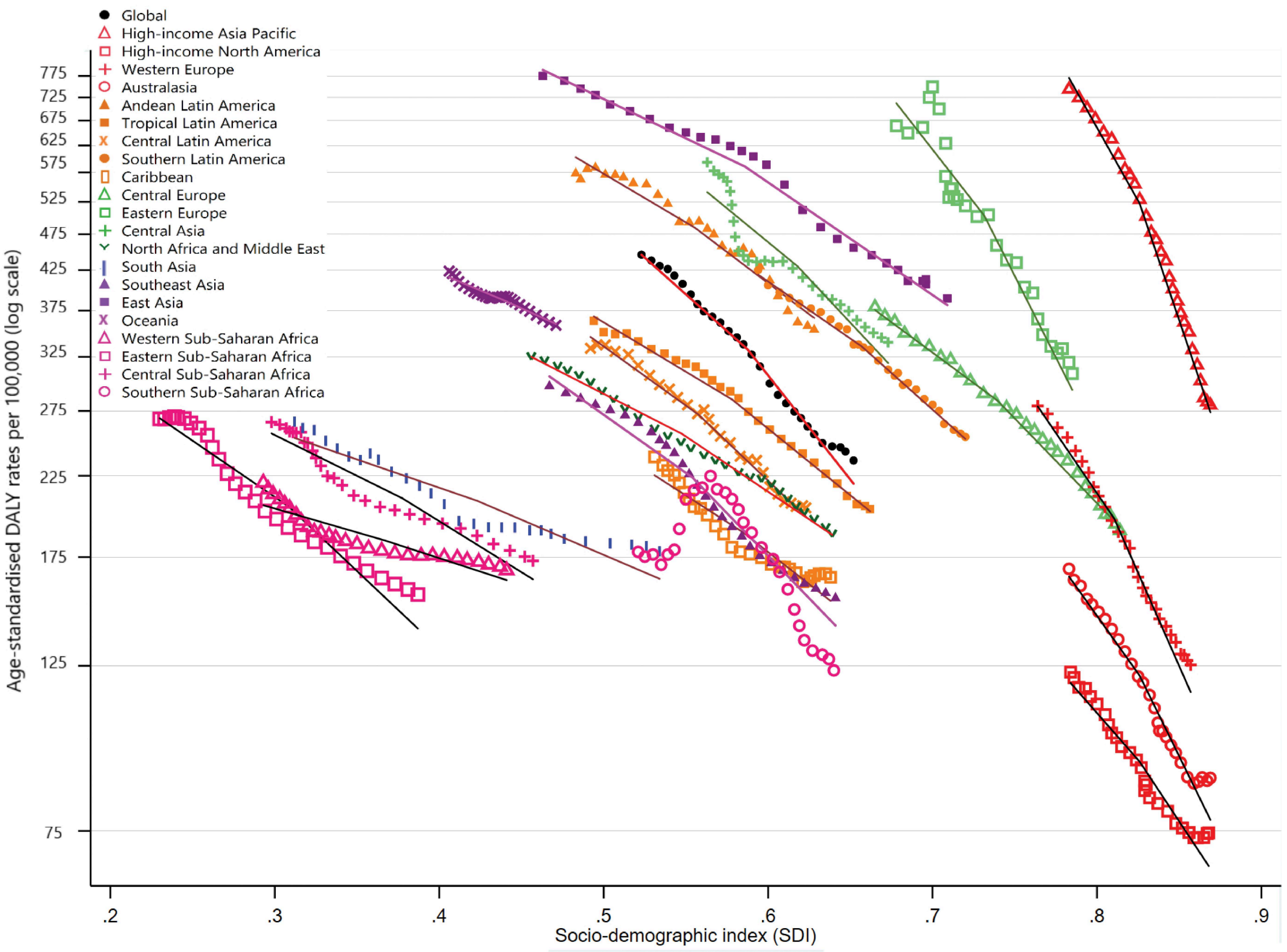

For each region, points from left to right depict estimates from each year from 1990 to 2017. Lines are based on fitted regression models. DALY=disability-adjusted life-year. SDI=Socio-demographic Index. GBD=Global Burden of Diseases, Injuries, and Risk Factors Study.
